# Supplementary material for: Immunogenicity and safety of DS-5670d, an omicron XBB.1.5-targeting COVID-19 mRNA vaccine: A phase 3, randomized, active-controlled study
Source: PLoS Med. 2025 Oct 13;22(10):e1004499. doi: 10.1371/journal.pmed.1004499 (PMC12517495; doi:10.1371/journal.pmed.1004499)
Supplement: S1 Text — (PDF) [file pmed.1004499.s002.pdf]

S1 Text. Tests performed to evaluate the assumptions of the ANCOVA analysis.

We conducted various tests to evaluate the assumptions of the ANCOVA analysis and explore potential biases in terms of (a) multicollinearity of covariates, (b) homogeneity of variance, and (c) homogeneity of regression. None of these tests indicated any evidence that comparisons between DS-5670d and BNT162b were confounded.

(a) Multicollinearity of covariates

We performed ANCOVA analysis with blood neutralizing activity as the dependent variable, study vaccine administration group as an independent variable, and including the following covariates: (i) baseline neutralization titer, subpopulation (i.e., the same covariates as the primary analysis), (ii) subpopulation, (iii) baseline neutralization titer, (iv) none. Regardless of how many covariates were included in the model, the adjusted GMT ratios between DS-5670d and BNT162b2 were not significantly affected. The between-vaccine comparison was robust regardless of multicollinearity among covariates.

	**Adjusted GMT (95% CI)**		**Adjusted GMT ratio (95% CI)**
**Covariates**	**DS-5670d**	**BNT162b2**	**DS-5670d/ BNT162b2**
(i) baseline neutralization titer, subpopulation	1326.489 (1162.071, 1514.170)	1119.097 (976.369, 1282.689)	1.185 (1.025, 1.371)
(ii) subpopulation	1202.703 (1036.650, 1395.354)	950.025 (816.154, 1105.853)	1.266 (1.075, 1.491)
(iii)baseline neutralization titer	1482.734 (1334.577, 1647.339)	1257.340 (1131.228, 1397.511)	1.179 (1.016, 1.369)
(iv) none	1537.643 (1360.761, 1737.517)	1212.088 (1072.133, 1370.313)	1.269 (1.067, 1.508)

(b) Homogeneity of variance

For this evaluation, we conducted Levene's homogeneity of variance test for neutralization titer at Day 29 across treatment groups. We did not detect any statistically significant differences, and this result indicates that the variance has little impact on the comparison between DS-5670d and BNT162b2.

**Factors**	**Degree of freedom**	**Sum of squares**	**Mean square**	**F value**	**Pr > |F|**

Treatment group	1	0.1114	0.1114	0.30	0.5855

(c) Homogeneity of regression

We performed ANCOVA analysis with common logarithm of blood neutralizing activity as the dependent variable, treatment group as an independent variable, and including common logarithm of baseline titer, subpopulation, the interaction of treatment group and subpopulation in the analysis as shown below:

$$Y_{\text{common logarithm of neutralization titer at Day29}} = \beta_0 + \beta_1 X_{\text{treatment\_group(DS5670,Comirnaty)}}$$
$$+\beta_2\ X_{\text{common logarithm of baseline titer}} +$$
$$\beta_3 X_{\text{subpopulation(A,B,C,D)}} + \beta_4 X_{\text{treatment\_group(DS5670,Comirnaty)} \times \text{X\_sub population(A,B,C,D)}}$$

As shown in the table below, we detected statistically significant values between subpopulations, however we did not detect any statistically significant interactions of treatment groups and subpopulation. Therefore, even if heterogeneity in subpopulation exists between treatment groups, this does not have a significant impact on the comparison between DS-5670d and BNT162b2.

**Covariates**	**Degree of freedom**	**Sum of squares**	**Mean square**	**F value**	**Pr > |F|**
Treatment group	1	0.002	0.002	0.01	0.914
Baseline titer	1	40.274	40.274	208.58	<0.001
Subpopulation	3	8.134	2.711	14.04	<0.001
Treatment group × Subpopulation	3	1.277	0.426	2.20	0.086

**Parameter**	**Estimate**	**Standard error**	**t-value**	**Pr > |t|**
Intercept	2.296	0.130	17.69	<0.001
Treatment group: DS-5670d 60 ug	-0.194	0.168	-1.16	0.247
Treatment group: Comirnaty RTU IM	0.000	-	-	-
Baseline titer	0.342	0.024	14.44	<0.001
Subpopulation: A	0.215	0.135	1.60	0.111
Subpopulation: B	0.393	0.138	2.84	0.005
Subpopulation: C	0.068	0.132	0.51	0.609
Subpopulation: D	0.000	-	-	-

Treatment arm * Subpopulation (DS-5670d *A)	0.282	0.176	1.61	0.109
Treatment arm * Subpopulation (DS-5670d *B)	0.145	0.185	0.79	0.432
Treatment arm * Subpopulation (DS-5670d *C)	0.328	0.175	1.87	0.061
Treatment arm * Subpopulation (DS-5670d *D)	0	-	-	-
Treatment arm * Subpopulation (Comirnaty *A)	0	-	-	-
Treatment arm * Subpopulation (Comirnaty *B)	0	-	-	-
Treatment arm * Subpopulation (Comirnaty *C)	0	-	-	-
Treatment arm * Subpopulation (Comirnaty *D)	0	-	-	-
