# Supplementary material for: Immunogenicity and safety of DS-5670d, an omicron XBB.1.5-targeting COVID-19 mRNA vaccine: A phase 3, randomized, active-controlled study
Source: PLoS Med. 2025 Oct 13;22(10):e1004499. doi: 10.1371/journal.pmed.1004499 (PMC12517495; doi:10.1371/journal.pmed.1004499)
Supplement: S2 Text — (PDF) [file pmed.1004499.s003.pdf]

CLINICAL STUDY PROTOCOL

DS5670-237

A PHASE III, RANDOMIZED, ACTIVE-COMPARATOR, OBSERVER-BLIND, NON-INFERIORITY STUDY TO DEMONSTRATE THE IMMUNOGENICITY AND SAFETY OF DS-5670D (MONOVALENT: OMICRON XBB.1.5) IN ADULTS AND CHILDREN AGED 12 YEARS AND OLDER

VERSION 1.0, 20 SEP 2023

VERSION 2.0, 29 FEB 2024

VERSION 3.0, 30 APR 2024

DAIICHI SANKYO

CONFIDENTIALITY STATEMENT

Information contained in this document is proprietary to Daiichi Sankyo. The information is provided to you in confidence which is requested under an agreed upon and signed "Confidentiality and Disclosure Agreement." Do not give this document or any copy of it or reveal any proprietary information contained in it to any third party (other than those in your organization who are assisting you in this work and are bound by the "Confidentiality and Disclosure Agreement") without the prior written permission of an authorized representative of Daiichi Sankyo.

PROTOCOL SYNOPSIS

Protocol Number:	DS5670-237
Investigational Product:	DS-5670d
Active Ingredient/INN:	MAFB-7256a (DS-5670 mRNA)/To be determined.
Study Title:	A Phase III, Randomized, Active-comparator, Observer-blind, Non-inferiority Study to Demonstrate the Immunogenicity and Safety of DS-5670d (Monovalent: Omicron XBB.1.5) in Adults and Children Aged 12 Years and Older
Study Phase:	Phase III
Indication Under Investigation:	Prevention of infection due to severe acute respiratory syndrome coronavirus 2 (SARS-CoV-2)
Study Objectives:	<Primary objective><br>• To verify the non-inferiority of DS-5670d (monovalent: Omicron strain XBB.1.5) (hereinafter, "DS-5670d") to Comirnaty RTU intramuscular injection (monovalent: Omicron strain XBB.1.5) (hereinafter, "Comirnaty RTU IM" unless otherwise noted) in terms of the geometric mean titer (GMT) of blood neutralizing activity against SARS-CoV-2 (Omicron strain XBB.1.5.6) and seroresponse rate at 4 weeks after administration in adults and children aged 12 years and older with at least either a history of infection with SARS-CoV-2 or vaccination with SARS-CoV-2 vaccine.<br><Key secondary objectives><br>• To verify the non-inferiority of DS-5670d to Comirnaty RTU IM in terms of GMT of blood neutralizing activity against SARS-CoV-2 (Omicron strain XBB.1.5.6) and seroresponse rate at 4 weeks after administration in adults and children aged 12 years and older with or without a history of SARS-CoV-2 infection or vaccination with SARS-CoV-2 vaccine.<br>• To evaluate the immunogenicity and safety (excluding primary endpoint) after administration of DS-5670d in adults and children aged 12 years and older with or without a history of SARS-CoV-2 infection or vaccination with SARS-CoV-2 vaccine.
Study Design:	Study type: Interventional<br>Type of intervention: Preventive intervention using a vaccine<br>Type of indication for use: Prevention<br>Study design: A multicenter, randomized, active-comparator, observer-blind, and non-inferiority study<br>Blinding level: Observer-blind<br>Type of control: Active drug<br>Name of active comparator: Comirnaty RTU IM<br>Structure of groups: DS-5670d group and Comirnaty RTU IM group<br>Randomized: Yes<br>Stratification factors: Study site, age (12 to 17 years, 18 to 64 years, 65 years and older), the presence or absence of a history of SARS-CoV-2 infection (yes, no), the presence or absence of a history of vaccination with SARS-CoV-2 vaccine (yes, no)<br>Assignment ratio:<br>DS-5670d group : Comirnaty RTU IM group = 1:1<br>Observation period for subjects: Approximately 26 weeks<br>Whether administered in addition to standard treatment: No
Study Duration:	See Attachment 1 Per-country Requirements.

Study Sites and Location:	Study sites: See "Attachment 3 List of Study Sites and Investigators."<br>Locations: Japan
Subject Eligibility Criteria:	<Inclusion criteria><br>1) Subjects aged 12 years and older at the time of informed consent.<br>2) Subjects who have provided written consent to study participation via the subjects or legal representatives.<br>3) Subjects who are able to comply with the rules of the study, appropriately record symptoms in the electronic diary by the subjects or legal representatives, undergo medical examinations and tests that are specified in the protocol, and report symptoms or other issues (reporting by the legal representatives is also acceptable).<br><br><Exclusion criteria><br>1) Subjects who have any serious cardiovascular, renal, hepatic, blood, neuropsychiatric or developmental disorder, thrombocytopenia, or coagulopathy.<br>2) Subjects who have a history of vaccination-related convulsions or epilepsy.<br>3) Subjects who have a concurrent or medical history of myocarditis or pericarditis.<br>4) Subjects who have tested positive for SARS-CoV-2 infection (based on reverse transcription polymerase chain reaction [RT-PCR], other nucleic acid detection methods, or SARS-CoV-2 antigen test), or have been diagnosed with coronavirus disease 2019 (COVID-19) based on a physician's medical examination within 3 months before informed consent.<br>5) Subjects who have been diagnosed with immunodeficiency in the past or have a close relative with congenital immunodeficiency.<br>6) Subjects who have symptoms suspected of SARS-CoV-2 infection (eg, fever, respiratory symptoms, malaise, headache, gastrointestinal symptoms, nasal discharge/nasal congestion, pharyngeal pain, dysgeusia, olfactory dysfunction, arthralgia, myalgia) at the time of informed consent.<br>7) Subjects whose cohabiting family is determined to be infected with SARS-CoV-2 at the time of informed consent.<br>8) Subjects who have axillary body temperature ≥ 37.5°C at the time of informed consent.<br>9) Subjects who have a serious acute disease at the time of informed consent.<br>10) Subjects who have tested positive for SARS-CoV-2 antigens at the time of eligibility evaluation.<br>11) Subjects who have tested positive for SARS-CoV-2 antibody test at the time of eligibility evaluation, with symptoms suspected of SARS-CoV-2 infection (eg, fever, respiratory symptoms, malaise, headache, gastrointestinal symptoms, nasal discharge/nasal congestion, pharyngeal pain, dysgeusia, olfactory dysfunction, arthralgia, myalgia) within 3 months before informed consent.<br>12) Subjects who have a history of anaphylaxis or severe allergy to food, pharmaceuticals, cosmetics, vaccination, etc.<br>13) Subjects who have received a blood transfusion (including component transfusion) or immunosuppressive therapy (eg,

	radiotherapy) within 30 days before informed consent.<br>14) Subjects who have used gamma globulin preparations, systemic immunosuppressants (including treatments for autoimmune diseases), hematopoietic agents (excluding iron and vitamin preparations), or corticosteroids (excluding external agents, inhalants, and topically administered agents) within 30 days before informed consent.<br>15) Subjects who have participated in another clinical study and received treatment with a drug used in the study (excluding placebo) within 4 months before informed consent.<br>16) Subjects who have received other SARS-CoV-2 vaccine within 3 months before informed consent.<br>17) Subjects who have received other vaccines (excluding influenza vaccines) within 14 days before informed consent.<br>18) Subjects who have previously participated in a clinical trial of this vaccine and have received immunization of DS-5670a, DS-5670b, and DS-5670a/b other than booster immunization of DS-5670a 60 μg and have not completed initial immunization or booster immunization with any other approved SARS-CoV-2 vaccine.<br>19) Subjects who have previously participated in a clinical trial of any other SARS-CoV-2 vaccine and received a drug used in the study other than the approved SARS-CoV-2 vaccine, and have not completed initial immunization or booster immunization with any other approved SARS-CoV-2 vaccine.<br>20) Subjects who are suspected or confirmed as being immunosuppressed or in an immunodeficient state due to infection (eg, human immunodeficiency virus [HIV]), identified in their medical history or medical interview.<br>21) Female subjects who are pregnant or breast-feeding, or who have a positive pregnancy test at the time of eligibility evaluation; premenopausal women of child-bearing potential who have menstruated within 12 months and are not willing to get a pregnancy test; and subjects and subjects' partners who are planning to become pregnant during the study or who are unable to practice effective contraception during the study. Male subjects who intend to donate sperm and female subjects who intend to donate ova during the study.<br>22) Subjects who are otherwise considered unsuitable for the study by the investigator or subinvestigator.
Dosage Form, Dose and Route of Administration:	<Investigational product><br>DS-5670d: A whitish turbid aqueous injection containing MAFB-7256a (DS-5670 mRNA) encoding the receptor-binding domain (RBD) of the spike (S) protein of the Omicron strain XBB.1.5 encapsulated in lipid nanoparticles (LNP) (mRNA encapsulated in LNP [LNP-mRNA]) filled in a colorless glass vial. A solution containing the equivalent of 60 μg of MAFB-7256a in a drug solution of 0.6 mL in a vial (labeled volume: 1.5 mL). A single dose of 0.6 mL (DS-5670d 60 μg as mRNA) will be intramuscularly administered to the deltoid muscle of the upper arm.<br><br><Comparator><br>Comirnaty RTU IM: A frozen aqueous solution containing 0.225 mg of raxtozinameran encapsulated in LNP-mRNA filled in a glass vial (labeled volume 2.25 mL). A single dose of Comirnaty RTU IM at a

	volume of 0.3 mL will be intramuscularly administered to the deltoid muscle of the upper arm.
Study Endpoints:	<u>Efficacy endpoints</u><br><Primary endpoints><br>• GMT of blood neutralizing activity against SARS-CoV-2 (Omicron strain XBB.1.5.6) at 4 weeks after study drug administration in adults and children aged 12 years and older with at least either a history of SARS-CoV-2 infection or vaccination with SARS-CoV-2 vaccine<br>• Seroresponse rate* of blood neutralizing activity against SARS-CoV-2 (Omicron strain XBB.1.5.6) at 4 weeks after study drug administration in adults and children aged 12 years and older with at least either a history of SARS-CoV-2 infection or vaccination with SARS-CoV-2 vaccine<br>*: Percentage of subjects with an increase of ≥ 4 times in the blood neutralizing activity against SARS-CoV-2 after study drug administration, compared with the blood neutralizing activity against SARS-CoV-2 before study drug administration.<br><br><Key secondary endpoints><br>• GMT of blood neutralizing activity against SARS-CoV-2 (Omicron strain XBB.1.5.6) at 4 weeks after study drug administration in adults and children aged 12 years and older with or without a history of SARS-CoV-2 infection or vaccination with SARS-CoV-2 vaccine<br>• Seroresponse rate of blood neutralizing activity against SARS-CoV-2 (Omicron strain XBB.1.5.6) at 4 weeks after study drug administration in adults and children aged 12 years and older with or without a history of SARS-CoV-2 infection or vaccination with SARS-CoV-2 vaccine<br><br><Secondary endpoint><br>• Incidence of COVID-19 for 26 weeks after study drug administration<br><br><u>Safety endpoints</u><br>• Solicited adverse events (AEs) (injection site and systemic)<br>• Unsolicited AEs<br>• Serious adverse events (SAEs)<br>• Clinical laboratory values for 28 days after study drug administration
Planned Sample Size:	Subjects with at least either a history of SARS-CoV-2 infection or vaccination with SARS-CoV-2 vaccine: 690 subjects (DS-5670d group: 345 subjects, Comirnaty RTU IM group: 345 subjects)<br>Among those, subjects with a history of SARS-CoV-2 infection and without a history of vaccination with SARS-CoV-2 vaccine: 100 subjects (DS-5670d group: 50 subjects, Comirnaty RTU IM group: 50 subjects)<br><br>Subjects without a history of SARS-CoV-2 infection or vaccination with SARS-CoV-2 vaccine: Enrolled as many as possible.
Statistical Analyses	<u>Efficacy endpoints</u><br><Primary endpoints><br>To verify the non-inferiority of DS-5670d group to Comirnaty RTU

IM group in adults and children aged 12 years and older with at least either a history of SARS-CoV-2 infection or vaccination with SARS-CoV-2 vaccine. Non-inferiority will have been verified if the following conditions are met simultaneously:

- The lower limit of 2-sided 95% confidence interval of GMT ratio (DS-5670d group vs. Comirnaty RTU IM group) for blood neutralizing activity against SARS-CoV-2 (Omicron strain XBB.1.5.6) at 4 weeks after study drug administration is greater than 0.67.
- The lower limit of 2-sided 95% confidence interval of the difference in seroresponse rate (DS-5670d group − Comirnaty RTU IM group) for blood neutralizing activity against SARS-CoV-2 (Omicron strain XBB.1.5.6) at 4 weeks after study drug administration is greater than −10%.

<Key secondary endpoints>
To verify the non-inferiority of DS-5670d group to Comirnaty RTU IM group in adults and children aged 12 years and older with or without a history of SARS-CoV-2 infection or vaccination with SARS-CoV-2 vaccine. Non-inferiority will have been verified if the following conditions are met simultaneously:

- The lower limit of 2-sided 95% confidence interval of GMT ratio (DS-5670d group vs. Comirnaty RTU IM group) for blood neutralizing activity against SARS-CoV-2 (Omicron strain XBB.1.5.6) at 4 weeks after study drug administration is greater than 0.67.
- The lower limit of 2-sided 95% confidence interval of the difference in seroresponse rate (DS-5670d group − Comirnaty RTU IM group) for blood neutralizing activity against SARS-CoV-2 (Omicron strain XBB.1.5.6) at 4 weeks after study drug administration is greater than −10%.

<Secondary endpoint>

- The incidence of COVID-19 for 26 weeks after study drug administration will be calculated.

Safety endpoints

- The incidence proportion of solicited AEs for 7 days after study drug administration (to be collected from the electronic diary) will be calculated.
- The incidence proportion of unsolicited AEs for 28 days after study drug administration will be calculated.
- The incidence proportion of SAEs from the time of informed consent to 26 weeks after study drug administration will be calculated.
- Summary statistics of laboratory values for 28 days after study drug administration will be calculated.

TABLE OF CONTENTS

1. INTRODUCTION 15
1.1 Background 15
1.2 Study Rationale 16
2. STUDY OBJECTIVES, HYPOTHESES, AND ENDPOINTS 20
2.1 Study Objectives 20
2.1.1 Primary Objective 20
2.1.2 Secondary Objectives 20
2.1.2.1 Key Secondary Objectives 20
2.1.2.2 Other Secondary Objective 20
2.1.3 Exploratory Objectives 20
2.2 Study Hypotheses 20
2.2.1 Primary Hypothesis 20
2.2.2 Key Secondary Hypothesis 21
2.3 Study Endpoints 21
2.3.1 Primary Efficacy Endpoints 21
2.3.2 Secondary Efficacy Endpoints 22
2.3.2.1 Key Secondary Efficacy Endpoints 22
2.3.2.2 Other Secondary Efficacy Endpoint 22
2.3.3 Exploratory Efficacy Endpoints 22
2.3.4 Pharmacokinetic/Pharmacodynamic/Biomarker Endpoints 22
2.3.5 Safety Endpoints 22
3. STUDY DESIGN 23
3.1 Overall Design 23
3.2 Rationale of Study Design 24
4. STUDY POPULATION 27
4.1 Inclusion Criteria 27
4.2 Exclusion Criteria 27
5. STUDY TREATMENTS 30
5.1 Assigning Subjects to Treatments and Blinding 30
5.1.1 Administration Arms/Treatment Sequence 30
5.1.2 Method of Treatment Allocation 30
5.1.3 Blinding 31
5.1.4 Emergency Unblinding Procedure 32
5.2 Drugs Used in the Study 32
5.2.1 Description 32

5.2.2 Labeling and Packaging 33
5.2.3 Preparation 33
5.2.4 Administration 33
5.2.5 Storage 34
5.3 Drugs Used in the Study Other Investigational Drugs 34
5.4 Drug Accountability 34
5.4.1 Study Drugs Supplied by the Sponsor 34
5.4.2 Study Drugs Not Supplied by the Sponsor 35
5.5 Temporary Dose Discontinuation and Reduction 35
5.6 Method of Confirming Administration Compliance 35
5.7 Prior Treatments and Concomitant Therapies 35
5.7.1 Prohibited Concomitant Medication/Therapy 35
5.7.2 Other Restrictions 36
5.8 Subject Withdrawal/Discontinuation 36
5.8.1 Reasons for Withdrawal 37
5.8.2 Withdrawal Procedures 37
5.8.3 Subject Replacement 37
5.8.4 Subject Re-screening Procedures 37
5.9 Study Completion for Individual Subjects 37
6. STUDY PROCEDURES 39
6.1 Informed Consent, Eligibility Evaluation, and Enrollment 39
6.2 Study Drug Administration (Day 1, No Allowance) 40
6.3 Telephone Contact after Study Drug Administration (Day 8, Day 15, Day 22; Allowance ± 2 Days) 41
6.4 Post-dose Evaluation (Day 29, Allowance + 3 Days) 42
6.5 Follow-up Examination (Day 57 [Allowance ± 7 Days], Day 85 [Allowance ± 7 Days], Day 113 [Allowance ± 7 Days], Day 141 [Allowance ± 7 Days], and Day 183 [Allowance ± 14 Days]) 42
6.6 Withdrawal/Discontinuation 43
6.7 Blood Sampling for Immunogenicity Evaluation 43
6.7.1 Sample Handling Methods 44
6.7.2 Labeling for Sample Storage Containers and Shipment Method 44
6.7.3 Evaluation Methods 44
6.8 Electronic Diary 44
6.9 Clinical Laboratory Tests (Blood/Urine) 46
6.10 SARS-CoV-2 Antigen Test 46
6.11 SARS-CoV-2 Antibody Test 46

6.12 Pregnancy Test 47
6.13 Response in the Case of Suspected SARS-CoV-2 Infection 47
6.14 Response in the Case Where Cohabiting Family of Subject Is Determined to Be Infected with SARS-CoV-2 48
7. EFFICACY EVALUATIONS 49
7.1 Method for Evaluation of Efficacy Endpoints 49
7.1.1 Primary Endpoint 49
7.1.1.1 Definition of Seroresponse Rate 49
7.1.2 Secondary Endpoints 49
7.1.2.1 Key Secondary Endpoints 49
7.1.2.2 Other Secondary Endpoints 49
7.1.3 Exploratory Endpoints 50
7.2 Appropriateness of Selected Efficacy Endpoints 50
8. PHARMACOKINETIC/PHARMACODYNAMIC EVALUATIONS 51
8.1 Pharmacokinetic Evaluations 51
8.2 Pharmacodynamic Evaluations 51
8.3 Biomarker Evaluations 51
8.4 Immunogenicity 51
8.5 Pharmacogenetic Analysis 51
9. SAFETY EVALUATION AND REPORTING 52
9.1 Method for Evaluation of Safety Endpoints 52
9.2 Adverse Event Collection and Reporting 52
9.3 Adverse Event 53
9.3.1 Definition of Adverse Event 53
9.3.2 Serious Adverse Events 54
9.3.3 Solicited Adverse Events 54
9.3.3.1 Solicited Injection Site Adverse Events 54
9.3.3.2 Solicited Systemic Adverse Events 55
9.3.4 Unsolicited Adverse Events 55
9.3.5 Severity Evaluation 55
9.3.5.1 Severity of Solicited Injection Site Adverse Events 55
9.3.5.2 Severity of Solicited Systemic Adverse Events 56
9.3.5.3 Severity of Unsolicited Adverse Events 56
9.3.6 Causality Evaluation 57
9.3.7 Action Taken for the Study Drug in Response to Adverse Events 58
9.3.8 Other Action Taken for Adverse Events 58
9.3.9 Adverse Event Outcome 59

9.4 Adverse Events of Special Interest 59
9.4.1 Combined Elevations of Aminotransferases and Bilirubin 59
9.4.2 Antibody Dependent Enhancement 60
9.5 Investigational Device or Investigational Combination Product Defects/Regenerative Medicine Products Defects 60
9.6 Reporting of Serious Adverse Events: Procedures for the Investigator or Subinvestigator 60
9.7 Notifying Regulatory Authorities, Investigators/Subinvestigators, and Institutional Review Boards/Ethics Committees 61
9.8 Exposure in Utero 61
9.9 Clinical Laboratory Evaluations 62
9.10 Vital Signs 62
9.11 Electrocardiograms 62
9.12 Physical Examinations 62
9.13 Other Examinations 63
10. OTHER EVALUATIONS 64
10.1 Patient-Reported Outcome 64
11. STATISTICAL METHODS 65
11.1 General Statistical Considerations 65
11.2 Analysis Sets 66
11.3 Subject Data 68
11.4 Efficacy Analysis 68
11.4.1 Primary Efficacy Analysis 68
11.4.2 Secondary Efficacy Analysis 69
11.4.2.1 Key Secondary Efficacy Analysis 69
11.4.2.2 Additional Secondary Efficacy Analysis 69
11.4.3 Exploratory Efficacy Analysis 69
11.4.4 Pharmacokinetic/Pharmacodynamic Analysis 70
11.4.5 Safety Analysis 70
11.4.5.1 Analysis of Adverse Events 70
11.4.5.2 Analysis of Clinical Laboratory Data 71
11.4.5.3 Analysis of Vital Signs 71
11.4.5.4 Analysis of Electrocardiographic Parameters 71
11.4.6 Blind Review 71
11.5 Interim Analysis 71
11.6 Sample Size Determination 71
11.7 Statistical Analysis Process 73

12. DATA INTEGRITY AND QUALITY ASSURANCE 75
12.1 Monitoring and Inspections 75
12.2 Data Collection....75
12.2.1 Preparation of Case Report Forms....75
12.2.2 Entry Precautions for the Completion of Case Report Forms 76
12.2.3 Format of the Electronic Diary 76
12.3 Data Management 76
12.4 Study Documentation and Storage 77
12.5 Record Keeping 78
13. FINANCING AND INSURANCE 79
13.1 Finances....79
13.2 Payment of Expenses, Compensation for Health Damage, and Insurance 79
14. PUBLICATION OF RESULTS AND DISCLOSURE OF CLINICAL STUDY INFORMATION 80
15. ETHICS AND STUDY ADMINISTRATIVE INFORMATION 81
15.1 Compliance Statement, Ethics, and Regulatory Compliance 81
15.2 Subject Confidentiality 81
15.3 Informed Consent 81
15.4 Regulatory Compliance 82
15.5 Protocol Deviations....83
15.6 Supply of New Information Affecting the Conduct of the Study 83
15.7 Protocol Amendments 84
15.8 Study Termination 84
15.9 Data and Safety Monitoring Board....85
15.10 Address List 85
16. REFERENCES....86
17. APPENDICES....89
17.1 Schedule of Event....89

LIST OF TABLES

Table 9.3-1 Severity of Solicited Injection Site Adverse Events 55
Table 9.3-2 Severity of Solicited Systemic Adverse Events 56
Table 9.3-3 Severity of Unsolicited Adverse Events at the Injection Site (Tenderness) 57
Table 9.3-4 Severity of Unsolicited Systemic Adverse Events (Nausea, Vomiting, Diarrhea) 57
Table 17.1-1 Investigations, Observations, Examinations, and Sampling Schedule....90

LIST OF FIGURES

Figure 3.1-1Study Design.. 24
Figure 5.1-1Subject Enrollment Procedure.. 31

LIST OF ABBREVIATIONS

ABBREVIATION	DEFINITION	
	English	Japanese
ACE2	Angiotensin-converting enzyme 2	アンジオテンシン変換酵素 2
ADE	antibody dependent enhancement	抗体依存性感染増強
ALP	alkaline phosphatase	アルカリフォスファターゼ
ALT	alanine aminotransferase	アラニンアミノトランスフェラーゼ
AST	aspartate aminotransferase	アスパラギン酸アミノトランスフェラーゼ
BUN	blood urea nitrogen	血中尿素窒素
CCG	CRF completion guidelines	症例報告書記入・修正の手引き
CK	creatine kinase	クレアチンキナーゼ
COVID-19	coronavirus disease 2019	新型コロナウイルス感染症
CRF	case report form	症例報告書
CRO	contract research organization	開発業務受託機関
EC	ethics committee	倫理委員会
eCOA	electronic clinical outcome assessment	電子的臨床結果の評価
eCRF	electronic case report form	電子症例報告書
EDC	electronic data capture	電子的データ収集
EIU	EXPOSURE IN UTERO REPORTING FORM	妊娠・出産調査票
EMA	European Medicines Agency	欧州医薬品庁
EU	European Union	欧州連合
EUA	Emergency Use Authorization	緊急使用許可
FAS	full analysis set	最大の解析対象集団
FDA	Food and Drug Administration	米国食品医薬品局
γ-GT	gamma-glutamyltransferase	γ-グルタミルトランスフェラーゼ
GCP	Good Clinical Practice	医薬品の臨床試験の実施の基準
GMFR	geometric mean fold rise	幾何平均上昇倍率
GMT	geometric mean titer	幾何平均抗体価
GPP3	Good Publication Practice for Communicating Company-Sponsored Medical Research	-
HIV	human immunodeficiency virus	ヒト免疫不全ウイルス
ICH	International Council for Harmonisation of Technical Requirements for Pharmaceuticals for Human Use	医薬品規制調和国際会議
ICMJE	International Committee of Medical Journal Editors	医学雑誌編集者国際委員会
ICMRA	International Coalition of Medicines Regulatory Authorities	-
IRB	institutional review board	治験審査委員会
IRT	Interactive response technology	-
LNP	lipid nanoparticle	脂質ナノ粒子
LNP-mRNA	mRNA encapsulated in LNP	LNP に封入した mRNA
MedDRA	Medical Dictionary for Regulatory Activities	ICH 国際医薬用語集
mRNA	messenger ribonucleic acid	メッセンジャーリボ核酸
PCR	polymerase chain reaction	ポリメラーゼ連鎖反応
PMDA	Pharmaceuticals and Medical Devices Agency	独立行政法人医薬品医療機器総合機構
PPS	per-protocol set	治験実施計画書に適合した対象集団
PT	preferred term	基本語

ABBREVIATION	DEFINITION	
	English	Japanese
RBD	receptor-binding domain	受容体結合ドメイン
RNA	ribonucleic acid	リボ核酸
RT-PCR	reverse transcription PCR	逆転写ポリメラーゼ連鎖反応
S	spike	スパイク
SAE	serious adverse event	重篤な有害事象
SAP	statistical analysis plan	統計解析計画書
SARS-CoV-2	severe acute respiratory syndrome coronavirus 2	新型コロナウイルス
SAVER	serious adverse event report	-
SOC	system organ class	器官別大分類
SOP	standard operating procedures	標準業務手順書
SUSAR	suspected unexpected serious adverse reaction	予期せぬ重篤な副反応の疑い
TEAE	treatment-emergent adverse event	治験治療中に発現した有害事象
Th1	T helper cell type 1	ヘルパーT 細胞 1 型
ULN	upper limit of normal	基準値上限
WHO	World Health Organization	世界保健機関

1. INTRODUCTION

1.1 Background

In December 2019, an epidemic outbreak of pneumonia in Wuhan, Hubei Province in the People's Republic of China was reported,[1] and a novel coronavirus was identified as the cause of pneumonia by the Chinese authorities on 09 January 2020.[2] The World Health Organization (WHO) named the disease due to the novel coronavirus (severe acute respiratory syndrome coronavirus 2 [SARS-CoV-2]) as coronavirus disease 2019 (COVID-19) on 11 February 2020[3,4] and indicated that it had reached a pandemic on 11 March 2020.[5] As of 20 August 2023, more than 760 million patients with COVID-19 infection and more than 6.9 million deaths have been reported worldwide, and as of 9 May 2023, more than 33.8 million patients and more than 70 thousand deaths have been reported for COVID-19 infection in Japan.[6,7] Even in the current situation where various modalities of the SARS-CoV-2 vaccine (messenger ribonucleic acid [mRNA], adenoviral vector, recombinant protein, inactivated vaccines, etc.) have been developed and people have been inoculated with these vaccines around the world, and vaccination with mRNA vaccines has also been initiated in Japan, SARS-CoV-2 is repeatedly mutating and the number of new COVID-19 cases continues to be increasing. Thus, the pandemic is not expected to end soon.

Several new variants with different infection, transmission, and antigenic characteristics emerged and were detected in various countries around the world as the results of mutation of the SARS-CoV-2 gene. For Omicron strains, since the first case was reported from South Africa to the WHO on 24 November 2021, the infection has been spreading worldwide. In Japan, the strain was replaced by Omicron subvariant nationwide from around February 2022, and the proportion of XBB.1.5 strains designated as "currently circulating variants of interest" by the WHO as of 27 July 2023 has been increasing since January 2023.[8] According to the nationwide estimate of subvariant detection rate by genomic surveillance based on samples from private laboratories conducted by the National Institute of Infectious Diseases, the rate is estimated to be 38% for XBB.1.16, 35% for XBB.1.9, 9% for XBB.1.5, and 2% for XBB as of 18 July 2023.[9]

The XBB variant has amino acid mutations, including R346T, N460K, and F486S in the receptor binding site of spike (S) protein that plays an important role in the entry of SARS-CoV-2 into host cells, suggesting that immune escape from neutralizing antibody may occur. It has been pointed out that the immune escape similar to that of the XBB variant may occur in the XBB 1.5 variant, and that the binding affinity to angiotensin converting enzyme 2 (ACE2) receptor is higher than that of the XBB variant, resulting in higher infectiveness/transmissibility.[8] Also, it has been pointed out that the XBB.1.16 variant, which is the most dominant variant in Japan, has amino acid mutations of E180V and T478R in the receptor binding site of the S protein in addition to those of the XBB.1.5 variant, and immune escape equivalent to that of the XBB.1.5 variant may occur.[10]

Since the XBB.1.5 variant has been regarded as one of the variants with the highest immune evasion from neutralizing antibodies acquired from the previous vaccination targeting original strain

and COVID-19 infection, the WHO Technical Advisory Group on COVID-19 Vaccine Composition recommends a vaccine targeting the XBB.1.5 variant as one of new vaccine options, particularly for the prevention of symptomatic COVID-19.[11] The Food and Drug Administration (FDA) and the 47th Immunization and Vaccine Committee of the Health Sciences Council in Japan indicated a policy to recommend the use of a monovalent vaccine targeting the XBB.1.5 variant (or XBB.1 variant) as a SARS-CoV-2 vaccine in the fall and winter season of 2023.[12,13] A nonclinical study in mice demonstrated that blood neutralizing activity against SARS-CoV-2 (Omicron strain XBB.1.5) was higher after 2 intramuscular injections of Comirnaty intramuscular injection (Comirnaty IM) targeting the XBB.1.5 variant at a 3-week interval, compared to that after intramuscular injections of Comirnaty RTU IM (bivalent: original strain/Omicron strain BA.4-5).[14]

DS-5670a (monovalent: original strain) (hereinafter, "DS-5670a") and DS-5670d (monovalent: Omicron strain XBB.1.5) (hereinafter, "DS-5670d") are mRNA agents, in which mRNA encodes the receptor-binding domain (RBD), which is a part of the S protein of the original strain and Omicron strain XBB.1.5 detected by our company, encapsulated in lipid nanoparticles (LNP) (mRNA encapsulated in LNP [LNP-mRNA]). Expression of antigen proteins using LNP-mRNA in human body induces appropriate immune response against viruses, i.e., neutralizing antibodies contributing to protection against infection.

Having a development/production ability of mRNA vaccines in Japan is important in the context where a variant-targeting vaccine can be developed immediately after the genetic information of the virus serving as the antigen is clarified and infection control measures can then be taken without foreign influence at times of emerging and re-emerging infections and in preparation for infection-related emergency situations such as pandemic in Japan.[15]

Based on the above background, the development of DS-5670d, a preventive vaccine targeting the RBD of the Omicron strain XBB.1.5 variant as the antigen, was initiated for the purpose of preventing COVID-19 due to the original strain and variants including Omicron strains in adults and children aged 12 years and older.

1.2 Study Rationale

In mice that received DS-5670a intramuscularly, blood anti-RBD IgG titer, blood neutralizing activity against SARS-CoV-2, and RBD-specific cellular immune response overemphasized in T-helper cell type 1 (Th1) were observed.

The results of Part 1 of Study DS5670-146 (a phase I/II/III study to evaluate the effect of booster immunization with DS-5670a in subjects aged 18 years and older who have completed initial immunization with approved SARS-CoV-2 vaccines) confirmed that the geometric mean fold rise (GMFR) of blood neutralizing activity against SARS-CoV-2 (original strain) at 4 weeks after booster immunization in the DS-5670a 60 μg group was higher than that in the comparator group (Comirnaty IM [monovalent: original strain] 30 μg group and Spikevax intramuscular injection [Spikevax IM]

[monovalent: original strain] 50 µg group), regardless of the type of the vaccine for initial immunization. With regard to safety, there were no deaths, serious adverse reactions, or adverse events (AEs) that led to permanent discontinuation of study treatment, with no clear difference in the incidence proportion of solicited AEs in the DS-5670a 60 µg group compared with the comparator group, which resulted in the selection of 60 µg as the recommended non-inferiority study dose for booster immunization with DS-5670a in adults and elderly people.

The results of Part 2 of the above study showed that the lower limit of the 2-sided 97.5% confidence interval of the ratio (DS-5670a 60 µg group/comparator group) of the GMFR of blood neutralizing activity against SARS-CoV-2 (original strain) at 4 weeks after study drug administration is greater than the non-inferiority margin of 0.67, regardless of the type of the vaccine for initial immunization, which verified the non-inferiority of DS-5670a to the comparator. With regard to safety, there were no deaths or AEs that led to permanent discontinuation of study treatment, with no clear difference in the incidence proportion of solicited injection site AEs, solicited systemic AEs, unsolicited AEs, or serious adverse events (SAEs) between the DS-5670a group and the comparator group, regardless of the type of the vaccine for initial immunization.

In Study DS5670-103 (A Phase III, Randomized, Active-Comparator, Observer-Blind, Non-Inferiority Study of DS-5670a in Adults Aged 18 Years and Older), subjects received 2 doses of 60 µg of DS-5670a 4 weeks apart for initial immunization. Neither the difference in the ratio of the geometric mean titer (GMT) of blood neutralizing activity against SARS-CoV-2 (original strain) 4 weeks after initial immunization nor the seroconversion rate compared to the comparator group (Comirnaty IM [monovalent: original strain] 60 µg group) satisfied the non-inferiority verification criteria for this study. Based on the results of this study, non-inferiority of the DS-5670a group to the comparator group was not verified. As for safety, no AEs leading to death were observed, and an AE that led to permanent discontinuation of study treatment was observed in 1 subject in the DS-5670a group. There was no obvious difference in the incidence proportions of solicited injection site AEs and solicited systemic AEs between the DS-5670a group and the comparator group.

Comirnaty IM (monovalent: original strain) and Spikevax IM (monovalent: original strain), vaccines approved in Japan, have been confirmed to induce blood neutralizing activity against SARS-CoV-2 after two doses, in overseas clinical studies. With regard to the efficacy and safety, no differences of concern were observed by age, sex, race, or ethnicity, and a high disease-preventive effect (≥ 90%) against the original strain has been reported.[16,17] In a case-control study conducted by the National Institute of Infectious Diseases, the efficacy of Comirnaty RTU IM (bivalent: original strain/Omicron strain BA.1), Comirnaty RTU IM (bivalent: original strain/Omicron strain BA.4-5), Spikevax IM (bivalent: original strain/Omicron strain BA.1), and Spikevax IM (bivalent: original strain/Omicron strain BA.4-5) in the epidemic of Omicron strain BA.5 was evaluated, and it was suggested that the disease-preventive effect was comparable to Comirnaty IM (monovalent: original strain).[18] In addition, a study showed that the disease-preventive effects targeting the BA.5 and

XBB.1 variants are comparable.[11] In Japan, Comirnaty IM and Spikevax IM targeting the XBB.1.5 variant has been approved for administration since 20 September 2023.[19,Error! Reference source not found.]

Since the immunogenicity and safety of booster immunization with DS-5670a, which contains mRNA encoding the RBD of the original strain, were confirmed in Study DS5670-146, DS-5670d developed by changing the antigen of DS-5670a to Omicron strain XBB.1.5 is also expected to induce a certain level of seroresponse in individuals with basic immunity. In addition, as with Comirnaty IM that has the same modality, it can be expected to have a certain level of disease-preventive effect due to variants that are currently becoming mainstream.

In response to the situation in which the majority of the population has basic immunity from vaccination with SARS-CoV-2 vaccine or SARS-CoV-2 infection, on 18 April 2023, FDA adopted a revised Emergency Use Authorization (EUA) policy to streamline the vaccination schedule for Comirnaty IM (bivalent: original strain/Omicron strain BA.4-5) and Spikevax IM (bivalent: original strain/Omicron strain BA.4-5) to be given in a single dose for adults and children aged 5 years and older, regardless of a history of SARS-CoV-2 vaccination or SARS-CoV-2 infection.[21] On 1 September 2023, the European Medicines Agency (EMA) announced that the autumn 2023 vaccination schedule for adults and children aged 5 years and older will be a single dose of Comirnaty IM targeting the XBB.1.5 variant regardless of SARS-CoV-2 vaccination history.[22] In the U.K., the Joint Committee on Vaccination and Immunisation also recommended a single dose for individuals with no previous vaccination with SARS-CoV-2 vaccine in autumn 2023 and thereafter.[23] According to an FDA analysis, the immune response obtained after a single dose of Spikevax IM (monovalent: original strain, bivalent: original strain/Omicron strain A.1 or bivalent: original strain/Omicron strain BA.4-5) to individuals aged 6 years and older with a history of SARS-CoV-2 infection was comparable to that obtained after two doses of the vaccine to individuals aged 6 years and older without a history of SARS-CoV-2 infection.[21] In the U.K., an observational study was conducted in subjects aged 12 to 17 years who had SARS-CoV-2 infection confirmed by polymerase chain reaction (PCR) test. The estimated vaccine effect of a single dose of Comirnaty IM (monovalent: original strain, bivalent: original strain/Omicron strain A.1 or bivalent: original strain/Omicron strain BA.4-5) was 18.8% in subjects without a history of SARS-CoV-2 infection, compared to 81.5%, 78.8%, and 79.6% in subjects with a history of SARS-CoV-2 infection with alpha, delta, and Omicron strains, respectively. It was reported that the effect of a single dose of the vaccine on the prevention of symptomatic SARS-CoV-2 (Omicron strain) infection was higher than that in those with no history of infection.[24] Based on these studies, it can be inferred that sufficient immunity is induced by a single dose in individuals with basic immunity due to SARS-CoV-2 infection in addition to those with a history of vaccination with SARS-CoV-2 vaccine, leading to consideration of the shift to single dose mainly in foreign countries where the majority of the people have basic immunity.

The office of the Prime Minister announced that, as of 5 September 2023, more than 69.4% of the

people aged 12 years and older have completed the initial immunization.[25] According to the research by the Novel Coronavirus Response Headquarters, the Ministry of Health, Labour and Welfare, and the National Institute of Infectious Diseases, 42.0% of the people aged 16 and older have anti-N antibodies induced by SARS-CoV-2 virus infection.[26] Therefore, it is considered that most of the people aged 12 years and older already have basic immunity. On the other hand, only 17.1% of the people aged 5 to 11 years completed the initial immunization,[25] and the prevalence of anti-N antibodies has not been made clear. Therefore, it is unknown whether the majority of them have basic immunity. However, as the SARS-CoV-2 infection is prevailing or the vaccination with SARS-CoV-2 vaccine progresses, it is expected that most of them will have basic immunity in the future, similarly to the people aged 12 years and older.

In Japan as in other countries, if the majority of the population have acquired basic immunity due to vaccination with SARS-CoV-2 vaccine or SARS-CoV-2 infection, the policy is likely to be changed to a single dose regardless of the history of SARS-CoV-2 vaccination or SARS-CoV-2 infection. Therefore, a study to obtain the indication of this vaccine as a single dose was to be conducted.

For this background, a clinical study was planned in adults and children aged 12 years and older with or without a history of vaccination with SARS-CoV-2 vaccine in parallel with clinical studies in children aged 5 to 11 years with or without SARS-CoV-2 vaccination history. In the primary analysis, the non-inferiority of DS-5670d to Comirnaty RTU IM (monovalent: Omicron strain XBB.1.5) (hereinafter, "Comirnaty RTU IM" unless otherwise noted) will be verified in adults and children aged 12 years and older with at least either a history of SARS-CoV-2 infection or vaccination with SARS-CoV-2 vaccine in whom a single dose is expected to induce sufficient immunity. In the secondary analysis, the non-inferiority of DS-5670d to Comirnaty RTU IM will be verified in all populations including those without a history of SARS-CoV-2 infection or a history of SARS-CoV-2 vaccination, assuming a society where the vaccine will be administered to some individuals without basic immunity.

2. STUDY OBJECTIVES, HYPOTHESES, AND ENDPOINTS

2.1 Study Objectives

2.1.1 Primary Objective

- To verify the non-inferiority of DS-5670d to Comirnaty RTU IM in terms of GMT of blood neutralizing activity against SARS-CoV-2 (Omicron strain XBB.1.5.6) and seroresponse rate* at 4 weeks after administration in adults and children aged 12 years and older with at least either a history of SARS-CoV-2 infection or vaccination with SARS-CoV-2 vaccine.

*: Percentage of subjects with an increase of ≥ 4 times in the blood neutralizing activity against SARS-CoV-2 after study drug administration, compared with the blood neutralizing activity against SARS-CoV-2 before study drug administration.

2.1.2 Secondary Objectives

2.1.2.1 Key Secondary Objectives

- To verify the non-inferiority of DS-5670d to Comirnaty RTU IM in terms of GMT of blood neutralizing activity against SARS-CoV-2 (Omicron strain XBB.1.5.6) and seroresponse rate at 4 weeks after administration in adults and children aged 12 years and older with or without a history of SARS-CoV-2 infection or vaccination with SARS-CoV-2 vaccine.
- To evaluate the immunogenicity and safety (excluding primary endpoint) after administration of DS-5670d in adults and children aged 12 years and older with or without a history of SARS-CoV-2 infection or vaccination with SARS-CoV-2 vaccine.

2.1.2.2 Other Secondary Objective

- To investigate the incidence of COVID-19 for 26 weeks after study drug administration.

2.1.3 Exploratory Objectives

- To confirm the immunogenicity against the variants excluding Omicron strain XBB.1.5.6 after administration of DS-5670d.
- To confirm the persistent immune activity against the Omicron strain XBB.1.5.6 and other variants after administration of DS-5670d.

2.2 Study Hypotheses

The primary and key secondary hypotheses are set as follows: All hypotheses will be evaluated at a one-sided 2.5% significance level. The key secondary hypothesis will be tested only if the primary hypothesis is verified.

2.2.1 Primary Hypothesis

In adults and children aged 12 years and older with at least either a history of SARS-CoV-2

infection or vaccination with SARS-CoV-2 vaccine, the blood neutralizing activity against SARS-CoV-2 (Omicron strain XBB.1.5.6) after a single dose of DS-5670d is non-inferior to that after a single dose of Comirnaty RTU IM. Non-inferiority will have been verified if the following conditions are met simultaneously:

- The lower limit of 2-sided 95% confidence interval of GMT ratio (DS-5670d group vs. Comirnaty RTU IM group) for blood neutralizing activity against SARS-CoV-2 (Omicron strain XBB.1.5.6) at 4 weeks after study drug administration is greater than 0.67.
- The lower limit of 2-sided 95% confidence interval of the difference in seroresponse rate (DS-5670d group − Comirnaty RTU IM group) for blood neutralizing activity against SARS-CoV-2 (Omicron strain XBB.1.5.6) at 4 weeks after study drug administration is greater than −10%.

2.2.2 Key Secondary Hypothesis

In adults and children aged 12 years and older with or without a history of SARS-CoV-2 infection or vaccination with SARS-CoV-2 vaccine, the blood neutralizing activity against SARS-CoV-2 (Omicron strain XBB.1.5.6) after a single dose of DS-5670d is non-inferior to that after a single dose of Comirnaty RTU IM. Non-inferiority will have been verified if the following conditions are met simultaneously:

- The lower limit of 2-sided 95% confidence interval of GMT ratio (DS-5670d group vs. Comirnaty RTU IM group) for blood neutralizing activity against SARS-CoV-2 (Omicron strain XBB.1.5.6) at 4 weeks after study drug administration is greater than 0.67.
- The lower limit of 2-sided 95% confidence interval of the difference in seroresponse rate (DS-5670d group − Comirnaty RTU IM group) for blood neutralizing activity against SARS-CoV-2 (Omicron strain XBB.1.5.6) at 4 weeks after study drug administration is greater than −10%.

2.3 Study Endpoints

2.3.1 Primary Efficacy Endpoints

- GMT of blood neutralizing activity against SARS-CoV-2 (Omicron strain XBB.1.5.6) at 4 weeks after study drug administration in adults and children aged 12 years and older with at least either a history of SARS-CoV-2 infection or vaccination with SARS-CoV-2 vaccine
- Seroresponse rate of blood neutralizing activity against SARS-CoV-2 (Omicron strain XBB.1.5.6) at 4 weeks after study drug administration in adults and children aged 12 years and older with at least either a history of SARS-CoV-2 infection or vaccination with SARS-CoV-2 vaccine

2.3.2 Secondary Efficacy Endpoints

2.3.2.1 Key Secondary Efficacy Endpoints

- GMT of blood neutralizing activity against SARS-CoV-2 (Omicron strain XBB.1.5.6) at 4 weeks after study drug administration in adults and children aged 12 years and older with or without a history of SARS-CoV-2 infection or vaccination with SARS-CoV-2 vaccine
- Seroresponse rate of blood neutralizing activity against SARS-CoV-2 (Omicron strain XBB.1.5.6) at 4 weeks after study drug administration in adults and children aged 12 years and older with or without a history of SARS-CoV-2 infection or vaccination with SARS-CoV-2 vaccine

2.3.2.2 Other Secondary Efficacy Endpoint

- Incidence of COVID-19 for 26 weeks after study drug administration

2.3.3 Exploratory Efficacy Endpoints

- GMT of blood neutralizing activity against SARS-CoV-2 (Omicron strain XBB.1.5.6) and seroresponse rate at 8 weeks, 12 weeks, 16 weeks, 20 weeks, and 26 weeks after study drug administration
- GMT of blood neutralizing activity* against SARS-CoV-2 and seroresponse rate at 4 weeks, 8 weeks, 12 weeks, 16 weeks, 20 weeks, and 26 weeks after study drug administration

*: Details of variants to be evaluated are specified in a separately prepared plan.

2.3.4 Pharmacokinetic/Pharmacodynamic/Biomarker Endpoints

Not applicable.

2.3.5 Safety Endpoints

- Solicited AEs (injection site and systemic) collected from the electronic diary within 7 days after study drug administration
- Unsolicited AEs within 28 days after study drug administration
- SAEs from the time of informed consent to 26 weeks after study drug administration
- Clinical laboratory values for 28 days after study drug administration

3. STUDY DESIGN

3.1 Overall Design

This is a multicenter, randomized, active-comparator, observer-blind, and non-inferiority study.

The primary objective is to verify the non-inferiority of DS-5670d to Comirnaty RTU IM in terms of GMT of blood neutralizing activity against SARS-CoV-2 (Omicron strain XBB.1.5.6) and seroresponse rate at 4 weeks after administration in adults and children aged 12 years and older with at least either a history of SARS-CoV-2 infection or vaccination with SARS-CoV-2 vaccine.

The key secondary objective is to verify the non-inferiority of DS-5670d to Comirnaty RTU IM in terms of GMT of blood neutralizing activity against SARS-CoV-2 (Omicron strain XBB.1.5.6) and seroresponse rate at 4 weeks after administration in adults and children aged 12 years and older with or without a history of SARS-CoV-2 infection or vaccination with SARS-CoV-2 vaccine.

Study drug (DS-5670d or Comirnaty RTU IM) will be intramuscularly administered once to the deltoid muscle of the upper arm.

Blood samples for immunogenicity evaluations will be collected before study drug administration (Day 1), and visits at 4 weeks (Day 29), 8 weeks (Day 57), 12 weeks (Day 85), 16 weeks (Day 113), 20 weeks (Day 141), and 26 weeks (Day 183) after study drug administration. If subjects are aged 12 to 17 years, blood samples for immunogenicity evaluations will be collected as much as possible at 8 weeks (Day 57), 16 weeks (Day 113), and 20 weeks (Day 141) after study drug administration.

For safety evaluation, solicited AEs (injection site and systemic), unsolicited AEs, SAEs and clinical laboratory values for 28 days after study drug administration will be collected.

The health status of each subject will be confirmed by contacting via telephone after study drug administration (Day 8, Day 15, and Day 22).

The subject or legal representative will observe the subject's health status every day after study drug administration until Day 29 visit. Solicited AEs (injection site and systemic) will be recorded in the electronic diary every day for 7 days after study drug administration (Day 1 to Day 8). Unsolicited AEs will be recorded in the electronic diary by the subject or legal representative if any symptom is observed in the subject for 28 days after study drug administration (Days 1 to 29).

An overview of the study design is shown in Figure 3.1-1.

Figure 3.1-1 Study Design

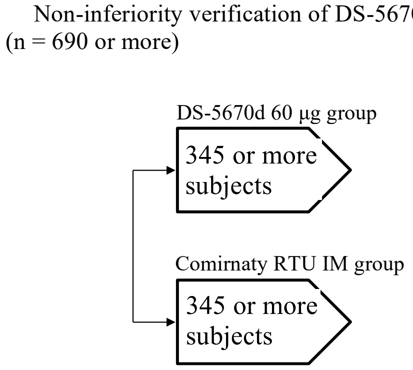

If DS-5670d is approved for marketing by authorities during this study, this study is to be shifted to a post-marketing clinical study.

3.2 Rationale of Study Design

Since the global vaccination with SARS-CoV-2 vaccines has been ongoing worldwide, it is difficult to conduct large-scale clinical studies to confirm the disease-preventive effect of vaccines in comparison with that with placebo. The International Coalition of Medicines Regulatory Authorities (ICMRA) has discussed the clinical studies of SARS-CoV-2 vaccines for future development and has agreed to proceed with the development of these vaccines by alternatively using a randomized, active-comparator, non-inferiority study or superiority study with an indicator of immunogenicity as an endpoint, and this study was therefore designed as an active-controlled, non-inferiority study to evaluate the efficacy and safety of DS-5670d with Comirnaty RTU IM as the comparator.[27]

Khoury et al. reported that a correlation between blood neutralizing activities against SARS-CoV-2 and vaccine effects (disease-preventive effect) has been shown,[28] and the GMT of blood neutralizing activity against SARS-CoV-2 (Omicron strain XBB.1.5.6) was used as an indicator in this study.

As for the comparator, Comirnaty RTU IM (monovalent: Omicron strain XBB.1.5) was selected because it is a variant vaccine for Omicron strain XBB.1.5 developed using Comirnaty IM (monovalent: original strain),[25] the most administered vaccine in Japan, as the parent vaccine and also has the same modality (LNP-mRNA agent) as DS-5670d. A nonclinical study in mice demonstrated that blood neutralizing activity against SARS-CoV-2 (Omicron strain XBB.1.5) was higher after 2 intramuscular injections of Comirnaty RTU IM (monovalent: Omicron strain XBB.1.5) at a 3-week interval, compared to that after intramuscular injections of Comirnaty RTU IM (bivalent: original strain/Omicron strain BA.4-5).[14]

Since Comirnaty IM (monovalent: original strain) is specified to be administered at an interval of at least 3 months from the last dose of the SARS-CoV-2 vaccine for booster immunization to individuals aged 12 years and older who have received SARS-CoV-2 vaccines in the past, this study will enroll subjects for whom 3 months have elapsed since the last booster immunization if they have

a history of vaccination with approved SARS-CoV-2 vaccine.

In Study DS5670-116 (investigation of the safety, tolerability, and recommended dose of DS-5670a in Japanese healthy adults) (phase II study), subjects received 2 doses of DS-5670a 30 μg or 60 μg, 4 weeks apart, for the initial immunization. There were no SAEs or AEs leading to withdrawal from the study. The incidence proportion of solicited injection site AEs was 90.0% (36/40) in both the DS-5670a 30 μg and 60 μg groups, and the incidence proportion of solicited systemic AEs was 62.5 (25/40) and 80.0 (32/40) in the DS-5670a 30 μg and 60 μg groups, respectively. Most of the events were mild or moderate in severity, and all reported events resolved promptly raising no safety concern. The seroconversion rate for blood neutralizing activity against SARS-CoV-2 at 4 weeks after the second dose of DS-5670a was comparable to that at 2 weeks after the second dose. Furthermore, the GMT and GMFR of blood neutralizing activity against SARS-CoV-2 at 2 weeks after the second dose of study drug tended to be higher in the DS-5670a 60 μg group than in the 30 μg group, and 60 μg was selected as the recommended dose for initial immunization with DS-5670a.

In Study DS5670-103 (A Phase III, Randomized, Active-Comparator, Observer-Blind, Non-Inferiority Study of DS-5670a in Japanese Adults Aged 18 Years and Older), subjects received 2 doses of 60 μg of DS-5670a 4 weeks apart for initial immunization. Neither the difference in the ratio of the GMT of blood neutralizing activity against SARS-CoV-2 (original strain) 4 weeks after initial immunization nor the seroconversion rate compared to the comparator group (Comirnaty IM 60 μg group) satisfied the non-inferiority verification criteria for this study. Based on the results of this study, non-inferiority of the DS-5670a group to the comparator group was not verified. As for safety, no AEs leading to death were observed, and an AE that led to permanent discontinuation of study treatment was observed in 1 subject in the DS-5670a group. There was no obvious difference in the incidence proportions of solicited injection site AEs and solicited systemic AEs between the DS-5670a group and the comparator group.

The results of Part 1 of Study DS5670-146 (a phase I/II/III study to evaluate the effect of booster immunization with DS-5670a in Japanese subjects aged 18 years and older who have completed initial immunization with approved SARS-CoV-2 vaccines) confirmed that the GMFR of blood neutralizing activity against SARS-CoV-2 (original strain) at 4 weeks after booster immunization in the DS-5670a 60 μg group was higher than that in the comparator group (Comirnaty IM 30 μg group and Spikevax IM 50 μg group), regardless of the type of the vaccine for initial immunization. With regard to safety, there were no deaths, serious adverse reactions, or AEs that led to permanent discontinuation of study treatment, with no clear difference in the incidence proportion of solicited AEs in the DS-5670a 60 μg group compared with the comparator group, which resulted in the selection of 60 μg as the recommended non-inferiority study dose for booster immunization with DS-5670a in adults and elderly people.

The results of Part 2 of the above study showed that the lower limit of the 2-sided 97.5% confidence interval of the ratio (DS-5670a 60 μg group/comparator group) of the GMFR of blood

neutralizing activity against SARS-CoV-2 (original strain) at 4 weeks after study drug administration is greater than the non-inferiority margin of 0.67, regardless of the type of the vaccine for initial immunization, which verified the non-inferiority of DS-5670a to the comparator. With regard to safety, there were no deaths or AEs that led to permanent discontinuation of study treatment, with no clear difference in the incidence proportion of solicited injection site AEs, solicited systemic AEs, unsolicited AEs, or SAEs between the DS-5670a group and the comparator group, regardless of the type of the vaccine for initial immunization.

Based on the study results, 60 μg was also selected as the dose of DS-5670d for this study.

Since the storage, preparation procedures, and dose of DS-5670d and Comirnaty RTU IM are different, indistinguishability of the study drugs cannot be achieved. Therefore, an observer-blind approach was adopted in this study to eliminate bias to evaluations. In the observer-blind approach, the preparation and administration of the study drug and evaluations will be performed by different persons and the evaluations will be performed in a blinded manner.

In Study DS5670-146, the blood neutralizing activity against SARS-CoV-2 increased at 4 weeks after study drug administration. Therefore, the time point of 4 weeks after study drug administration was selected as the time to evaluate the primary endpoint, considering the time point of safety evaluation for subjects (at 4 weeks after study drug administration) and the convenience for subjects to visit.

In accordance with the "Principles for the Evaluation of Vaccines Against the Novel Coronavirus SARS-CoV-2,"[29] a solicited AE observation period of 7 days and an AE observation period of 28 days after administration of DS-5670d were included to collect information on specific injection site reactions and specific systemic reactions occurring for at least 7 days and safety events occurring for at least 28 days after SARS-CoV-2 vaccine administration. In addition, a follow-up period of 52 weeks after study drug administration was included to collect information on long-term efficacy and safety including antibody dependent enhancement (ADE) after SARS-CoV-2 vaccine administration.

In Japan as in other countries, if the majority of the population have acquired basic immunity due to vaccination with SARS-CoV-2 vaccine or SARS-CoV-2 infection, the policy is likely to be changed to a single dose regardless of the history of SARS-CoV-2 vaccination or SARS-CoV-2 infection. Therefore, a study to obtain the indication of this vaccine as a single dose was to be conducted. In the primary analysis, the non-inferiority of DS-5670d to Comirnaty RTU IM will be verified in adults and children aged 12 years and older with at least either a history of SARS-CoV-2 infection or vaccination with SARS-CoV-2 vaccine in whom a single dose is expected to induce sufficient immunity. In the secondary analysis, the non-inferiority of DS-5670d to Comirnaty RTU IM will be verified in all populations including those without a history of SARS-CoV-2 infection or a history of SARS-CoV-2 vaccination, assuming a society where the vaccine will be administered to some individuals without basic immunity.

After issuance of the notification from the Pharmaceutical Evaluation Division dated March 4,

2024, "Modification of Description on Dosage and Administration of COVID-19 Vaccines,"[30] modification of Dosage and Administration of various COVID-19 vaccines was instructed to describe mainly the acquisition of booster immunization to adjust to changes in a social environment. On April 1, 2024, the package inserts of Comirnaty RTU IM (monovalent: Omicron strain XBB.1.5) and Spikevax IM (monovalent: Omicron strain XBB.1.5) were revised to modify Dosage and Administration mainly for the acquisition of booster immunization. So, a single dose became possible regardless of a history of SARS-CoV-2 vaccination or a history of SARS-CoV-2 infection. The package insert of DS-5670d is very likely to go through similar modification before completion of this study, so the 52-week follow-up period after administration of the study drug was modified to 26 weeks for ethical consideration to subjects.

4. STUDY POPULATION

4.1 Inclusion Criteria

Subjects must satisfy all of the following criteria to be included in the study:

1) Subjects aged 12 years and older at the time of informed consent.
2) Subjects who have provided written consent to study participation via the subjects or legal representatives.
3) Subjects who are able to comply with the rules of the study, appropriately record symptoms in the electronic diary by the subjects or legal representatives, undergo medical examinations and tests that are specified in the protocol, and report symptoms or other issues (reporting by the legal representatives is also acceptable).

<Rationale>

1) This was included to appropriately evaluate the efficacy and safety in adults and children.
2) This was included in accordance with Good Clinical Practice (GCP).
3) This was included to appropriately evaluate the safety and immunogenicity of the study drug.

4.2 Exclusion Criteria

Subjects who meet any of the following criteria will be excluded from the study:

1) Subjects who have any serious cardiovascular, renal, hepatic, blood, neuropsychiatric or developmental disorder, thrombocytopenia, or coagulopathy.
2) Subjects who have a history of vaccination-related convulsions or epilepsy.
3) Subjects who have a concurrent or medical history of myocarditis or pericarditis.
4) Subjects who have tested positive for SARS-CoV-2 infection (based on reverse transcription PCR [RT-PCR], other nucleic acid detection methods, or SARS-CoV-2 antigen test), or have been diagnosed with COVID-19 based on a physician's medical

examination within 3 months before informed consent.

5) Subjects who have been diagnosed with immunodeficiency in the past or have a close relative with congenital immunodeficiency.
6) Subjects who have symptoms suspected of SARS-CoV-2 infection (eg, fever, respiratory symptoms, malaise, headache, gastrointestinal symptoms, nasal discharge/nasal congestion, pharyngeal pain, dysgeusia, olfactory dysfunction, arthralgia, myalgia) at the time of informed consent.
7) Subjects whose cohabiting family is determined to be infected with SARS-CoV-2 at the time of informed consent.
8) Subjects who have axillary body temperature ≥ 37.5°C at the time of informed consent.
9) Subjects who have a serious acute disease at the time of informed consent.
10) Subjects who have tested positive for SARS-CoV-2 antigens at the time of eligibility evaluation.
11) Subjects who have tested positive for SARS-CoV-2 antibody test at the time of eligibility evaluation, with symptoms suspected of SARS-CoV-2 infection (eg, fever, respiratory symptoms, malaise, headache, gastrointestinal symptoms, nasal discharge/nasal congestion, pharyngeal pain, dysgeusia, olfactory dysfunction, arthralgia, myalgia) within 3 months before informed consent.
12) Subjects who have a history of anaphylaxis or severe allergy to food, pharmaceuticals, cosmetics, vaccination, etc.
13) Subjects who have received a blood transfusion (including component transfusion) or immunosuppressive therapy (eg, radiotherapy) within 30 days before informed consent.
14) Subjects who have used gamma globulin preparations, systemic immunosuppressants (including treatments for autoimmune diseases), hematopoietic agents (excluding iron and vitamin preparations), or corticosteroids (excluding external agents, inhalants, and topically administered agents) within 30 days before informed consent.
15) Subjects who have participated in another clinical study and received treatment with a drug used in the study (excluding placebo) within 4 months before informed consent.
16) Subjects who have received other SARS-CoV-2 vaccine within 3 months before informed consent.
17) Subjects who have received other vaccines (excluding influenza vaccines) within 14 days before informed consent.
18) Subjects who have previously participated in a clinical trial of this vaccine and have received immunization of DS-5670a, DS-5670b, and DS-5670a/b other than booster immunization of DS-5670a 60 μg and have not completed initial immunization or booster immunization with any other approved SARS-CoV-2 vaccine.
19) Subjects who have previously participated in a clinical trial of any other SARS-CoV-2

vaccine and received a drug used in the study other than the approved SARS-CoV-2 vaccine, and have not completed initial immunization or booster immunization with any other approved SARS-CoV-2 vaccine.

20) Subjects who are suspected or confirmed as being immunosuppressed or in an immunodeficient state due to infection (eg, human immunodeficiency virus [HIV]), identified in their medical history or medical interview.
21) Female subjects who are pregnant or breast-feeding, or who have a positive pregnancy test at the time of eligibility evaluation; premenopausal women of child-bearing potential who have menstruated within 12 months and are not willing to get a pregnancy test; and subjects and subjects' partners who are planning to become pregnant during the study or who are unable to practice effective contraception during the study. Male subjects who intend to donate sperm and female subjects who intend to donate ova during the study.
22) Subjects who are otherwise considered unsuitable for the study by the investigator or subinvestigator.

<Rationale>

1), 2), 3), 5), 8), 9), 12) These were included in consideration of the safety of subjects and to ensure the eligibility of subjects.

4), 11), 13), 14), 20) These were included in consideration of the safety of subjects and influence on immunogenicity and to ensure the eligibility of subjects.

6), 7), 10) These were included to remove the potential risks of SARS-CoV-2 infection.

15), 17) These were included in consideration of the safety of subjects and influence of other medications.

16), 18), 19) There were included to appropriately evaluate the immunogenicity of the study drug.

21) This was included in consideration of the influence on the subject, subject's partner, fetuses, and newborn.

22) This was included to allow the investigator or subinvestigator to make judgments in consideration of other general factors.

5. STUDY TREATMENTS

5.1 Assigning Subjects to Treatments and Blinding

5.1.1 Administration Arms/Treatment Sequence

Investigational product group

DS-5670d: A single dose of 0.6 mL (DS-5670d 60 µg as mRNA) will be intramuscularly administered to the deltoid muscle of the upper arm.

Comparator group

Comirnaty RTU IM: A single dose of Comirnaty RTU IM at a volume of 0.3 mL will be intramuscularly administered to the deltoid muscle of the upper arm.

5.1.2 Method of Treatment Allocation

The independent biostatistician will prepare a subject randomization list showing a correspondence between subject numbers and study drugs (investigational product or comparator).

Subjects will be randomized in a 1:1 ratio to the DS-5670d group or Comirnaty RTU IM group, with study site, age (12 to 17 years, 18 to 64 years, 65 years and older), the presence or absence of a history of SARS-CoV-2 infection (yes, no), the presence or absence of a history of vaccination with SARS-CoV-2 vaccine (yes, no) as stratification factors based on the information entered in the Interactive Response Technology (IRT). The presence or absence of a history of SARS-CoV-2 infection used as a stratification factor will be based on the history of SARS-CoV-2 infection reported by the subject or legal representative at informed consent (self-reported SARS-CoV-2 infection history) and the SARS-CoV-2 antibody test result from the rapid test kit at the time of eligibility evaluation. A subject will be defined as having a history of SARS-CoV-2 infection if he/she is determined to have a self-reported SARS-CoV-2 infection history at informed consent or have a positive SARS-CoV-2 antibody with the rapid test kit at the time of eligibility evaluation. Otherwise, the subject will be defined as having no history of SARS-CoV-2 infection.

The procedures for subject enrollment are shown in Figure 5.1-1.

After obtaining written consent from the subject or subject's legal representative, the investigator or subinvestigator will prepare a subject screening list, and confirm the eligibility of the subject.

The investigator, subinvestigator, or study staff will register the required information in the IRT after obtaining informed consent. The IRT will allocate a study-specific subject number. The investigator, subinvestigator, or study staff will enter confirmation of the subject eligibility in the IRT. The IRT will randomize the eligible subject to an administration and notification of the assigned study drug will be sent to the unblinded personnel at the study site (See Section 5.1.3).

After the unblinded personnel at the study site (study drug manager or study drug management assistant designated by the study drug manager, study drug preparer, and study drug administration personnel) will confirm the assigned study drug based on the IRT notification, the assigned study

drug will be dispensed, prepared and administered in a separate location from the blinded personnel. The date of the randomization is defined as the enrollment date.

If the subject is determined ineligible, the investigator or subinvestigator will provide the subject and, if necessary, his/her legal representative with the reason for the ineligibility. The investigator or subinvestigator will also record the reason for not enrolling the subject in the subject screening list.

Figure 5.1-1 Subject Enrollment Procedure

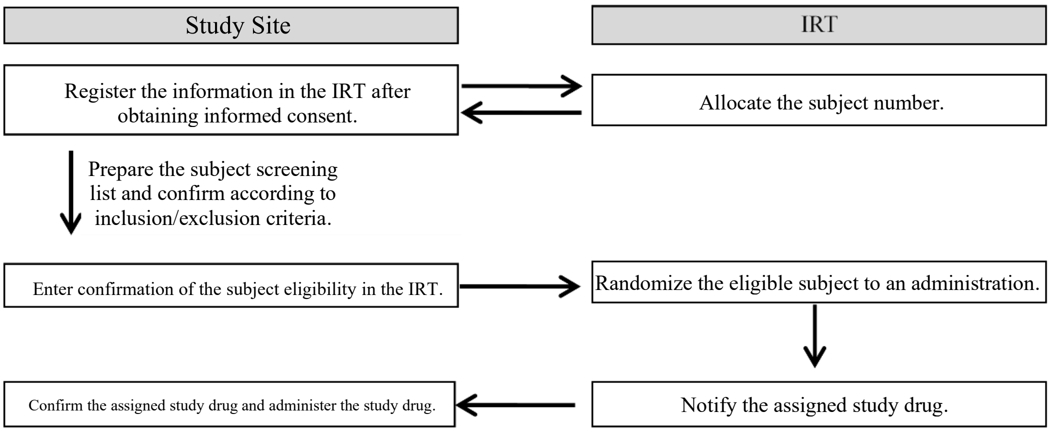

5.1.3 Blinding

In this study, the "observer-blind approach" is adopted (the reason for using this approach is detailed in Section 3.2 "Rationale of Study Design").

Unblinding will be applied to the independent biostatistician, study drug manager or study drug management assistant (designated by the study drug manager), study drug preparer, study drug administration personnel, unblinded study staff (if needed), and unblinded sponsor personnel (including unblinded monitor). Blinding will be applied to subjects, legal representatives, investigator, subinvestigator, study staff, nurses, monitors, the sponsor, and personnel measuring antibody titers.

The unblinded and blinded personnel must keep independent of each other. The unblinded personnel must not disclose information to maintain the blinding to the blinded personnel. An unblinded personnel cannot have the role of the blinded personnel.

When documenting assignment of each subject in the source documents, etc., the unblinded personnel should make sure that the administered study drug is not identifiable. If the blinded personnel become aware of the subject's assignment details, the sponsor personnel will be notified immediately.

5.1.4 Emergency Unblinding Procedure

In the case of an emergency where, in the opinion of the investigator or subinvestigator, the study administration must be unblinded in order to evaluate a course of medical treatment, the investigator or subinvestigator can perform the unblinding by using the IRT. Entry of the AE electronic case report form (eCRF) page must be completed before the unblinding except in cases where immediate treatment of the subject is required. If immediate treatment is required, the AE eCRF page is to be completed within 24 hours of the unblinding.

In the event of an unblinding, the subject and designated study site staff/personnel who are providing immediate care to the subject will be informed about their study drug assigned. The unblinding results must not be included in the subject's source documents to ensure the treatment assignment will remain blinded to monitor and other study personnel not involved with the subject's immediate care.

In the event of an unblinding, an automatic notification (via e-mail) will be sent to the investigator or subinvestigator and selected sponsor personnel from the IRT vendor. The notification will contain the name of a person who broke the blinding, the date and time of unblinding, and the subject number. The notification will not contain any unblinding information.

Once study treatment has been unblinded for a specific subject, the investigator or subinvestigator should treat the subject appropriately. The investigator or subinvestigator will perform evaluations at the end of study drug administration and follow-up evaluations for the subject in accordance with the protocol.

5.2 Drugs Used in the Study

"Drugs used in the study" refer to drugs (including investigational product) containing the approved active ingredient or unapproved active ingredients specified in the clinical trial plan notification and this protocol that are used to evaluate the efficacy and safety of the investigational product in the study. Specifically, study drugs (investigational product and comparator), concomitant medications, rescue drugs, premedication, etc. are included.

Drugs used in the study are the investigational product (DS-5670d) and the comparator (Comirnaty RTU IM). Since drugs other than the study drugs will not be used in the study, the "drug used in the study" is described as "study drug" in this protocol.

5.2.1 Description

Investigational product

DS-5670d

1) Test substance identification code: DS-5670d
2) Generic name: To be determined.
3) Content and dosage form:

A whitish turbid aqueous injection containing MAFB-7256a (DS-5670 mRNA) encoding the RBD of the S protein of the Omicron strain XBB.1.5 encapsulated in LNP (LNP-mRNA) filled in a colorless glass vial. A solution containing the equivalent of 60 μg of MAFB-7256a in a drug solution of 0.6 mL in a vial (labeled volume: 1.5 mL).

4) Lot number: Lot number is provided in the "Pharmacy Manual."

For details and the handling of the investigational product, refer to the "Investigator's Brochure (IB)" and "Pharmacy Manual."

Comparator

Comirnaty RTU IM

1) Brand name: COMIRNATY RTU intramuscular injection
2) Generic name: Coronavirus (SARS-CoV-2) RNA vaccine
3) Content and dosage form: A frozen aqueous solution containing 0.225 mg of raxtozinameran encapsulated in LNP-mRNA filled in a colorless glass vial (labeled volume 2.25 mL).
4) Lot number: Lot number is provided in the "Pharmacy Manual."

For details and the handling of comparator, refer to the "Pharmacy Manual."

5.2.2 Labeling and Packaging

Details on the packaging and labeling of the study drug are described in the "Pharmacy Manual."

5.2.3 Preparation

Details of the study drug preparation method are explained in the "Study Drug Preparation and Administration Procedures." Since this study is conducted with an observer-blind approach, the study drug preparer and study drug administration personnel must not disclose information that can identify the study drug to the blinded study personnel until unblinding.

5.2.4 Administration

The study drug will be administered to subjects according to the procedure described below. In principle, the study drug should be administered on the non-dominant arm.

1) The unblinded personnel at the study site (study drug manager or study drug management assistant designated by the study drug manager, study drug preparer, study drug administration personnel) will confirm and dispense the assigned study drug based on the IRT notification.
2) The study drug preparer will prepare the study drug in a separate location from the

blinded personnel.

3) The study drug administration personnel will administer the study drug to subjects in a manner that the blinded personnel cannot identify the administered study drug.

5.2.5 Storage

Investigational product

- DS-5670d

At 2°C to 8°C, shielded from light

Comparator

- Comirnaty RTU IM

−90°C to −60°C

Refrigerated: At 2°C to 8°C for 10 weeks*

*: Thawed vials should not be refrozen, and should be used within the shelf life.

5.3 Drugs Used in the Study Other Investigational Drugs

Not applicable.

5.4 Drug Accountability

5.4.1 Study Drugs Supplied by the Sponsor

The study drug should be managed in accordance with the "Pharmacy Manual."

The sponsor will supply the investigational product to the study site after execution of the study contract. The Ministry of Health, Labour and Welfare will supply Comirnaty RTU IM to the study drug storage depot designated by the sponsor. The sponsor will supply Comirnaty RTU IM to the study site after execution of the study contract. The study drug manager or the study drug management assistant designated by the study drug manager will receive the investigational product and Comirnaty RTU IM, and check the amount and condition of the received drugs. The sponsor should be contacted as soon as possible if there is a problem during the shipment. A study drug accountability list will be prepared for the investigational product and comparator, and must be kept current.

At the end of the study or as directed by the sponsor, unused study drugs will be returned to the sponsor. Study drugs will be returned after the monitor has completed confirmation and reconciliation of the amount of remaining unused study drugs based on the study drug accountability list. At the end of the study, the investigator or the study drug manager will prepare a copy of the final study drug accountability list and submit it to the sponsor.

5.4.2 Study Drugs Not Supplied by the Sponsor

Not applicable.

5.5 Temporary Dose Discontinuation and Reduction

If any significant abnormality is observed in the subject during study drug administration, the administration should be immediately discontinued.

5.6 Method of Confirming Administration Compliance

The investigator or subinvestigator will perform medical examinations and measure axillary body temperature of the subject before study drug administration to determine the subject's eligibility for study drug administration. If the subject has a fever of ≥ 37.5°C or suffers from a serious acute disease, study drug should be discontinued. The investigator or subinvestigator will record the date of determination of the subject's eligibility for study drug administration, the actual value of body temperature measured for eligibility evaluation or confirmation of administration compliance, and the result of the determination (acceptable or unacceptable) in the eCRF.

5.7 Prior Treatments and Concomitant Therapies

If any concomitant medication is used during the period from study drug administration to the study visit at 4 weeks after study drug administration, the investigator or subinvestigator will record the drug name, dosage regimen, route, start date, end date, and reason for use in the eCRF. If any concomitant therapy is used during the period from study drug administration to the study visit at 4 weeks after study drug administration, the investigator or subinvestigator will record the therapy name, frequency, start date, end date, and reason for the therapy in the eCRF.

5.7.1 Prohibited Concomitant Medication/Therapy

The use of the following concomitant medications and therapies will be prohibited for 4 weeks after study drug administration. However, this does not apply when the investigator or subinvestigator determines that treatment is necessary for AE that occurs after obtaining informed consent.

1) Blood transfusions (including component transfusions)
2) Gamma globulin preparations
3) Systemic administration of immunosuppressive drugs (including treatments to autoimmune diseases)
4) Immunosuppressive therapy (including radiotherapy)
5) Hematopoietic agents (except for iron and vitamin preparations)
6) Systemic corticosteroids (excluding external agents and inhalants)
7) Preventive antipyretic analgesics*
8) Other vaccines (excluding influenza vaccines)

9) Other drugs used in the study

*: The use of these drugs for prevention of fever and pain will be prohibited.

<Rationale>

1) to 6) These were included because these drugs/therapies may influence the immunogenicity evaluation of the study drug.

7) This was included because these drugs may influence the safety evaluation of the study drug.

8) This was included because these vaccines may influence the immunogenicity and safety evaluation of the study drug.

9) This was included because the safety of other drugs used in the study under development has not been established, and concomitant use of the study drug with such drugs may cause safety problems.

In addition, the use of SARS-CoV-2 vaccines (including drugs used in the study), except in this study, will be prohibited for 26 weeks after study drug administration.

<Rationale>

The use of SARS-CoV-2 vaccines (including investigational drugs), except in this study, will be prohibited for 26 weeks after study drug administration because they may influence the evaluation of long-term efficacy and safety.

5.7.2 Other Restrictions

During the study, the subject and partner should practice contraception with effective contraceptive methods (male contraceptive condom, oral contraceptive pill, intrauterine device, intrauterine hormone release system, bilateral tubal ligation, and vasectomy).

<Rationale>

This was included in consideration of the influence on the subject, subject's partner, fetuses, and newborn.

5.8 Subject Withdrawal/Discontinuation

When the subject meets the criteria described in Section 5.8.1, the investigator or subinvestigator will determine whether or not the subject should be withdrawn from the study, and if the subject is withdrawn from the study, take appropriate actions for the subject. If the subject is withdrawn from the study, the subject will not be replaced. The subject and legal representative can request

withdrawal from the study at any time even after providing informed consent.

5.8.1 Reasons for Withdrawal

- Withdrawal requested or consent withdrawn by the subject or legal representative
- AEs due to which continuing the study is considered undesirable by the investigator or subinvestigator
- Inclusion criteria not met or exclusion criteria met
- Significant deviation from the protocol
- Failure to make a visit
- Death
- Study terminated by the sponsor
- Pregnancy (subject herself)
- Otherwise considered unsuitable by the investigator or subinvestigator

5.8.2 Withdrawal Procedures

If the subject is withdrawn from the study, the investigator or subinvestigator will complete the observations and examinations described in "STUDY PROCEDURES" to the extent possible, and record the results of observations up to the date of permanent discontinuation, including the date of the last dose, and the reason for withdrawal in the eCRF.

In the case of withdrawal due to an AE, the investigator or subinvestigator should follow the subject until the AE recovers or resolves to the condition before the occurrence of the AE and disappears, AE is recovering or resolving, or AE is stabilized.

5.8.3 Subject Replacement

Not applicable.

5.8.4 Subject Re-screening Procedures

If re-consent is obtained in writing from the subject or legal representative and eligibility is confirmed, the investigator or subinvestigator may re-enroll the subject who dropped out of the study for being determined ineligible at the time of eligibility evaluation after providing informed consent or who was withdrawn from the study during the period after randomization until study drug administration. To re-enroll such a subject, the investigator or subinvestigator will record the subject number assigned at the time of dropping out or withdrawal in the eCRF of the re-enrolled subject according to the procedures specified in Section 5.1.2.

5.9 Study Completion for Individual Subjects

If the subject completes the study at 26 weeks after study drug administration, the investigator or

subinvestigator will complete the observations and examinations described in "STUDY PROCEDURES," and record the results of observations up to 26 weeks after study drug administration in the eCRF. If an AE persists after 26 weeks after study drug administration, the investigator or subinvestigator should follow the subject until the AE recovers or resolves to the condition before the occurrence of the AE and disappears, AE is recovering or resolving, or AE is stabilized. The outcome of the AE should be confirmed and recorded in the eCRF. Upon completion of these procedures, the study for the subject will be completed.

6. STUDY PROCEDURES

6.1 Informed Consent, Eligibility Evaluation, and Enrollment

1) The investigator, subinvestigator, or study staff will explain an outline of this study to the subject and legal representative and obtain written consent from the subject and, if necessary, legal representative. If the subject is aged 12 to 17 years, written consent to study participation should be obtained from the subject, depending on his/her ability of understanding. If the subject cannot sign the informed consent form (ICF) or the subject's assent is obtained orally rather than in writing, the ICF signed by the legal representative should indicate that the subject's assent has been obtained.
2) The investigator or subinvestigator will collect the following baseline characteristics and document them in the eCRF:
   - Birth year (western calendar) and the age at informed consent
   - Sex
   - Race, ethnicity
   - Height, body weight
   - Medical history/complications, name of diseases, and date of recovery (confirmed)
   - Self-reported SARS-CoV-2 infection history, date of onset or test, evaluation method (eg, RT-PCR, other nucleic acid detection methods, SARS-CoV-2 antigen test)
   - Presence or absence of a history of vaccination with SARS-CoV-2 vaccine, the most recent type of SARS-CoV-2 vaccines administered as well as dates of the administration

   Definition of a history of SARS-CoV-2 infection reported by the subject or legal representative (self-reported SARS-CoV-2 infection history)

   A subject with a history of SARS-CoV-2 infection reported by the subject or legal representative is defined as having tested positive for SARS-CoV-2 infection (based on RT-PCR, other nucleic acid detection methods, or SARS-CoV-2 antigen test) in the past or having been diagnosed with COVID-19 based on a physician's medical examination in the past. Otherwise, the subject is defined as having no self-reported SARS-CoV-2 infection history.

3) The investigator, subinvestigator, or study staff will register the required subject information in the IRT based on the source documents for the subject who provided written informed consent and obtain the subject number.
4) The investigator or subinvestigator will prepare a subject screening list based on the subject number allocated by the IRT.
5) The investigator or subinvestigator will perform the following investigations,

observations, and examinations, and confirm the subject's eligibility (based on the inclusion/exclusion criteria).

- Axillary body temperature (actual measured value)
- SARS-CoV-2 antigen test
- SARS-CoV-2 antibody test*1 (measurement using a rapid test kit at the study site)
- Pregnancy test (urine)*2

*1: If performed by venous blood, the sampling for clinical laboratory tests, immunogenicity evaluation and SARS-CoV-2 antibody test prescribed in Section 6.2 can be performed at the same time, taking into account the burden on the subject caused by blood sampling.

*2: To be performed for women of childbearing potential only.

6) The investigator, subinvestigator or study staff will enter confirmation of the subject eligibility (based on the inclusion/exclusion criteria) in the IRT. If the subject dropped out when eligibility is evaluated, the date and the reason for dropout will be entered in the IRT.

Definition of History of SARS-CoV-2 Infection at Enrollment

A subject will be defined as having a history of SARS-CoV-2 infection at enrollment if he/she is determined to have a self-reported SARS-CoV-2 infection history or have a positive SARS-CoV-2 antibody with the rapid test kit at the time of eligibility evaluation. Otherwise, the subject will be defined as having no history of SARS-CoV-2 infection.

7) The IRT will randomize the eligible subject to an administration and notification of the assigned study drug will be sent to the unblinded personnel at the study site. The investigator, subinvestigator, or study staff will enter the randomization date in the eCRF.

6.2 Study Drug Administration (Day 1, No Allowance)

1) The investigator or subinvestigator will perform the following investigations, observations, and examinations, and document them in the eCRF. The result of determination of the subject's eligibility for study drug administration will be sent to unblinded personnel at the study site (study drug preparer and study drug administration personnel).

- Clinical laboratory tests (blood/urine)
- Blood sampling for immunogenicity evaluation
- SARS-CoV-2 antibody test (to be performed at the central laboratory)
- Determination of the subject's eligibility for study drug administration specified in Section 5.6

2) The unblinded personnel at the study site (study drug manager or study drug management assistant designated by the study drug manager) will confirm the assigned study drug based on the IRT notification. The assigned study drug will be dispensed in a separate location from the blinded personnel. The study drug preparer will prepare the injection syringe with the prescribed dose of the study drug.
3) The study drug administration personnel will administer the prepared study drug to the subject according to the study drug administration procedures and record the following items in the source document: the date of study drug administration, whether or not administration was completed, the administration site (right or left arm), and whether or not any dosing error occurred. If there was a dosing error, the subject number for which the study drug was actually administered will be identified and recorded. The investigator, subinvestigator, or study staff will enter the information recorded in the source document in the eCRF. The investigator or subinvestigator will monitor the subject's condition carefully for at least 30 minutes after study drug administration, and enter the results of the monitoring in the eCRF.
4) For the initial treatment in the event of an allergic reaction, the investigator, subinvestigator, or study staff will prepare the necessary drugs and equipment for emergency treatment. The investigator or subinvestigator will enter the treatments provided against all AEs in the eCRF.
5) The investigator, subinvestigator, or study staff will give a thermometer and the electronic diary (with instruction leaflets) to the subject or legal representative, and explain how to use them. An electronic diary entry device will also be given to them, if necessary.

6.3 Telephone Contact after Study Drug Administration (Day 8, Day 15, Day 22; Allowance ± 2 Days)

1) The investigator, subinvestigator, or study staff will contact the subject or legal representative by telephone on Day 8, Day 15, and Day 22, and ask about the subject's health status. Records of telephone contact documenting whether or not the contact was made and the date of the contact will be kept. It is also acceptable to ask the subject to return to the study site for health status confirmation.
2) Based on the record of telephone contact, the investigator, subinvestigator, or study staff will record whether or not the contact was made and the date of the contact in the eCRF.
3) The investigator or subinvestigator will judge if the subject's visit is necessary based on the information collected via telephone contact, and document the judgment in the source document.

6.4 Post-dose Evaluation (Day 29, Allowance + 3 Days)

1) The subject will be requested to visit the study site for the post-dose evaluation at 4 weeks after study drug administration. The investigator or subinvestigator will confirm the subject's health status by medical interview and review the descriptions in the electronic diary to determine the seriousness, severity, and causality of nonserious unsolicited AEs, SAEs, and AEs leading to withdrawal from the study during the period from study drug administration to the study visit for the post-dose evaluation at 4 weeks after study drug administration in accordance with the definitions in Section 9.3, and record the results on the AE page of the eCRF. The use of concomitant medications/therapies during the period from study drug administration to the study visit for the post-dose evaluation at 4 weeks after study drug administration will also be confirmed.
2) The investigator or subinvestigator will perform the following investigations, observations, and examinations, and document them in the eCRF.
   - Blood sampling for immunogenicity evaluation
   - Clinical laboratory tests (blood/urine)
   - Pregnancy test (urine)*
   - SARS-CoV-2 antibody test (to be performed at the central laboratory)

   *: For women of childbearing potential only.
3) The investigator or subinvestigator will confirm that all data in the electronic diary have been transferred and then confirm that the application for data entry has been uninstalled from the electronic diary entry device. If a device has been lent to the subject or legal representative, the investigator or subinvestigator will collect the device.

6.5 Follow-up Examination (Day 57 [Allowance ± 7 Days], Day 85 [Allowance ± 7 Days], Day 113 [Allowance ± 7 Days], Day 141 [Allowance ± 7 Days], and Day 183 [Allowance ± 14 Days])

The subject will visit the study site on Day 57, Day 85, Day 113, Day 141, and Day 183. The investigator or subinvestigator will perform the following investigations, observations, and examinations, and document them in the eCRF. For subjects aged 12 to 17 years, only subjects who are able to visit the study site will visit the study site on Days 57, 113, and 141. In addition, if a solicited AE is ongoing at the study visit for the post-dose evaluation, the investigator or subinvestigator will confirm the outcome of the solicited AE, and enter the log number, event term and date of outcome of the event in the electronic diary in the eCRF.

- Blood sampling for immunogenicity evaluation
- SARS-CoV-2 antibody test (to be performed at the central laboratory)

6.6 Withdrawal/Discontinuation

If the investigator or subinvestigator judges that the subject should be withdrawn from the study, the investigator or subinvestigator will perform an interview about AEs and SAEs and an investigation on development of COVID-19, and document them in the eCRF. The following investigations, observations, and examinations will be also performed to the extent possible, and documented in the eCRF. The investigator or subinvestigator should sufficiently explain the need for examinations and observations to the subject and legal representative, and document the subject's informed consent in the source document:

- Blood sampling for immunogenicity evaluation
- Clinical laboratory tests (blood/urine)*1
- Pregnancy test (urine)*2
- SARS-CoV-2 antibody test (to be performed at the central laboratory)

*1: To be performed only for subjects withdrawn from the study during the observation period.

*2: To be performed only for women of childbearing potential withdrawn from the study during the observation period.

If the subject is withdrawn from the study during the observation period, the investigator or subinvestigator will confirm that all data in the electronic diary have been transferred and then confirm that the application for data entry has been uninstalled from the electronic diary entry device. If a device has been lent to the subject or legal representative, the investigator or subinvestigator will collect the device.

6.7 Blood Sampling for Immunogenicity Evaluation

The investigator or subinvestigator will collect samples for immunogenicity evaluation before study drug administration (Day 1) and at 4 weeks (Day 29), 8 weeks (Day 57), 12 weeks (Day 85), 16 weeks (Day 113), 20 weeks (Day 141), and 26 weeks (Day 183) after study drug administration. However, if subjects are aged 12 to 17 years, the samples will be collected from evaluable subjects only at 8 weeks (Day 57), 16 weeks (Day 113), and 20 weeks (Day 141) after study drug administration.

Samples for immunogenicity evaluation will also be collected at the time of withdrawal from the study, if possible. The investigator or subinvestigator will record whether or not blood sampling was performed, and the date of sampling for measurement of blood neutralizing activity against SARS-CoV-2 in the eCRF. The handling, storage, and shipping of samples are specified in a separately prepared procedure manual.

Residual samples after antibody titer measurement may be used for exploratory studies on the safety information of DS-5670d or the relationship between immunogenicity and study drug administration or be used for future evaluation of analytical procedures. Details are specified in a separately prepared plan. Genome and gene analysis research will not be conducted using the

samples.

6.7.1 Sample Handling Methods

Details are specified in a separately prepared procedure manual.

6.7.2 Labeling for Sample Storage Containers and Shipment Method

Details are specified in a separately prepared procedure manual.

6.7.3 Evaluation Methods

Details are specified in a separately prepared procedure manual.

6.8 Electronic Diary

The investigator, subinvestigator, or study staff will distribute the electronic diary account to the subject or legal representative on the day of study drug administration (Day 1). The investigator, subinvestigator, or study staff will ask the subject or legal representative to record the following items in the electronic diary during the period from the day of study drug administration to the visit for the post-dose evaluation at 4 weeks after study drug administration, and regularly check the details of descriptions on the electronic data capture (EDC) system where the relevant data will be transferred and confirm entry compliance and the safety of the subject. If the subject is aged 12 to 17 years, the legal representative will make entries in the electronic diary in principle. However, if the investigator or subinvestigator judges that the subject can comply with the rules of the study and report symptoms, etc., specified in the protocol, the entry by the subject is acceptable. The investigator or subinvestigator will record the person who recorded data in the electronic diary in the eCRF.

At the visit for the post-dose evaluation at 4 weeks after study drug administration or the visit after withdrawal from the study during the observation period, the investigator or subinvestigator will confirm that all data of the electronic diary have been transferred and then confirm that the application for data entry has been uninstalled from the electronic diary entry device of the subject or legal representative. If a device has been lent to the subject or legal representative, the investigator or subinvestigator will collect the device. If a solicited AE is ongoing at the study visit for the post-dose evaluation, the investigator or subinvestigator will confirm the outcome of the solicited AE, and enter the log number, event term and date of outcome of the event in the electronic diary in the eCRF.

<<Items to be recorded on the electronic diary>>

- Axillary body temperature
  1) The subject or legal representative will measure the axillary body temperature of the subject every day for 7 days after study drug administration (Day 1 to Day 8) and record the results. If a fever of 37.5°C and higher persists even after an elapse of 7 days since

study drug administration, body temperature will be measured and recorded every day until it drops below 37.5°C.

2) On the day of the study drug administration (Day 1), the axillary body temperature will be measured once after the study drug administration, within that day. On Day 2 to Day 8, axillary body temperature will be measured once a day. If the body temperature is measured at multiple times a day, the maximal body temperature will be recorded. The temperature should not be taken immediately after eating, exercising, or taking a bath.

- Solicited Injection Site Adverse Events

1) The subject or legal representative will monitor the injection site of the subject every day for 7 days after study drug administration (Day 1 to Day 8). In the electronic diary, the presence or absence of injection site reactions (redness, swelling, induration, pain, warmth, and pruritus) will be monitored and, if present, their severity (see Section 9.3) will be recorded. If redness, swelling, or induration is observed, the long diameter of it will be recorded. If such events persist even after an elapse of 7 days since study drug administration, the monitoring of severity (long diameter) will be continued every day until the symptoms are resolved, and the result will be recorded.
2) On the day of the study drug administration (Day 1), the injection site will be monitored once after the study drug administration, within that day. On Day 2 to Day 8, the injection site will be monitored once a day.
3) If there are any new symptoms of injection site AEs before the visit for the post-dose evaluation at 4 weeks after study drug administration, except for the above period, the onset of the symptoms will be recorded as unsolicited AEs in the electronic diary. The symptoms, date of onset, severity (see Section 9.3), and the date of disappearance will be reported to the personnel during a medical interview or telephone contact.

- Solicited Systemic Adverse Events

1) The subject or legal representative will monitor the presence or absence of solicited systemic AEs (fever, malaise, headache, rash, and myalgia) of the subject every day for 7 days after study drug administration (Day 1 to Day 8) and, if there are any symptoms, record the symptoms, date of onset, and severity (see Section 9.3). If such events persist even after an elapse of 7 days since study drug administration, the symptoms will be monitored every day until they resolve, and the result will be recorded.
2) On the day of the study drug administration (Day 1), the presence or absence of systemic AEs will be monitored after the study drug administration, within that day. From Day 2 to Day 8, the presence or absence of systemic AEs will be monitored once a day.
3) If there are any new symptoms of systemic AEs before the visit for the post-dose

evaluation at 4 weeks after study drug administration, except for the above period, the onset of the symptoms will be recorded as unsolicited AEs in the electronic diary. The symptoms, date of onset, severity (see Section 9.3), and the date of resolution will be reported to the personnel during a medical interview or telephone contact.

- Unsolicited AEs
  1) The subject or legal representative will monitor the presence or absence of unsolicited AEs of the subject every day for 28 days after study drug administration (Day 1 to Day 29) and, if there are any symptoms, record the onset of the symptoms.

6.9 Clinical Laboratory Tests (Blood/Urine)

The investigator or subinvestigator will collect samples for blood tests and urinalysis before study drug administration (Day 1) and at the post-dose evaluation at 4 weeks after study drug administration (Day 29), and record whether or not the samples were collected and the date of blood and urine sampling in the eCRF. Blood and urine sampling will also be performed at withdrawal from the study during the observation period, if possible, and whether or not the samples were collected and the date of blood and urine sampling will be recorded in the eCRF. Detailed methods for sampling, collection, and testing procedures are specified in a separately prepared procedure manual.

6.10 SARS-CoV-2 Antigen Test

The investigator or subinvestigator will collect nasopharyngeal swab samples from the subject at the time of eligibility evaluation before study drug administration (Day 1) and perform the SARS-CoV-2 antigen test. The measurement will be performed at the study site.

The investigator or subinvestigator will record whether or not the test was performed, the date of the test, and the "negative" or "positive" result in the eCRF.

6.11 SARS-CoV-2 Antibody Test

The investigator or subinvestigator will perform the SARS-CoV-2 antibody test using the rapid test kit at the time of eligibility evaluation before study drug administration (Day 1). The "negative" or "positive" result will be determined based only on anti-N protein antibody. The measurement will be performed at the study site.

The investigator or subinvestigator will record whether or not the test was performed, the date of the test, and the "negative" or "positive" result in the eCRF.

The investigator or subinvestigator will collect blood samples for the SARS-CoV-2 antibody test before study drug administration (Day 1) and at the post-dose evaluation at 4 weeks (Day 29), and at 8 weeks (Day 57), 12 weeks (Day 85), 16 weeks (Day 113), 20 weeks (Day 141), and 26 weeks (Day 183) after study drug administration. However, if subjects are aged 12 to 17 years, the samples will be

collected from evaluable subjects only at 8 weeks (Day 57), 16 weeks (Day 113), and 20 weeks (Day 141) after study drug administration.

Blood will also be collected from subjects at withdrawal from the study, if possible, for the SARS-CoV-2 antibody test. The measurement will be performed at the central laboratory. Detailed methods for sampling, collection, and testing procedures are specified in a separately prepared procedure manual.

The investigator or subinvestigator will record whether or not the test was performed and the date of the test in the eCRF.

6.12 Pregnancy Test

The investigator or subinvestigator will perform pregnancy tests using urinary samples for female subjects of childbearing potential at the time of eligibility evaluation before study drug administration (Day 1), and at the post-dose evaluation at 4 weeks after study drug administration (Day 29). Pregnancy tests will also be performed using urinary samples at withdrawal from the study during observation period, if possible. The measurement will be performed at the study site.

The investigator or subinvestigator will determine whether they are pregnant and record whether or not the test was performed, the date performed and results in the eCRF.

6.13 Response in the Case of Suspected SARS-CoV-2 Infection

The investigator, subinvestigator, or study staff will explain to the subject and legal representative about symptoms suspected to indicate SARS-CoV-2 infection and instruct the legal representative to immediately contact the study site if the subject encounters such symptoms within 26 weeks (Day 183) after study drug administration or if the legal presentative voluntarily determines that the subject may have been infected and the subject visits a medical institution providing COVID-19 treatment such as a fever outpatient clinic.

If the investigator or subinvestigator determines that the subject is suspected to have been infected with SARS-CoV-2 based on symptoms and the subject's status heard from the subject or legal representative via telephone, etc. (eg, the subject's cohabiting family member is found to have been infected with SARS-CoV-2 in the last few days, or a person involved in the environment around the subject such as school is found to have been infected with SARS-CoV-2), the subject or legal representative should be instructed to ensure that the subject will promptly visit the study site or a medical institution providing COVID-19 treatment such as a fever outpatient clinic. The investigator or subinvestigator will record the axillary body temperature measurement (actual measured value), the presence or absence of respiratory symptoms, malaise, headache, gastrointestinal symptoms, nasal discharge/nasal congestion, pharyngeal pain, dysgeusia, olfactory dysfunction, arthralgia, or myalgia, as well as the date of onset of symptoms, as obtained via telephone, etc., in the eCRF.

In the case of receiving treatment at the study site, nasopharyngeal swab samples for RT-PCR

testing will be collected to determine the presence or absence of SARS-CoV-2 infection. Detailed methods for sampling, collection, and testing procedures are specified in a separately prepared procedure manual. Leftover nasopharyngeal swab samples after RT-PCR testing may be used for future examinations. Details are specified in a separately prepared plan.

Blood sampling for immunogenicity evaluation will be performed whenever possible and the samples will be cryopreserved at ≤ −70°C after serum separation.

If the subject visits a medical institution providing COVID-19 treatment such as a fever outpatient clinic, the results of RT-PCR testing and diagnosis will be obtained from the medical institution.

The investigator or subinvestigator will record the date of the subject's outpatient visit, whether or not blood sampling for immunogenicity evaluation was performed, the date of the sampling, the results of RT-PCR, other tests (eg, nucleic acid detection), or SARS-CoV-2 antigen test, and the date and result of the diagnosis in the eCRF.

<Definition of symptoms of suspected SARS-CoV-2 infection>

A fever of ≥ 37.5°C, with at least one of the following symptoms:

- Cough, shortness of breath/difficulty breathing, fatigue/malaise, headache, gastrointestinal symptoms, nasal discharge/nasal congestion, pharyngalgia, new dysgeusia/olfactory dysfunction, arthralgia, myalgia/generalized pain

6.14 Response in the Case Where Cohabiting Family of Subject Is Determined to Be Infected with SARS-CoV-2

The investigator, subinvestigator, or study staff should instruct the subject or legal representative to immediately contact the study site if the subject's cohabiting family member has been diagnosed with SARS-CoV-2, for 26 weeks (Day 183) after study drug administration.

If the investigator, subinvestigator, or study staff obtains information that the subject's cohabiting family member has been diagnosed with SARS-CoV-2, the presence or absence of occurrence of symptoms suspected to indicate SARS-CoV-2 infection should be confirmed by telephone or other means. The investigator or subinvestigator will record the date that the subject's cohabiting family member was determined to have been infected with SARS-CoV-2 in the eCRF. If the subject is confirmed to have experienced symptoms suspected to indicate SARS-CoV-2 infection by telephone, etc., this should be handled in accordance with Section 6.13.

The investigator, subinvestigator, or study staff should instruct the subject or legal representative to immediately contact the study site if the subject has symptoms suspected to indicate SARS-CoV-2 infection after the date that the subject's cohabiting family member is judged to have been infected with SARS-CoV-2.

7. EFFICACY EVALUATIONS

7.1 Method for Evaluation of Efficacy Endpoints

7.1.1 Primary Endpoint

- GMT of blood neutralizing activity against SARS-CoV-2 (Omicron strain XBB.1.5.6) at 4 weeks after study drug administration in adults and children aged 12 years and older with at least either a history of SARS-CoV-2 infection or vaccination with SARS-CoV-2 vaccine
- Seroresponse rate of blood neutralizing activity against SARS-CoV-2 (Omicron strain XBB.1.5.6) at 4 weeks after study drug administration in adults and children aged 12 years and older with at least either a history of SARS-CoV-2 infection or vaccination with SARS-CoV-2 vaccine

7.1.1.1 Definition of Seroresponse Rate

Percentage of subjects with an increase of ≥ 4 times in the blood neutralizing activity against SARS-CoV-2 after study drug administration, compared with the blood neutralizing activity against SARS-CoV-2 before study drug administration.

7.1.2 Secondary Endpoints

7.1.2.1 Key Secondary Endpoints

- GMT of blood neutralizing activity against SARS-CoV-2 (Omicron strain XBB.1.5.6) at 4 weeks after study drug administration in adults and children aged 12 years and older with or without a history of SARS-CoV-2 infection or vaccination with SARS-CoV-2 vaccine
- Seroresponse rate of blood neutralizing activity against SARS-CoV-2 (Omicron strain XBB.1.5.6) at 4 weeks after study drug administration in adults and children aged 12 years and older with or without a history of SARS-CoV-2 infection or vaccination with SARS-CoV-2 vaccine

7.1.2.2 Other Secondary Endpoints

- Incidence of COVID-19 for 26 weeks after study drug administration

7.1.2.2.1 Definition of Onset of COVID-19

The onset of symptoms of COVID-19 is defined as the presence of at least one of the following symptoms and a positive test result of SARS-CoV-2 infection (eg, RT-PCR, SARS-CoV-2 antigen test): fever with a body temperature of ≥ 37.5°C, cough, shortness of breath/difficulty breathing, fatigue/malaise, headache, gastrointestinal symptoms, nasal discharge/nasal congestion, pharyngalgia, new dysgeusia/olfactory dysfunction, arthralgia, myalgia/generalized pain.

7.1.3 Exploratory Endpoints

- GMT of blood neutralizing activity against SARS-CoV-2 (Omicron strain XBB.1.5.6) and seroresponse rate at 8 weeks, 12 weeks, 16 weeks, 20 weeks, and 26 weeks after study drug administration
- GMT of blood neutralizing activity* against SARS-CoV-2 and seroresponse rate at 4 weeks, 8 weeks, 12 weeks, 16 weeks, 20 weeks, and 26 weeks after study drug administration

*: Details of variants to be evaluated are specified in a separately prepared plan.

7.2 Appropriateness of Selected Efficacy Endpoints

The primary endpoint of the study is the GMT of blood neutralizing activity against SARS-CoV-2 (Omicron strain XBB.1.5.6) and seroresponse rate at 4 weeks after study drug administration.

In conjunction with ongoing SARS-CoV-2 vaccination with existing vaccines, the conduct of placebo-controlled clinical studies of subsequent SARS-CoV-2 vaccines has become difficult, and there is ongoing global debate on alternative studies. The ICMRA, which is comprised of top pharmaceutical authorities, has discussed the clinical study methods for subsequent development of SARS-CoV-2 vaccines and has determined that there is no objection to proceeding with the development by alternatively using a randomized, active-comparator, non-inferiority or superiority study with an indicator of immunogenicity as an endpoint.[27]

Alternative endpoints for the disease-preventive effect of the SARS-CoV-2 vaccines should be considered based on a scientific understanding of the mechanism of the disease-preventive effect of the SARS-CoV-2 vaccines. Khoury et al. reported that a correlation between blood neutralizing activity against SARS-CoV-2 after vaccination with approved SARS-CoV-2 vaccines and the disease-preventive effect on SARS-CoV-2 has been shown, and it has been proposed that immunogenicity evaluation can be a surrogate marker for the disease-preventive effect.[28]

In the Notice of the PMDA entitled "Principles for the Evaluation of Vaccines Against the Novel Coronavirus SARS-CoV-2, (Appendix 4) Immunogenicity-based evaluation of variant vaccines modified from parent vaccines and booster vaccines with new active ingredients,"[31] it is stated that "the non-inferiority of the novel booster vaccine candidate to the active comparator vaccine should be tested using the GMT of neutralizing activity against the target SARS-CoV-2 strain and the immune response rate as co-primary endpoints."

Based on the above, it was decided to verify the non-inferiority of DS-5670d to Comirnaty RTU IM using blood neutralizing activity against SARS-CoV-2 (Omicron strain XBB.1.5.6) as an indicator in this study.

8. PHARMACOKINETIC/PHARMACODYNAMIC EVALUATIONS

8.1 Pharmacokinetic Evaluations

Not applicable.

8.2 Pharmacodynamic Evaluations

Not applicable.

8.3 Biomarker Evaluations

Not applicable.

8.4 Immunogenicity

Not applicable.

8.5 Pharmacogenetic Analysis

Not applicable.

9. SAFETY EVALUATION AND REPORTING

9.1 Method for Evaluation of Safety Endpoints

Data will be collected and evaluated as specified in Section 6.8 (Electronic Diary), Section 9.2 (Adverse Event Collection and Reporting), Section 9.9 (Clinical Laboratory Evaluations), and Section 9.10 (Vital Signs).

9.2 Adverse Event Collection and Reporting

All SAEs (for the definition of SAEs, see 9.3.1 and 9.3.2) reported after the subject or legal representative signs the ICF until the study visit at 26 weeks after study drug administration (follow-up period), regardless of whether they are confirmed by the investigator or subinvestigator or reported by the subject or legal representative, will be recorded on the AE page of the eCRF. SAEs will be followed until the AE disappears, is stabilized, or is found not to be serious, or the subject is lost to follow-up.

Nonserious unsolicited AEs reported from study drug administration to the study visit at 4 weeks after study drug administration will be recorded on the AE page of the eCRF. Medical conditions that were diagnosed or known to exist before the first administration of the study drug (including clinically significant laboratory values/vital signs that are out of normal range but are not related to the underlying disease) will be recorded not on the AE page but as part of medical history or complications. For any medical occurrence ongoing on the date of the first administration of study drug, the eCRF "Medical History/Complications" module will record the start date of the medical occurrence even if the onset date is before the first administration of study drug. Worsening of preexisting medical occurrences or symptoms (excluding conditions related to the underlying disease), including worsening of severity after the first administration of the study drug, will be recorded on the AE page. SAEs will be reported according to the procedures specified in Section 9.6.

All clinical laboratory test results and vital signs will be evaluated and their clinical significance will be determined by the investigator or subinvestigator. Sporadic abnormalities of clinical laboratory test results and vital signs (ie, findings not consistent with part of reported diagnoses) will be reported as AEs if they are symptomatic, lead to permanent discontinuation of study treatment, require treatment for symptomatic relief, and are regarded as AEs based on the clinical judgment of the investigator or subinvestigator.

The investigator or subinvestigator will confirm whether any AEs have occurred through medical interviews at study visits, subject's electronic diary, hearing of the subject's condition from the subject by telephone confirmation, etc., during the study, or medical examinations. AEs may be directly observed, reported spontaneously by the subject or legal representative, or found by medical interview at each study visit. The subject and legal representative should be questioned in a general way, without asking about the occurrence of any specific symptoms. The investigator or subinvestigator will evaluate nonserious unsolicited AEs, SAEs, and AEs leading to permanent

discontinuation of study treatment or withdrawal from the study, and determine the seriousness, severity, and causality according to the definitions provided in Section 9.3. The evaluation by the investigator or subinvestigator will be clearly recorded in the source documents retained at the study site, with signature of the investigator or subinvestigator.

AEs should always be reported as a diagnosis. When a diagnosis is unavailable, the primary sign or symptom will be reported as the event term with additional details included in the narrative until the diagnosis becomes available. If the signs and symptoms are distinct and do not suggest a common diagnosis, they should be reported as individual entries of AEs.

For events that are serious due to hospitalization, the reason for hospitalization should be reported as SAE (diagnosis or symptom requiring hospitalization). Procedures (treatment and surgery) will not be handled as AEs, with no exception, but the reason for the procedure may be an AE. Pre-planned (before signing the ICF) procedures or treatments under hospitalization for preexisting medical conditions that do not worsen in severity need not be reported as SAEs (see Section 9.3.2 for definitions).

For deaths, after confirming the underlying or immediate cause of death, it should always be reported as an SAE.

Any serious, untoward event that may occur subsequently to the reporting period that the investigator or subinvestigator evaluates as related to the study drug should also be handled and reported as an SAE.

If an AE occurs, the investigator or subinvestigator will take appropriate actions, and immediately report to the sponsor according to the procedures in Section 9.6 when it falls under the definitions of SAEs. The investigator or subinvestigator will then continue follow-up until the AE recovers or resolves to the condition before the occurrence of the AE and the AE disappears or is recovering/resolving (even after the protocol-specified follow-up period), wherever possible. However, even if the AE is not confirmed to have recovered or resolved, if it is judged that the subject's condition remains stable and his/her safety can be assured, the investigator or subinvestigator will explain the matter to the subject and legal representative, and then complete the follow-up of the study (treatment of the relevant symptom will be continued).

9.3 Adverse Event

9.3.1 Definition of Adverse Event

An AE is any untoward medical occurrence in a subject administered an investigational product and that does not necessarily have to have causality with the treatment. An AE can therefore be any unfavorable and unintended sign (eg, an abnormal clinical laboratory finding), symptom, or disease temporally associated with the use of an investigational product, whether or not considered related to the investigational product.

The AEs in this study that are specified in Section 9.3.3 and Section 9.3.4 will be referred to as

"solicited AEs" and "unsolicited AEs," respectively. The investigator or subinvestigator will be responsible for determining the occurrence of nonserious unsolicited AEs, SAEs, and AEs leading to permanent discontinuation of study treatment or withdrawal from the study, including clinical laboratory evaluations. The presence or absence of nonserious solicited AEs will be determined and spontaneously reported by the legal representative using the electronic diary.

9.3.2 Serious Adverse Events

An SAE is any untoward medical occurrence after informed consent, at any dose, that is applicable to the following seriousness criteria:

- results in death;
- is life-threatening;
- requires inpatient hospitalization or prolongation of existing hospitalization;
- results in persistent or significant disability/incapacity;
- is a congenital anomaly/birth defect; or
- is an important medical event.

Note: The term "life-threatening" in the definition of "serious" refers to an event in which the subject was at risk of death at the time of the event; it does not refer to an event which hypothetically might have caused death if it were more severe.

Medical and scientific judgment should be exercised in deciding whether expedited reporting is appropriate in other situations, such as "important medical events" that may not be immediately life threatening or result in death or hospitalization but may jeopardize the subject or may require intervention to prevent one of the above five outcomes. Examples include allergic bronchospasm, convulsions, blood dyscrasias, or development of drug dependency or drug abuse.

Notes:

- Procedures are not AEs or SAEs, but the reason for the procedure may be an AE or SAE.
- Hospitalization for pre-planned (before informed consent) procedures or treatments, or preexisting medical conditions that do not worsen in severity needs not be reported as SAEs.

9.3.3 Solicited Adverse Events

Solicited AEs are defined as "solicited injection site AEs" and "solicited systemic AEs" reported spontaneously by the subject or legal representative in the electronic diary.

9.3.3.1 Solicited Injection Site Adverse Events

Solicited injection site AEs are injection site redness, swelling, induration, pain, warmth, and pruritus that occur within 7 days after study drug administration.

9.3.3.2 Solicited Systemic Adverse Events

Solicited systemic AEs are fever, malaise, headache, rash, and myalgia that occur within 7 days after study drug administration.

9.3.4 Unsolicited Adverse Events

Unsolicited AEs are defined as AEs other than "solicited AEs."

9.3.5 Severity Evaluation

The subject or legal representative will evaluate the severity of solicited systemic AEs (injection site and systemic) according to the definitions in Section 9.3.5.1 "Severity of Solicited Injection Site Adverse Events" and Section 9.3.5.2 "Severity of Solicited Systemic Adverse Events," and record the evaluation in the electronic diary.

The investigator or subinvestigator will evaluate the severity of nonserious unsolicited AEs, SAEs, and AEs leading to permanent discontinuation of study treatment or withdrawal from the study according to the definitions in Section 9.3.5.1 "Severity of Solicited Injection Site Adverse Events," Section 9.3.5.2 "Severity of Solicited Systemic Adverse Events," and Section 9.3.5.3 "Severity of Unsolicited Adverse Events."

Difference between severity and seriousness: Severity is used to describe the intensity of a specific event while the event itself may be of relatively minor medical significance (eg, severe headache). This is not the same as "seriousness," which is based on patient/event outcome at the time of the event.

9.3.5.1 Severity of Solicited Injection Site Adverse Events

The subject or legal representative will determine the severity of solicited injection site AEs (pain, warmth, and pruritus) according to the criteria in Table 9.3-1~~Table 9.3-1Table 9.3-1~~[32]. For other solicited injection site AEs (redness, swelling, and induration), the severity will be determined according to the criteria in Table 9.3-1~~Table 9.3-1Table 9.3-1~~, based on the information recorded in the electronic diary by the subject or legal representative. The investigator or subinvestigator will determine the severity of serious solicited injection site AEs and solicited injection site AEs leading to permanent discontinuation of study treatment or withdrawal from the study according to the criteria in Table 9.3-1~~Table 9.3-1Table 9.3-1~~, with reference to the information recorded in the electronic diary by the subject or legal representative.

Table 9.3-1 Severity of Solicited Injection Site Adverse Events

AE	Mild (Grade 1)	Moderate (Grade 2)	Severe (Grade 3)
Redness	The long diameter is 2.5 cm to 5 cm.	The long diameter is > 5 cm to 10 cm.	The long diameter is > 10 cm, or necrosis/exfoliative dermatitis.

AE	Mild (Grade 1)	Moderate (Grade 2)	Severe (Grade 3)
Swelling	The long diameter is 2.5 cm to 5 cm.	The long diameter is > 5 cm to 10 cm.	The long diameter is > 10 cm, prevents daily activities, or necrosis.
Induration	The long diameter is 2.5 cm to 5 cm.	The long diameter is > 5 cm to 10 cm.	The long diameter is > 10 cm, prevents daily activities, or necrosis.
Pain	Tolerable with no interference with daily activities.	Repeated use of anti-inflammatory analgesics for > 24 hours, or some interference with daily activities.	Not alleviated by the repeated use of anti-inflammatory analgesics, or prevents daily activities.
Warmth	Warm feeling, but drug treatment is not required.	Warm feeling, and drug treatment is required.	Prevents daily activities.
Pruritus	Itchy, but drug treatment is not required.	Itchy, and drug treatment is required.	Prevents daily activities.

9.3.5.2 Severity of Solicited Systemic Adverse Events

The subject or legal representative will determine the severity of solicited systemic AEs (fever, malaise, headache, rash, and myalgia) according to the criteria in Table 9.3-2~~Table 9.3-2Table 9.3-2~~ [32]. For fever, the severity will be determined according to the criteria in Table 9.3-2~~Table 9.3-2Table 9.3-2~~, based on the information in the electronic diary recorded by the subject or legal representative. The investigator or subinvestigator will determine the severity of serious solicited systemic AEs and solicited systemic AEs leading to permanent discontinuation of study treatment or withdrawal from the study according to the criteria in Table 9.3-2~~Table 9.3-2Table 9.3-2~~, with reference to the information recorded in the electronic diary by the subject or legal representative.

Table 9.3-2 Severity of Solicited Systemic Adverse Events

AE	Mild (Grade 1)	Moderate (Grade 2)	Severe (Grade 3)
Fever	37.5°C–38.4°C	38.5°C–38.9°C	39.0°C –
Malaise	Tolerable with no interference with daily activities.	Some interference with daily activities.	Significantly prevents daily activities, such as school life.
Headache	Tolerable with no interference with daily activities.	Repeated use of anti-inflammatory analgesics for > 24 hours, or some interference with daily activities.	Not alleviated by the repeated use of anti-inflammatory analgesics, or prevents daily activities.
Rash	Local (non-injection sites).	Systemic.	Systemic rash, accompanied by symptoms such as pain, blisters, and ulcers.
Myalgia	Tolerable with no interference with daily activities.	Some interference with daily activities.	Significantly prevents daily activities, such as school life.

9.3.5.3 Severity of Unsolicited Adverse Events

The investigator or subinvestigator will determine the severity of "tenderness of injection site," among the unsolicited injection site AEs confirmed by medical interview, telephone contact, etc.,

according to the criteria in Table 9.3-3Table 9.3-3Table 9.3-3[32].

Table 9.3-3 Severity of Unsolicited Adverse Events at the Injection Site (Tenderness)

AE	Mild (Grade 1)	Moderate (Grade 2)	Severe (Grade 3)
Tenderness of injection site	Mild discomfort to touch at the injection site.	Discomfort with movement of the injected arm.	Significant discomfort at rest.

The investigator or subinvestigator will determine the severity of "nausea," "vomiting," and "diarrhea," among the unsolicited systemic AEs confirmed by medical interview, telephone contact, etc., according to the criteria in Table 9.3-4Table 9.3-4Table 9.3-4[32].

Table 9.3-4 Severity of Unsolicited Systemic Adverse Events (Nausea, Vomiting, Diarrhea)

AE	Mild (Grade 1)	Moderate (Grade 2)	Severe (Grade 3)
Nausea	Tolerable with no interference with daily activities.	Some interference with daily activities.	Requires medication.
Vomiting	1 to 2 episodes per 24 hours.	≥ 3 episodes over 24 hours.	Requires outpatient intravenous drip infusion (hydration).
Diarrhea	Is tolerable with no interference with daily activities, or is associated with 2 to 3 episodes of loose stools per 24 hours.	4 to 5 episodes of loose stools per 24 hours.	≥ 6 episodes of watery stools per 24 hours, or requires outpatient intravenous drip infusion (hydration).

The investigator or subinvestigator will determine the severity of unsolicited AEs other than "tenderness of injection site," "nausea," "vomiting," and "diarrhea" confirmed by medical interview, telephone contact, etc., according to the following criteria:

- Mild: Awareness of sign or symptom, but easily tolerated, i.e., does not interfere with subject's usual function.
- Moderate: Interfering with daily activities.
- Severe: Incapacitating with inability to work or do usual activity, i.e., interferes significantly with subject's usual function.

9.3.6 Causality Evaluation

Solicited AEs (injection site and systemic), except for serious solicited AEs (injection site and systemic) and solicited AEs leading to permanent discontinuation of study treatment or withdrawal from the study, should be evaluated as "related" to the study drug.

The investigator or subinvestigator will evaluate the causality with the study drug for all nonserious unsolicited AEs, SAEs, and AEs leading to permanent discontinuation of study treatment or withdrawal from the study according to his/her own clinical judgment and the definitions below.

The causality evaluation must be made based on the available information and can be updated as new information becomes available.

- Related:
    - The event follows a reasonable temporal sequence from study drug administration, and cannot be reasonably explained by the subject's clinical state or other factors (eg, disease under study, concurrent diseases, concomitant medications other than the study drug). OR
    - The occurrence of event follows a reasonable temporal sequence from study drug administration, and is a known reaction to the drug under study or its chemical group, or is predicted by known pharmacology.
- Not related:
    - The event does not follow a reasonable temporal sequence from study drug administration or can be reasonably explained by the subject's clinical state or other factors (eg, disease under study, concurrent diseases, concomitant medications).

AEs evaluated as "related" to the study drug will be handled as adverse reactions.

9.3.7 Action Taken for the Study Drug in Response to Adverse Events

The investigator or subinvestigator will record any action taken for the study drug in response to nonserious unsolicited AEs, SAEs, and AEs leading to permanent discontinuation of study treatment or withdrawal from the study that occur after study drug administration in the eCRF, as follows:

- Dose not change: No change in study drug dosage and administration was made.
- Permanent discontinuation: The study drug was permanently discontinued.
- Temporary discontinuation: The study drug was temporarily discontinued.
- Not applicable: The subject died, study drug administration was completed before the reaction/event, or the reaction/event occurred before the start of study drug administration.

9.3.8 Other Action Taken for Adverse Events

The investigator or subinvestigator will record any other action taken in response to nonserious unsolicited AEs, SAEs, and AEs leading to permanent discontinuation of study treatment or withdrawal from the study that occur after study drug administration in the eCRF, as follows:

- None
    - No treatment was required.
- Medication required
    - Prescription and/or over-the-counter drugs were required to treat the AE.
- Hospitalization or prolongation of existing hospitalization required

    - Hospitalization was required or prolonged due to the AE, whether or not medication was required.
- Other

9.3.9 Adverse Event Outcome

The investigator or subinvestigator will record the outcome of nonserious unsolicited AEs, SAEs, and AEs leading to permanent discontinuation of study treatment or withdrawal from the study that occur after study drug administration in the eCRF, as follows:

- Recovered/resolved
    - The subject fully recovered from the AE, with no residual effect observed.
- Recovering/resolving
    - The AE has improved but has not fully resolved.
- Not recovered/not resolved
    - The AE itself is still present and observable.
- Recovered/resolved with sequelae
    - Residual effects of the AE are still observed.
    - Sequelae/residual effects are present.
- Fatal
    - "Fatal" should be used when death is a direct outcome of the AE.
- Unknown

9.4 Adverse Events of Special Interest

The AEs of special interest are defined as "solicited injection site AE," "solicited systemic AEs," "combined elevations of aminotransferases and bilirubin," and "ADE."

9.4.1 Combined Elevations of Aminotransferases and Bilirubin

Combined elevations of aminotransferases and bilirubin, either serious or nonserious and whether or not causally related, as a potential case of Hy's Law (alanine aminotransferase [ALT] or aspartate aminotransferase [AST] ≥ 3 × upper limit of normal [ULN] and blood bilirubin ≥ 2 × ULN, with no increase in alkaline phosphatase (ALP), in the absence of other factors for the increases in both aminotransferases and total bilirubin), even when evaluated to be due to disease progression by the investigator or subinvestigator, should always be reported to the sponsor using the eCRF, with the investigator's or subinvestigator's evaluation of seriousness, causality, and a detailed narrative.[33] This event should be reported within 24 hours of the investigator's or subinvestigator's awareness of the event.

If study drug administration is permanently discontinued due to liver enzyme abnormalities, the subject will be followed as described in Section 5.8 until the AE recovers or resolves to the condition

before the occurrence of the AE and disappears, AE is recovering or resolving, or AE is stabilized in order to determine the nature and severity of the potential liver injury.

9.4.2 Antibody Dependent Enhancement

If exacerbation of respiratory symptoms is suspected, such as pneumonia, after study drug administration, tests necessary for diagnosing SARS-CoV-2 infection (eg, RT-PCR, antigen test) will be performed wherever possible to determine whether it is caused by ADE due to SARS-CoV-2 vaccination. If the exacerbation is diagnosed as ADE due to study drug administration, it should be recorded as an AE in the eCRF. In addition, to examine the relation with ADE, blood sampling will be performed as promptly as possible, to cryopreserve serum.

9.5 Investigational Device or Investigational Combination Product Defects/Regenerative Medicine Products Defects

Not applicable.

9.6 Reporting of Serious Adverse Events: Procedures for the Investigator or Subinvestigator

The types of events listed below should be reported by the investigator or subinvestigator to the sponsor using the EDC, promptly after becoming aware of relevant information (within 24 hours of awareness).

The investigator should also report the detail of the event in writing promptly to the sponsor and the head of the study site. The written report to the head of the study site should be made in accordance with the procedures and format specified by the study site.

- SAEs (See Section 9.3.2 for definitions.)
- Combined elevations of aminotransferases and bilirubin, either serious or nonserious, as a potential case of Hy's Law (See Section 9.4.1 for details.)

All events (serious and nonserious) that fall under either of the above categories should be reported with the investigator's or subinvestigator's evaluation of the seriousness, severity, and causality with the study drug. A detailed narrative that summarizes the course of the event, including its evaluation, treatment, and outcome, should also be submitted. Specific or estimated dates of event onset, treatment, and resolution should be included when available. Medical history, concomitant medications, and clinical laboratory data that are relevant to the event should also be summarized in the narrative. For fatal events, the narrative should state whether an autopsy was or will be performed, and include the results if available. Source documents (including medical reports) will be retained at the study site and need not be submitted to the sponsor for the purpose of SAE reporting.

Upon receipt of urgent safety queries from the sponsor, the investigator or subinvestigator must follow and address them promptly. Follow-up information and responses to non-urgent safety queries

should be combined for reporting to provide the most complete data possible in the eCRF.

In situations where EDC is not available, the paper serious adverse event report (SAVER) form should be completed and faxed or emailed along with the fax transmission form provided to the sponsor clinical safety and pharmacovigilance (CSPV) department. The SAVER form should be completed as soon as possible after EDC is available. For additional instructions, refer to the CRF Completion Guidelines (CCG).

See Section 15.10 for contact information for SAE reporting. If there are any questions related to SAE reporting, contact should be made to the emergency address or the monitor.

9.7 Notifying Regulatory Authorities, Investigators/Subinvestigators, and Institutional Review Boards/Ethics Committees

The sponsor and/or the contract research organization (CRO) will inform investigators or subinvestigators, institutional review boards/ethics committees (IRBs/ECs), and regulatory authorities of any suspected unexpected serious adverse reactions (SUSARs) occurring at a study site of this study or a study site of other studies using investigational products containing the same ingredients, as appropriate per local reporting requirements.

The sponsor and/or the CRO will comply with any additional local safety reporting requirements. To judge "Unexpected (unknown)," the section of "Reference Safety Information" in the updated IB or the document describing the latest scientific knowledge about the study drug should be referred to.

9.8 Exposure in Utero

The sponsor must be notified of any subject who becomes pregnant within 28 days from the study drug administration.

The sponsor must be notified of any male subject whose female partner becomes pregnant within 28 days from the study drug administration to the male subject.

Although pregnancy is not technically an AE, all pregnancies must be followed until delivery to determine their outcome. This information is important for both drug safety and public health concerns. It is the responsibility of the investigator or subinvestigator to report any pregnancy in a female subject or a male subject's female partner using the Exposure In Utero (EIU) Reporting form. Please contact your monitor to receive the EIU Reporting Form upon learning of a pregnancy. The investigator or subinvestigator should make every effort to follow the subject or the subject's partner until completion of the pregnancy and complete the EIU Reporting Form with complete pregnancy outcome information, including normal delivery and induced abortion. The outcome of the pregnancy, whether serious or nonserious, should be reported to the sponsor. If the outcome of the pregnancy meets the criteria for immediate classification as an SAE (ie, post-partum complications, spontaneous or induced abortion, stillbirth, neonatal death, or congenital anomaly, including that in miscarriage or an aborted fetus), the investigator or subinvestigator should follow the procedures for reporting SAEs

outlined in Section 9.6.

For reports of pregnancy in the female partner of a male subject, the EIU form (or SAE form if associated with an adverse outcome) should be completed with the subject numbers, initials, and date of birth, and details regarding the female partner should be entered in the narrative section.

9.9 Clinical Laboratory Evaluations

The following items will be measured:

The presence or absence of measurement and the date of measurement will be recorded in the eCRF.

- Hematology test
  Red blood cell count, hemoglobin, hematocrit, white blood cell count, differential white blood cell count (neutrophils, lymphocytes, monocytes, eosinophils, and basophils), and platelet count
- Blood chemistry test
  Total protein, albumin, total bilirubin, direct bilirubin, AST, ALT, ALP, gamma-glutamyltransferase (γ-GT), creatine kinase (CK), blood urea nitrogen (BUN), creatinine, Na, K, Cl, total cholesterol, and triglyceride
- Urinalysis
  Sugar, protein, occult blood, and urobilinogen

9.10 Vital Signs

The following items will be measured:

- Axillary body temperature
  The subject or legal representative will measure the axillary body temperature of the subject every day for 7 days after study drug administration (Day 1 to Day 8) and record the results in the electronic diary. On the day of study drug administration (Day 1), axillary body temperature will be measured once on the day. On Day 2 to Day 8, axillary body temperature will be measured once a day. If it is measured at multiple times a day, the maximal body temperature will be recorded. The temperature should not be taken immediately after eating, exercising, or taking a bath.

9.11 Electrocardiograms

Not applicable.

9.12 Physical Examinations

Not applicable.

9.13 Other Examinations

Not applicable.

10. OTHER EVALUATIONS

10.1 Patient-Reported Outcome

Among the AEs in Section 9.3, solicited AEs will be evaluated based on the patient-reported outcome obtained from the electronic diary.

11. STATISTICAL METHODS

11.1 General Statistical Considerations

The study population consists of the following 4 sub-population:

- Sub-population A: Population with a history of SARS-CoV-2 infection* and vaccination with SARS-CoV-2 vaccine
- Sub-population B: Population with a history of SARS-CoV-2 infection but without a history of vaccination with SARS-CoV-2 vaccine
- Sub-population C: Population without a history of SARS-CoV-2 infection but with a history of vaccination with SARS-CoV-2 vaccine
- Sub-population D: Population without a history of SARS-CoV-2 infection or vaccination with SARS-CoV-2 vaccine

*: The history of SARS-CoV-2 infection will be based on the self-reported SARS-CoV-2 infection history at informed consent and the SARS-CoV-2 antibody test result as measured at the central laboratory at the time of eligibility evaluation.

The primary endpoint of this study will be verified in subject population with at least either a history of SARS-CoV-2 infection or vaccination with SARS-CoV-2 vaccine (ABC combined) if the following conditions are met simultaneously:

- The lower limit of 2-sided 95% confidence interval of GMT ratio (DS-5670d group vs. Comirnaty RTU IM group) for blood neutralizing activity against SARS-CoV-2 (Omicron strain XBB.1.5.6) at 4 weeks after study drug administration is greater than 0.67.
- The lower limit of 2-sided 95% confidence interval of the difference in seroresponse rate (DS-5670d group − Comirnaty RTU IM group) for blood neutralizing activity against SARS-CoV-2 (Omicron strain XBB.1.5.6) at 4 weeks after study drug administration is greater than −10%.

In addition, the key secondary endpoints of this study will be validated in subject population with or without a history of SARS-CoV-2 infection or vaccination with SARS-CoV-2 vaccine (ABCD combined) if the following conditions are met simultaneously:

- The lower limit of 2-sided 95% confidence interval of GMT ratio (DS-5670d group vs. Comirnaty RTU IM group) for blood neutralizing activity against SARS-CoV-2 (Omicron strain XBB.1.5.6) at 4 weeks after study drug administration is greater than 0.67.
- The lower limit of 2-sided 95% confidence interval of the difference in seroresponse rate (DS-5670d group − Comirnaty RTU IM group) for blood neutralizing activity against SARS-CoV-2 (Omicron strain XBB.1.5.6) at 4 weeks after study drug administration is greater than −10%.

Multiplicity of the test associated with multiple non-inferiority hypotheses in multiple populations (ABC combined and ABCD combined) will be controlled by fixed sequence testing. First, the non-

inferiority of the DS-5670 group to the Comirnaty RTU IM group will be tested in the ABC combined population, and only if the non-inferiority is confirmed, the non-inferiority of the DS-5670d group to the Comirnaty RTU IM group will be verified in the ABCD combined population. All hypotheses will be evaluated at a one-sided 2.5% significance level.

With reference to the PMDA notification "Principles for the Evaluation of Vaccines Against the Novel Coronavirus SARS-CoV-2, (Appendix 4) Immunogenicity-based evaluation of variant vaccines modified from parent vaccines and booster vaccines with new active ingredients,"[31] the non-inferiority margins for the GMT ratio and the difference in the seroresponse rate are set at 0.67 and −10%, respectively.

In this study, analyses will be performed twice. The first analysis will be the primary analysis of immunogenicity and safety. The primary immunogenicity and safety endpoints will be evaluated to verify the non-inferiority of DS-5670d group to Comirnaty RTU IM group once when immunogenicity and safety data are collected for all subjects for 4 weeks after study drug administration or when the study is terminated. The second analyses will be performed when or the immunogenicity and safety data for 26 weeks after study drug administration have been obtained for all subjects or the study termination.

Unless otherwise specified, continuous variables will be summarized by presenting the number of subjects, arithmetic mean, standard deviation, minimum, median, and maximum, and categorical variables will be summarized by calculating the frequency and proportion. Unless otherwise specified, baseline values from each subject will be the last observed values that are available before study drug administration. Changes from baseline (eg, change, shift table) will be analyzed in subjects whose baseline values and at least one post-treatment value are available. An outline of the statistical analyses is provided below. Statistical methods will be specified in more details in separately prepared Statistical Analysis Plan (SAP).

11.2 Analysis Sets

Subjects with major GCP violations (violation of informed consent obtainment and significant violations of study procedures) will be excluded from all analysis sets.

- All subjects enrolled in the study must have signed the informed consent form by the subjects or legal representatives.
- All randomized subjects must have signed the informed consent form by the subjects or legal representatives.
- The safety analysis set will consist of all subjects who have signed the informed consent form by the subjects or legal representatives and received at least one dose of study drug.
- The solicited safety analysis set will consist of all subjects with at least one available data on presence or absence of occurrence of solicited AE in the safety analysis set.

- The full analysis set (FAS) will consist of randomized subjects who meet the following criterion:
  1) Having received at least one dose of the study drug.
- The per-protocol set (PPS) will consist of subjects included in the FAS who meet all of the following criteria:
  1) Having no significant protocol violations that may affect the efficacy evaluation (eg, violation of inclusion/exclusion criteria, prohibited concomitant medications/therapies, violation of withdrawal/discontinuation criteria).
- The FAS for immunogenicity evaluation (FAS for Immunogenicity) will consist of subjects included in the FAS who meet the following criterion:
  1) Having immunogenicity measurements before study drug administration and at least one immunogenicity measurement after study drug administration.
- The PPS for immunogenicity evaluation (PPS for Immunogenicity) will consist of subjects included in the FAS for Immunogenicity who meet the following criterion:
  1) Having no significant protocol violations that may affect the immunogenicity evaluation (eg, violation of inclusion/exclusion criteria, prohibited concomitant medications/therapies, violation of withdrawal/discontinuation criteria).
- The FAS with basic immunity (Immunized FAS) will consist of subjects included in the FAS who meet the following criterion:
  1) Having at least either a history of SARS-CoV-2 infection* or vaccination with SARS-CoV-2 vaccine and completed the study drug administration as specified in the protocol.

  *: The history of SARS-CoV-2 infection will be based on the self-reported SARS-CoV-2 infection history at informed consent and the SARS-CoV-2 antibody test result as measured at the central laboratory at the time of eligibility evaluation. The subject will be defined as having a history of SARS-CoV-2 infection based on self-reported SARS-CoV-2 infection history at informed consent or as having had a positive SARS-CoV-2 antibody test as measured at the central laboratory at the time of eligibility evaluation. Otherwise, the subject will be defined as having no history of SARS-CoV-2 infection.
- The per-protocol set with basic immunity (Immunized PPS) will consist of subjects included in the Immunized FAS who meet the following criterion:
  1) Having no significant protocol violations that may affect the efficacy evaluation (eg, violation of inclusion/exclusion criteria, prohibited concomitant medications/therapies, violation of withdrawal/discontinuation criteria).
- The FAS with basic immunity for immunogenicity evaluation (Immunized FAS for Immunogenicity) will consist of subjects included in the Immunized FAS who meet the following criterion:
  1) Having immunogenicity measurements before study drug administration and at least one

immunogenicity measurement after study drug administration.

- The PPS with basic immunity for immunogenicity evaluation (Immunized PPS for Immunogenicity) will consist of subjects included in the Immunized FAS for Immunogenicity who meet the following criterion:
  1) Having no significant protocol violations that may affect the immunogenicity evaluation (eg, violation of inclusion/exclusion criteria, prohibited concomitant medications/therapies, violation of withdrawal/discontinuation criteria).

11.3 Subject Data

In all enrolled subjects, frequency tabulation will be performed for randomized subjects, subjects who received the study drug, subjects who did not receive the study drug, subjects who completed the study, and subjects who have withdrawn from the study and reasons for withdrawal. In randomized subjects, frequency tabulation will also be made for inclusion/exclusion in/from each analysis set, and reasons for exclusion. Baseline characteristics will be tabulated for subjects included in the safety analysis set, solicited safety analysis set, FAS, PPS, FAS for Immunogenicity, PPS Immunogenicity, Immunized FAS, Immunized PPS, Immunized FAS for Immunogenicity, and Immunized PPS for Immunogenicity. The use of concomitant medications will also be tabulated for subjects included in the safety analysis set.

11.4 Efficacy Analysis

The primary analysis set for immunogenicity endpoints is the Immunized PPS for Immunogenicity or PPS for Immunogenicity, and a supplementary analysis will also be performed in the Immunized FAS for Immunogenicity or FAS for Immunogenicity.

In addition, the primary analysis set for COVID-19 incidence is the Immunized PPS or the PPS, and a supplementary analysis will also be performed in the Immunized FAS or the FAS. The analyses will be performed based on the treatment group to which each subject was assigned.

11.4.1 Primary Efficacy Analysis

The following analyses will be performed for the Immunized PPS for Immunogenicity.

The GMT ratio (DS-5670d group vs. Comirnaty RTU IM group) for blood neutralizing activity against SARS-CoV-2 (Omicron strain XBB.1.5.6) at 4 weeks after study drug administration and its 2-sided 95% confidence interval will be calculated by administration arm. The difference in seroresponse rate (DS-5670d group − Comirnaty RTU IM group) for blood neutralizing activity against SARS-CoV-2 (Omicron strain XBB.1.5.6) at 4 weeks after study drug administration and its 2-sided 95% confidence interval will be calculated.

A linear model will be applied with blood neutralizing activity against SARS-CoV-2 (Omicron strain XBB.1.5.6) at 4 weeks after study drug administration as an explained variable, administration

arm as an explanatory variable, and baseline value (common logarithmic value), a history of SARS-CoV-2 infection and vaccination with SARS-CoV-2 vaccine as covariates, based on which the GMT ratio (DS-5670d group vs. Comirnaty RTU IM group) and its 2-sided 95% confidence interval will be calculated. The covariates may be changed depending on subject enrollment.

In addition, the difference in seroresponse rate (DS-5670d group − Comirnaty RTU IM group) for blood neutralizing activity against SARS-CoV-2 (Omicron strain XBB.1.5.6) at 4 weeks after study drug administration and its 2-sided 95% confidence interval will be calculated. This analysis may be changed to stratified analysis depending on subject enrollment.

DS-5670d will be judged to be non-inferior to Comirnaty RTU IM if the lower limit of 2-sided 95% confidence interval in adjusted GMT ratio (DS-5670d group vs. Comirnaty RTU IM group) is greater than 0.67 and simultaneously the lower limit of 2-sided 95% confidence interval of the difference in seroresponse rate (DS-5670d group − Comirnaty RTU IM group) is greater than −10%.

The same supplementary analyses will also be performed for the Immunized FAS for Immunogenicity.

11.4.2 Secondary Efficacy Analysis

11.4.2.1 Key Secondary Efficacy Analysis

The same analysis as the primary efficacy analysis will be performed for the PPS for Immunogenicity. The same supplementary analyses will also be performed for the FAS for Immunogenicity.

11.4.2.2 Additional Secondary Efficacy Analysis

The incidence of COVID-19 (number of cases per 1000 person-years) for 4 weeks, 12 weeks, and 26 weeks after study drug administration will be calculated. The cumulative incidence will be calculated by administration arm using the Kaplan-Meier method.

The ratios of incidence (DS-5670d group vs. Comirnaty RTU IM group) and their 2-sided 95% confidence intervals will be calculated.

11.4.3 Exploratory Efficacy Analysis

Subgroup analyses will be performed for immunogenicity (eg, GMT of blood neutralizing activity against SARS-CoV-2 [Omicron strain XBB.1.5.6], seroresponse rate, etc.) according to baseline characteristics (eg, sex, history of SARS-CoV-2 infection, vaccination with SARS-CoV-2 vaccine).

The GMTs of blood neutralizing activity against SARS-CoV-2 (Omicron strain XBB.1.5.6) at 8 weeks, 12 weeks, 16 weeks, 20 weeks, and 26 weeks after study drug administration and their 2-sided 95% confidence intervals and the seroresponse rates will be calculated.

The GMTs of blood neutralizing activity against SARS-CoV-2* at 4 weeks, 8 weeks, 12 weeks, 16 weeks, 20 weeks, and 26 weeks after study drug administration and their 2-sided 95% confidence intervals, and the seroresponse rates will be calculated.

*: Details of variants to be evaluated are specified in a separately prepared plan.

11.4.4 Pharmacokinetic/Pharmacodynamic Analysis

Not applicable.

11.4.5 Safety Analysis

Solicited AEs will be analyzed in the solicited safety analysis set, and other safety analyses will be performed in the safety analysis set. The analyses will be performed based on the study drug that was actually administered to each subject.

11.4.5.1 Analysis of Adverse Events

A treatment-emergent adverse event (TEAE) is defined as any AE that emerges up to 28 days after study drug administration and is not observed before study drug administration but occurs or worsens in severity after study drug administration.

AEs recorded on the AE page of the eCRF will be coded using the International Council for Harmonisation of Technical Requirements for Pharmaceuticals for Human Use (ICH) Medical Dictionary for Regulatory Activities (MedDRA).

11.4.5.1.1 Analysis of Solicited Adverse Events (Collected from the Electronic Diary)

The number and proportion of subjects with solicited AEs (injection site and systemic) that are reported within 7 days after study drug administration will be calculated by event and by administration arm. In addition, solicited AEs will be summarized according to their severity.

A listing for solicited AEs, including the severity, will be prepared for each subject.

In addition, summary statistics of the number of days from study drug administration to the onset of the AE and the duration of symptoms continued in days will be calculated.

For solicited systemic AEs (fever) within 7 days after study drug administration, summary statistics of the highest body temperature will be calculated. Subgroup analyses will be performed on the frequency of solicited AEs according to baseline characteristics (eg, sex, history of SARS-CoV-2 infection, vaccination with SARS-CoV-2 vaccine).

11.4.5.1.2 Analysis of Unsolicited Adverse Events

The number and proportion of subjects with unsolicited TEAEs that are reported within 28 days after study drug administration will be calculated by system organ class (SOC), by preferred term (PT), and by administration arm. In addition, unsolicited TEAEs will be summarized according to their severity and causality with the study drug. Similarly, the number and proportion of subjects with TEAEs leading to permanent discontinuation of study treatment or withdrawal from the study will be summarized in a table. Adverse reactions will be tabulated in a similar manner.

A listing for unsolicited AEs (including TEAEs), including the verbatim term, PT, SOC, severity, and causality with the study drug, will be prepared for each subject. Any other required data should be included in the listing.

Subgroup analyses will be performed on the frequency of unsolicited AEs according to baseline characteristics (eg, sex, history of SARS-CoV-2 infection, vaccination with SARS-CoV-2 vaccine). Similar subgroup analyses will also be performed for adverse reactions.

11.4.5.1.3 Analysis of Serious and Other Significant Adverse Events

A table on the number and proportion of subjects with serious TEAEs will be prepared. In the second analyses (analysis for data up to 26 weeks after study drug administration), analysis will be performed in the same way by replacing TEAEs with AEs.

Death, other SAEs, and other significant AEs (including those that led to permanent discontinuation of study treatment or withdrawal from the study) will be listed.

11.4.5.2 Analysis of Clinical Laboratory Data

For hematology and blood chemistry test data (quantitative values), summary statistics of the measurement and the change from baseline at each time point will be calculated, and the time course of the measurements will be graphically represented.

For urinalysis data (qualitative values), a shift table of the baseline and post-treatment value at each time point will be prepared.

11.4.5.3 Analysis of Vital Signs

For body temperature, summary statistics of the measurement and the change from the final measurement before administration at each time point will be calculated, and the time course and by-subject time course of the measurements will be graphically represented.

11.4.5.4 Analysis of Electrocardiographic Parameters

Not applicable.

11.4.6 Blind Review

Not applicable.

11.5 Interim Analysis

No interim analysis will be performed in this study.

11.6 Sample Size Determination

Subjects with at least either a history of SARS-CoV-2 infection or vaccination with SARS-CoV-2

vaccine (Sub-population A, B and C): 690 subjects (DS-5670d group: 345 subjects, Comirnaty RTU IM group: 345 subjects)

Among those, subjects with a history of SARS-CoV-2 infection and without a history of vaccination with SARS-CoV-2 vaccine (Sub-population B): 100 subjects (DS-5670d group: 50 subjects, Comirnaty RTU IM group: 50 subjects)

Subjects without a history of SARS-CoV-2 infection or vaccination with SARS-CoV-2 vaccine (Sub-population D): Enrolled as many as possible.

<Rationale>

The primary objective of this study is to verify the non-inferiority in terms of blood neutralizing activity against SARS-CoV-2 (Omicron strain XBB.1.5.6) in population with at least either a history of SARS-CoV-2 infection or vaccination with SARS-CoV-2 vaccine (ABC combined). In addition, the key secondary objective is to verify the non-inferiority in terms of blood neutralizing activity against SARS-CoV-2 (Omicron strain XBB.1.5.6) in population with or without a history of SARS-CoV-2 infection or vaccination with SARS-CoV-2 vaccine (ABCD combined). Non-inferiority will have been verified if the following conditions are met simultaneously:

- The lower limit of 2-sided 95% confidence interval of GMT ratio (DS-5670d group vs. Comirnaty RTU IM group) for blood neutralizing activity against SARS-CoV-2 (Omicron strain XBB.1.5.6) at 4 weeks after study drug administration is greater than 0.67.
- The lower limit of 2-sided 95% confidence interval of the difference in seroresponse rate (DS-5670d group − Comirnaty RTU IM group) for blood neutralizing activity against SARS-CoV-2 (Omicron strain XBB.1.5.6) at 4 weeks after study drug administration is greater than −10%.

The target sample size has been set taking into account of power for the non-inferiority hypotheses, and feasibility.

Power for the primary objective

With the sample size of 690 subjects in the population with at least either a history of SARS-CoV-2 infection or vaccination with SARS-CoV-2 vaccine (ABC combined) and an assignment ratio (DS-5670d group : Comirnaty RTU IM group) of 1:1, and assuming that the percentage of enrolled subjects who do not contribute to immunogenicity evaluation due to reasons such as exclusion from analysis set and subjects' withdrawal from the study is 10.0%, the GMT ratio (DS-5670d group vs. Comirnaty RTU IM group) for blood neutralizing activity against SARS-CoV-2 at 4 weeks after study drug administration is 1.0, and the common standard deviation of neutralizing activity (common

logarithmic value) is 0.533, the statistical power to detect the non-inferiority for GMT would be 98.2% at 1-sided significance level of 0.025. The standard deviation was determined based on the results of Study DS5670-103 and Study DS5670-146 to compare DS-5670a and Comirnaty IM (monovalent: original strain) in subjects aged 18 years and older.

On the other hand, assuming that the seroresponse rate for blood neutralizing activity against SARS-CoV-2 at 4 weeks after study drug administration is 80.0% in both the DS-5670d group and Comirnaty RTU IM group, the statistical power to detect the non-inferiority for seroresponse rate would be 87.2% at 1-sided significance level of 0.025.

Therefore, the power for the non-inferiority which simultaneously satisfies these primary endpoints is 85.6% (= 98.2% × 87.2%) assuming that the non-inferiority in terms of GMT and seroresponse rate are respectively independent.

Power for the key secondary objectives

With the sample size of 790 subjects (ABC combined: 690 subjects, D: 100 subjects) in total population (ABCD combined), and assuming that the GMT ratio (DS-5670d group/Comirnaty RTU IM group) for blood neutralizing activity against SARS-CoV-2 at 4 weeks after study drug administration is 1.0, and the seroresponse rate is 80.0%, the statistical power to detect the non-inferiority for GMT would be 99.1%, and the non-inferiority for seroresponse rate would be 91.2% at 1-sided significance level of 0.025.

Therefore, the power for the non-inferiority which simultaneously satisfies these key secondary endpoints is 90.4% (= 99.1% × 91.2%) assuming that the non-inferiority in terms of GMT and seroresponse rate are respectively independent.

Meanwhile, the target sample size (100 subjects [14.5% of ABC combined]) with a history of SARS-CoV-2 infection and without a history of vaccination with SARS-CoV-2 vaccine (Sub-population B) was set to enable certain evaluation that a single dose of DS-5670d induces sufficient immunity. At the time, the feasibility was considered because the proportion of individuals aged 12 years and older who have not received the vaccine is 12.9% (based on the results of vaccination by age class published by the Office of the Prime Minister[25]) and because 42.0% of the people aged 16 and older have anti-N antibodies that are induced by SARS-CoV-2 infection[26].

11.7 Statistical Analysis Process

Statistical analysis will be performed by the sponsor or the CRO in accordance with the protocol and the SAP. The SAP represents statistical methods and definitions for analyzing efficacy and safety data, and also describes methods for summarizing other data, including disposition, demographic and other baseline characteristics of subjects, exposure to the study drug, and concomitant medications. The SAP will also include a description of how missing, unused, and spurious data will be addressed.

To maintain the integrity of statistical analysis and study conclusion, the SAP will be finalized and fixed before unblinding.

All statistical analyses will be performed using SAS Version 9.4 or later (SAS Institute).

12. DATA INTEGRITY AND QUALITY ASSURANCE

The head of the study site and the investigator will permit monitoring by the sponsor, audits, IRB review, and regulatory inspections by providing direct access to all study-related records including source documents. The sponsor will conduct monitoring and audits and directly access study-related records including source documents at the study site to confirm that the study has been appropriately conducted and the data reliability is properly assured. The sponsor will discuss with the investigator in advance regarding the procedures for direct access to source documents.

12.1 Monitoring and Inspections

The sponsor, CRO monitors, and regulatory authority inspectors are responsible for contacting the investigator and visiting the study site for the purpose of inspecting the facilities and, upon request, inspecting the various records of the study (eg, eCRFs, source data, other pertinent documents).

The verification of adherence to the protocol, completeness, accuracy, and consistency of the data, and adherence to ICH-GCP and local regulations on the conduct of clinical research will be accomplished through a combination of onsite visits by the monitor and remote review of study data. The frequency of the monitoring visit will vary based on the activity at each study site. The monitor is responsible for inspecting the eCRFs and ensuring the completeness of the study essential documents. The monitor will have access to subject medical records and other study-related records needed to verify the entries on the eCRFs. More details are provided in the Monitoring Plan.

The monitor will inform the investigator of violations of the protocol, standard operating procedures (SOPs), GCP, and applicable regulations, and ensure that appropriate actions to prevent their recurrence are taken and documented.

The investigator should agree to take actions satisfactory to the sponsor in cooperation with the monitor when any problem is detected during the process of monitoring, and ensure that they are documented.

In accordance with ICH-GCP, local regulations and the sponsor's audit plans, this study may be selected for audit by representatives from the sponsor. Audit of study site facilities (eg, pharmacy, drug storage areas, laboratories) and review of study-related records will be conducted to evaluate the study conduct and compliance with the protocol, ICH-GCP, and applicable regulatory requirements. The investigator should respond to audit findings. In the event that a regulatory authority informs the investigator that it intends to conduct an inspection, the investigator should notify the sponsor immediately.

12.2 Data Collection

12.2.1 Preparation of Case Report Forms

The sponsor will supply eCRFs. The investigator or subinvestigator will ensure that an eCRF is completed for each subject who signed the ICF and underwent any screening procedure. If the subject

is not administered the study drug, the reason must be recorded in the eCRF. All data collected during the study must be recorded in individual, subject-specific eCRFs. CCG will be provided by the sponsor. All entries and corrections to eCRFs will be automatically recorded as an audit trail in the EDC system. The eCRF should be kept current to enable the monitor to review the subject's status throughout the course of the study.

The eCRF must be completed, reviewed, and signed or e-signed by the investigator. The investigator will sign and date the eCRF via the EDC system's electronic signature. These signatures will indicate that the investigator inspected or reviewed the data in the eCRF, data queries, and site responses, and agrees with the content.

Easy-to-understand vocabulary and terminology must be used for all the information and other materials to be used by subjects and study staff in the study.

12.2.2 Entry Precautions for the Completion of Case Report Forms

In this study, the eCRF (completed by the investigator), clinical laboratory data report (prepared by the central laboratory), electronic diary (completed by subjects or legal representatives), verification report documented by telephone contacts, and immunogenicity measurement result report (prepared by the institution for immunogenicity measurement) will be prepared.

In this study, eCRFs will be electronically completed using a system (EDC System). The eCRF (including audit trail) will be completed for each subject and that signed by the investigator will be handled as the original. A validated EDC system will be used.

The eCRF will be created for subjects who provided informed consent.

12.2.3 Format of the Electronic Diary

Electronic diary data will be collected using an application (data entry system) installed on the electronic clinical outcome assessment (eCOA) device. The electronic diary entry account will be prepared for each subject. An electronic diary entry device will also be lent to them, if necessary. The subject or legal representative will answer all question items in the electronic diary, and the investigator will review the answers using the EDC system. A validated eCOA entry system will be used.

12.3 Data Management

Each subject in the database will be identified by a unique subject identifier as defined by the sponsor.

To ensure the quality of clinical data across all subjects and study sites, a clinical data management review will be performed on subject data according to the specifications given to the sponsor and the CRO. Data will be vetted both electronically and manually for eCRFs and the data will be electronically vetted according to the data rules programmed within the application. Queries

generated by the rules and raised by reviewers will be generated by the EDC system. During this review, subject data will be checked for consistency, completeness, and any apparent discrepancies.

Data received from external sources such as the central laboratory will be reconciled with the clinical database.

SAEs in the clinical database will be reconciled with the safety database.

All AEs entered in eCRFs will be coded using MedDRA.

Medical history/complications will be coded using MedDRA.

Concomitant medications will be coded using the WHO Drug Global B3 format.

12.4 Study Documentation and Storage

The investigator will maintain a signature list of appropriately qualified persons to whom he/she has delegated study duties.

The investigator will maintain a confidential screening log that includes information of all potential study candidates and the date and outcome of the screening process.

The investigator will be expected to maintain an enrollment log of all subjects enrolled in the study, including their assigned study numbers.

The investigator will maintain a confidential subject identifier list. This confidential list of names of all subjects assigned study numbers on enrolling in the study allows the investigator to reveal the identity of any subject when necessary.

Source documents are original documents, data, and records from which the subject's eCRF data are obtained. These include but are not limited to hospital records, medical records, clinical laboratory and pharmacy records, electronic diaries, microfiches, X-rays, and correspondence.

Records of subjects, source documents, data correction forms, eCRFs, electronic diaries, inventory of study drugs, regulatory documents (eg, protocol and amendments, IRB/EC correspondence and approvals, approved and signed ICFs, investigator's agreement, study drug receipts, distribution and return records), and other sponsor correspondence pertaining to the study must be kept in appropriate study files at the study site (Trial Master File [TMF]). Source documents include all recordings and observations or notations of clinical activities and all records necessary for the evaluation and reconstruction of the clinical study. These records will be retained in a secure file for the period required by the facility or the study site. Prior to transfer or destruction of these records, the sponsor must be notified in writing and be given the opportunity to further retain such records.

Entries in the eCRF, if the following items are entered directly in it (ie, there are no previously written or electronically recorded data) and all entries in the electronic diary will be considered source data:

1) Free description column in each item (comment column, etc.)
2) Entries of "No" or "Not done" in each item
3) Purpose of use of concomitant medications and therapies

4) The severity, seriousness, date of outcome, and causality with study drug regarding AEs
5) Reasons for withdrawal

12.5 Record Keeping

The head of the study site and/or the person responsible for record keeping are responsible for maintaining a comprehensive and centralized filing system (TMF) of all study-related (essential) documentation, suitable for inspection at any time by representatives from the sponsor and/or applicable regulatory authorities. Essential documents include:

- Subject files: Containing completed eCRFs (copies), electronic diaries (copies), ICFs, and copies of supporting source documentation (if kept).
- Study files: Containing the protocol with all amendments, IBs, copies of relevant essential documents required prior to commencing a clinical study, and all correspondence to and from the IRB/EC and the sponsor.
- Records related to the study drugs: Containing acknowledgment of receipt at the study site, accountability records, documentation of returned study drugs and copies of the study drug accountability list, and applicable correspondence.

In addition, all original source documents supporting entries in the eCRFs must be maintained and be readily available.

All study-related essential documentation will be retained by the head of the study site or the person responsible for record keeping until at least 3 years after the last approval of marketing application in an ICH region and until there are no pending or contemplated marketing applications in an ICH region or until at least 3 years have elapsed since the formal termination of clinical development of the investigational product. These documents should be retained for a longer period; however, if required by the applicable regulatory requirements or by an agreement with the sponsor. It is the responsibility of the sponsor to inform the investigator/study site as to when these documents no longer need to be retained.

Subject's medical files should be retained in accordance with applicable legislation and in accordance with the maximum period of time specified by the hospital, institution, or private practice.

No study document should be destroyed without prior written agreement between the sponsor and the investigator. If the investigator transfers study records to another party or moves to another location, the investigator must notify the sponsor in writing of the newly assigned person and the new location.

13. FINANCING AND INSURANCE

13.1 Finances

Prior to starting the study, the investigator or the study site will sign a clinical study agreement with Daiichi Sankyo or the CRO. This agreement will include the financial information agreed upon by the parties.

13.2 Payment of Expenses, Compensation for Health Damage, and Insurance

The sponsor will take measures such as insurance to ensure that subjects will be compensated in the case of the occurrence of study-related health damage.

The payment of expenses, compensation for health damage, and insurance should be handled in a separate agreement with respect to the conditions agreed upon between the parties.

It should be noted that a supplementary explanation regarding the payment of expenses, compensation for health damage, and insurance is provided in Attachment 1.

14. PUBLICATION OF RESULTS AND DISCLOSURE OF CLINICAL STUDY INFORMATION

Daiichi Sankyo is committed to meeting the highest standards of publication and public disclosure of information arising from clinical studies sponsored by the company. We will comply with US, EU, and Japanese policies for public disclosure of the clinical study information and clinical study results, and for sharing of clinical study data. We will follow the principles set forward in "Good Publication Practice for Communicating Company-Sponsored Medical Research (GPP3)," and publications will adhere to the "Recommendations for the Conduct, Reporting, Editing, and Publication of Scholarly Work in Medical Journals" established by the International Committee of Medical Journal Editors (ICMJE).

In order to ensure that we are in compliance with the public disclosure policies and the ICMJE recommendations, and to protect proprietary and confidential information generated during the study, all publications (manuscripts, abstracts, or other public disclosure) based on data generated in this study must be accepted, reviewed, and approved in writing by the sponsor prior to submission.

15. ETHICS AND STUDY ADMINISTRATIVE INFORMATION

15.1 Compliance Statement, Ethics, and Regulatory Compliance

This study will be conducted in compliance with the protocol, the ethical principles that have their origin in the Declaration of Helsinki, the ICH consolidated Guideline E6 for GCP (CPMP/ICH/135/95), and applicable regulatory requirements including the following:

- European Commission Directive (2001/20/EC, April 2001)
- European Commission Directive (2005/28/EC, April 2005)
- "Ministerial Ordinance on Good Clinical Practice for Drugs," Ministry of Health, Labour and Welfare Ordinance No. 28 (dated 27 March 1997)
- Act on Securing Quality, Efficacy and Safety of Products Including Pharmaceuticals and Medical Devices (Enforced on 25 November 2014)
- Other applicable local regulations

Data collection using the EDC system will be implemented in accordance with the "Use of Electromagnetic Records and Electronic Signatures in Applications etc., for Approval of, or License for Pharmaceuticals, etc." (PFSB Notification No. 0401022 dated 01 April 2005).

15.2 Subject Confidentiality

The investigator, subinvestigator, and the sponsor will preserve the confidentiality of all subjects participating in the study, in accordance with GCP and local regulations.

The investigator must ensure that the subject's anonymity is maintained. In the eCRFs or other documents submitted to the sponsor or the CRO, the subject should be identified by a unique subject identifier as designated by the sponsor. Documents that are not for submission to the sponsor or the CRO (eg, signed ICF) should be kept in strict confidence by the investigator.

In compliance with the ICH-GCP Guidelines, it is required that the investigator and the study site permit authorized representatives of the company, regulatory authorities, and the IRB/EC direct access to review the subject's original medical records for verification of study-related procedures and data. The investigator is obligated to inform the subject and legal representative that his/her study-related records will be reviewed by the above-mentioned representatives without violating the confidentiality of the subject.

15.3 Informed Consent

The subject and legal representative will be given sufficient explanation of the purpose, methods, anticipated benefits, and potential risks of the study prior to their participation in the study. It is the investigator's responsibility to obtain written, voluntary informed consent from the subject or legal representative at the time specified in the protocol or before any study drugs are administered. Even when consent is obtained from the legal representative of the subject aged 12 to 17 years, the

investigator or subinvestigator should explain an outline of the study to the subject using the ICF or assent form, depending on his/her ability of understanding, and obtain written, voluntary informed consent whenever possible. The subject and legal representative should be given the opportunity to ask questions and receive satisfactory answers to his/her inquiries, and should have adequate time to decide whether or not to participate in the study. The assent form or ICF should be prepared in the local language(s) of potential subjects and legal representatives.

In obtaining and documenting informed consent, the investigator should comply with the applicable regulatory requirements and GCP, and adhere to the ethical principles that have their origin in the Declaration of Helsinki. The ICF and any revisions should be approved by the IRB or EC prior to being provided to potential subjects and legal representatives.

The ICF signed by the legal representative should be retained with the subject's medical records. When written consent is obtained from the subject aged 12 to 17 years, the written confirmation of willingness to continue study participation signed by the subject should also be retained with the subject's medical records. The ICF should be signed and personally dated by the legal representative and by the persons who conducted the informed consent discussion (not limited to the investigator alone). The original signed ICF form, and the original signed assent form, if necessary, should be retained in accordance with the institutional policy, and a copy of the signed ICF should be provided to the subject and legal representative. The date that informed consent was obtained should be recorded in the eCRF.

When the subject is aged 12 to 17 years, the parent or legal guardian must sign the ICF based on the informed consent document approved by the IRB prior to implementing protocol-related procedures, and when requested by the IRB/EC of each site, consent to study participation must be obtained from the subject.

15.4 Regulatory Compliance

The study protocol, written information for subjects and legal representatives, ICF (and assent form, if necessary), IB, written instructions for subjects, safety information of the study drug, subject recruitment procedures (eg, advertisements), information about payments and compensation available to subjects, and documentation evidencing the investigator's qualifications should be submitted to the IRB or EC for ethical review and approval according to local regulations prior to study initiation. The written approval should identify all documents reviewed by name and version.

Changes in the conduct of the study or planned analysis will be documented in a protocol amendment and/or the SAP.

The investigator and/or the sponsor must submit and, where necessary, obtain approval from the IRB or EC for all subsequent protocol amendments and changes to the ICF. The investigator should notify the IRB or EC of deviations from the protocol or SAEs occurring at the study site and other AE reports received from the sponsor/CRO in accordance with local procedures.

As required by local regulations, the sponsor's local regulatory affairs group or representative to whom this responsibility has been delegated will ensure all legal aspects are covered, and approval from the appropriate regulatory bodies are obtained, prior to study initiation. If changes to the initial protocol and other relevant study documents are made, the assigned personnel will also ensure that any revised documents are submitted to regulatory authorities and implementation of these changes happen only after approval by the relevant regulatory bodies, as required.

In the event of any prohibition or restriction imposed (eg, clinical hold) by applicable regulatory authorities in any area of the world, or if the investigator is aware of any new information which might influence the evaluation of the benefits and risks of the investigational product, the sponsor should be informed immediately.

The investigator will inform the sponsor immediately of any urgent safety measures taken by the investigator to protect the study subjects against any immediate hazard, and of any suspected/actual serious GCP non-compliance that the investigator becomes aware of.

15.5 Protocol Deviations

The investigator or subinvestigator should conduct the study in compliance with the protocol agreed to by the sponsor and, if required, by regulatory authorities, and approved by the IRB/EC.

A deviation from any protocol procedures or waiver to any stated criteria will not be allowed in this study except where necessary to eliminate immediate risks to the subject. The sponsor must be promptly notified of all intended or unintended deviations from the protocol (eg, inclusion/exclusion criteria, study drug administration, missed study visits).

The investigator or subinvestigator should document and explain any deviation from the approved protocol.

If a subject was ineligible for the study or received an incorrect dose or treatment, but had at least one dose of the study drug, data of the subject must be collected for safety purposes.

If applicable, the investigator should notify the IRB or EC of deviations from the protocol in accordance with local procedures.

15.6 Supply of New Information Affecting the Conduct of the Study

When new information becomes available that may adversely affect the safety of subjects or the conduct of the study, the sponsor will inform all investigators involved in the study, IRBs/ECs, and regulatory authorities of such information, and when needed, amend the protocol and/or written information for subjects and legal representatives.

The investigator should immediately inform the subjects and legal representatives whenever new information becomes available that may be relevant to the consent of the legal representatives or may influence the willingness of the subjects and legal representatives to continue participation in the study. Communications with the subjects and legal representatives should be documented in medical

records, etc., and it should be confirmed whether the subjects and legal representatives are willing to remain in the study.

If written information for subjects and legal representatives, etc., is revised, it must be re-approved by the IRB/EC. The investigator should obtain consent to continue study participation from the subjects and legal representatives with the revised ICF, etc., even if the subjects and legal representatives were already informed of the relevant information. The investigator or other responsible personnel who provided explanations and the subjects and legal representatives should sign and date the revised ICF.

15.7 Protocol Amendments

Any amendments to the study protocol that seem to be appropriate as the study progresses will be communicated to the investigator by the sponsor or the CRO. Changes made by such amendments will be documented in a "Summary of Changes" document. These protocol amendments will undergo the same review and approval process as the original protocol.

A protocol amendment may be implemented after it has been approved by the IRB/EC and by regulatory authorities where appropriate, unless immediate implementation of the change is necessary for subject safety.

15.8 Study Termination

When any of the conditions below are met, and the sponsor determines that it is difficult to continue the study, the sponsor will suspend the study partially or entirely at that time point. The sponsor will then determine whether the study should be terminated partially or entirely and record it in writing.

1) New information on the safety of the study drug or information on SAEs is obtained.
2) The sponsor, the study site, or the investigator becomes involved in any major GCP violation or any major protocol deviation.
3) Other new information is obtained during the study.

If termination of the entire study or a part of the study has been decided as a result of a consultation with the medical expert, etc., or termination of the entire study has been decided according to the recommendation by the advisory board, the sponsor will promptly notify the head of the study site in writing of the decision and the reason. The head of the study site will promptly notify the investigator and the IRB in writing of the decision and the reason.

If the study is terminated or suspended regardless of the reason, the investigator will promptly notify subjects participating in the study and legal representatives and perform appropriate action and tests/examinations to confirm the safety of the subjects.

15.9 Data and Safety Monitoring Board

Not applicable.

15.10 Address List

See Attachment 2 Address List

16. REFERENCES

1. World Health Organization [Internet]. WHO advice for international travel and trade in relation to the outbreak of pneumonia caused by a new coronavirus in China, [cited 2020 January 10]. Available from: https://www.who.int/news-room/articles-detail/who-advice-for-international-travel-and-trade-in-relation-to-the-outbreak-of-pneumonia-caused-by-a-new-coronavirus-in-china/
2. Zhu N, Zhang D, Wang W, et al. A Novel Coronavirus from Patients with Pneumonia in China, 2019. N Engl J Med. 2020;382(8): 727-33.
3. World Health Organization [Internet]. Statement on the second meeting of the International Health Regulations (2005) Emergency Committee regarding the outbreak of novel coronavirus (2019-nCoV) [cited 2020 January 30]. Available from: https://www.who.int/news-room/detail/30-01-2020-statement-on-the-second-meeting-of-the-international-health-regulations-(2005)-emergency-committee-regarding-the-outbreak-of-novel-coronavirus-(2019-ncov)
4. World Health Organization [Internet]. WHO Director-General's remarks at the media briefing on 2019-nCoV on 11 February 2020 [cited 2020 February 11]. Available from: https://www.who.int/director-general/speeches/detail/who-director-general-s-remarks-at-the-media-briefing-on-2019-ncov-on-11-february-2020
5. World Health Organization [Internet]. WHO Director-General's opening remarks at the media briefing on COVID-19 - 11 March 2020 [cited 2020 March 11]. Available from: https://www.who.int/director-general/speeches/detail/who-director-general-s-opening-remarks-at-the-media-briefing-on-covid-19---11-march-2020
6. World Health Organization. COVID-19 Weekly Epidemiological Update. Edition 157, published 25 August 2023.
7. Office for COVID-19 and Other Emerging Infectious Disease Control, Cabinet Secretariat, Government of Japan. COVID-19 Information and Resources [cited 20 September 2023]. Available from: https://corona.go.jp/dashboard/
8. National Institute of Infectious Diseases (NIID). Summary on SARS-CoV-2 variants of concern for increased infectivity/transmissibility and antigenic changes (No. 27). Available from: https://www.niid.go.jp/niid/ja/2019-ncov/2551-cepr/12000-sars-cov-2-27.html
9. National Institute of Infectious Diseases (NIID). Estimation of the detection rate of each strain/variant by genomic surveillance based on samples from private testing institutions. Available from: https://www.niid.go.jp/niid/ja/2019-ncov/2624-flu/12055-flu2-1-1.html
10. Yamasoba D, Uriu K, Plianchaisuk A, et al. Virological characteristics of the SARS-CoV-2 omicron XBB.1.16 variant. Lancet Infect Dis. 2023;23(6):655-6.
11. World Health Organization [Internet]. Statement on the antigen composition of COVID-19 vaccines. Available from: https://www.who.int/news/item/18-05-2023-statement-on-the-antigen-

composition-of-covid-19-vaccines

12. Food and Drug Administration [Internet]. Updated COVID-19 Vaccines for Use in the United States Beginning in Fall 2023. Available from: https://www.fda.gov/vaccines-blood-biologics/updated-covid-19-vaccines-use-united-states-beginning-fall-2023
13. The 47th Immunization and Vaccine Committee of the Health Sciences Council [Internet]. Vaccines to be used in the fall and winter of 2023. Available from: https://www.mhlw.go.jp/content/10900000/001108705.pdf
14. Vaccines and Related Biological Products Advisory Committee June 15, 2023 Meeting Presentation- Pfizer: 2023-2024 COVID19 Vaccine Formula- Clinical and Preclinical Supportive Data (fda.gov). Available from: https://www.fda.gov/media/169541/download
15. National Healthcare Policy Secretariat, Cabinet Office, Government of Japan. Status of efforts based on the strategy for strengthening systems to develop and produce vaccines (07 June 2022). Available from: http://www.kantei.go.jp/jp/singi/kenkouiryou/sanyokaigou/dai21/siryou1-3.pdf
16. Polack FP, Thomas SJ, Kitchin N, et al. Safety and Efficacy of the BNT162b2 mRNA Covid-19 Vaccine. N Engl J Med. 2020;383(27):2603-15.
17. Baden LR, Sahly HME, Essink B, et al. Efficacy and Safety of the mRNA-1273 SARS-CoV-2 Vaccine. N Engl J Med. 2021;384(5): 403-16.
18. National Institute of Infectious Diseases (NIID). Preliminary report of a case-control study investigating the efficacy of novel coronavirus vaccines (5th report): Efficacy of Omicron-targeting bivalent vaccines. [cited 13 December 2022]. Available from: https://www.niid.go.jp/niid/ja/2019-ncov/2484-idsc/11688-covid19-9999.html
19. Ministry of Health, Labour and Welfare [Internet]. Partial change approval for Omicron strain-targeting vaccines. Available from: https://www.mhlw.go.jp/stf/newpage_34956.html
20. Ministry of Health, Labour and Welfare [Internet]. Partial change approval for Omicron strain-targeting vaccines. Available from: https://www.mhlw.go.jp/stf/newpage_35190.html
21. Coronavirus (COVID-19) Update: FDA Authorizes Changes to Simplify Use of Bivalent mRNA COVID-19 Vaccines. [cited 2023 September 20]. Available from: https://www.fda.gov/news-events/press-announcements/coronavirus-covid-19-update-fda-authorizes-changes-simplify-use-bivalent-mrna-covid-19-vaccines
22. European Medicines Agency [Internet]. COVID-19: Commission authorises adapted COVID-19 vaccine for Member States' autumn vaccination campaigns. Available from: https://ec.europa.eu/commission/presscorner/detail/en/ip_23_4301
23. JCVI statement on the COVID-19 vaccination programme for autumn 2023, 26 May 2023. [cited 2023 September 20]. Available from: https://www.gov.uk/government/publications/covid-19-autumn-2023-vaccination-programme-jcvi-advice-26-may-2023/jcvi-statement-on-the-covid-19-vaccination-programme-for-autumn-2023-26-may-2023
24. Annabel P, Freja K, Julia S, et al. Protection against symptomatic infection with delta

(B.1.617.2) and omicron (B.1.1.529) BA.1 and BA.2 SARS-CoV-2 variants after previous infection and vaccination in adolescents in England, August, 2021–March, 2022: a national, observational, test-negative, case-control study. Lancet Infect Dis. 2023;23(4): 435-44

25. Cabinet Secretariat Cabinet Public Affairs Office, Office of Prime Minister [Homepage]. About the novel coronavirus vaccine [cited 18 January 2023]. Available from: https://www.kantei.go.jp/jp/headline/kansensho/vaccine.html
26. National Institute of Infectious Diseases (NIID). Analysis on prevalence of previous infection using donated blood samples as of February 2023. Available from: https://www.niid.go.jp/niid/ja/2019-ncov/2484-idsc/12061-covid19-84.html
27. Trial designs for New SARS-CoV-2 Vaccine. ICMRA COVID-19 Virus Variants Workshop. Available from: https://www.icmra.info/drupal/sites/default/files/2021-07/24june2021_trial_designs_for_new_sars-cov2_vaccines.pdf
28. Khoury DS, Cromer D, Reynaldi A, et al. Neutralizing antibody levels are highly predictive of immune protection from symptomatic SARS-CoV-2 infection. Nat Med. 2021;27(7):1205-11.
29. Office of Vaccines and Blood Products, Pharmaceuticals and Medical Devices Agency. Principles for the evaluation of vaccines against the novel coronavirus SARS-CoV-2 (02 September 2020). Available from: https://www.pmda.go.jp/files/000236327.pdf
30. Pharmaceutical Evaluation Division. Modification of Description on Dosage and Administration of COVID-19 Vaccines (March 4, 2024). Available from: https://www.mhlw.go.jp/content/11121000/001219101.pdf
31. Office of Vaccines and Blood Products, Pharmaceuticals and Medical Devices Agency. Principles for the evaluation of vaccines against the novel coronavirus SARS-CoV-2. (Appendix 4) Immunogenicity-based evaluation of variant vaccines modified from parent vaccines and booster vaccines with new active ingredients (15 July 2022). Available from: https://www.pmda.go.jp/files/000247491.pdf
32. U.S. Department of Health and Human Services, Food and Drug Administration, Center for Biologics Evaluation and Research. Guidance for Industry: Toxicity Grading Scale for Healthy Adult and Adolescent Volunteers Enrolled in Preventive Vaccine Clinical Trials. (September, 2007). Available from: https://www.fda.gov/media/73679/download
33. U.S. Department of Health and Human Services, Food and Drug Administration, Center for Biologics Evaluation and Research. Guidance for Industry: Drug-induced Liver Injury: Premarketing Clinical Evaluation. (July, 2009). Available from: https://www.fda.gov/media/116737/download

17. APPENDICES

17.1 Schedule of Event

Table 17.1-1 Investigations, Observations, Examinations, and Sampling Schedule

	Observation period						Follow-up period					At the time of withdrawal
	Day of administration		7 days	14 days	21 days	4 weeks	8 weeks	12 weeks	16 weeks	20 weeks	26 weeks	
Scheduled day	Day 1		Day 8	Day 15	Day 22	Day 29	Day 57	Day 85	Day 113	Day 141	Day 183	
Acceptable time window (days)	0		± 2	± 2	± 2	3	± 7	± 7	± 7	± 7	± 14	
Visit/Tel	Visit		Tel	Tel	Tel	Visit	Visit[d]	Visit	Visit[d]	Visit[d]	Visit	
Investigation/observation items	Pre-dose	Post-dose	-	-	-	-	-	-	-	-	-	
Informed consent	■											
Baseline subject characteristics	■											
Eligibility determination	■											
Pregnancy test[a]	■					■						(■)
Clinical laboratory tests (blood/urine)	■					■						(■)
SARS-CoV-2 antigen test	■											
SARS-CoV-2 antibody test	■					■	■	■	■	■	■	■
Blood sampling for immunogenicity evaluation	■					■	■	■	■	■	■	■
Study drug administration	■											
Evaluation of immediate reaction (30 minutes after administration)		■										
Electronic diary		←	—	—	—	→						
Solicited adverse events (AEs)		←	→									(■)
AEs		←	—	—	—	→						(■)
SAEs	←	—	—	—	—	—	—	—	—	—	→	■
Investigation of the onset of COVID-19[b]		←	—	—	—	—	—	—	—	—	→	■
Prohibition period of other medications and concomitant therapies[c]	←	—	—	—	—	→						

■: Mandatory, ■: To be performed whenever possible, (■): To be performed depending on the timing of withdrawal from the study.

a: To be performed for women of childbearing potential only.
b: During the investigation, subjects with symptoms suspected of COVID-19 should be instructed to visit the study site whenever possible and undergo the RT-PCR test using nasopharyngeal swab. For subjects tested for COVID-19 at other medical institutions, the result of the test (eg, RT-PCR, SARS-CoV-2 antigen test) should be obtained at a later date.
c: Use of SARS-CoV-2 vaccines (including drugs used in the study) will be prohibited during the period from informed consent to 26 weeks after study drug administration.
d: To be performed for subjects aged 12 to 17 years to the extent possible.
