# Supplementary figures and images for: Immunogenicity and safety of DS-5670d, an omicron XBB.1.5-targeting COVID-19 mRNA vaccine: A phase 3, randomized, active-controlled study
Source: PLoS Med. 2025 Oct 13;22(10):e1004499. doi: 10.1371/journal.pmed.1004499 (PMC12517495; doi:10.1371/journal.pmed.1004499)

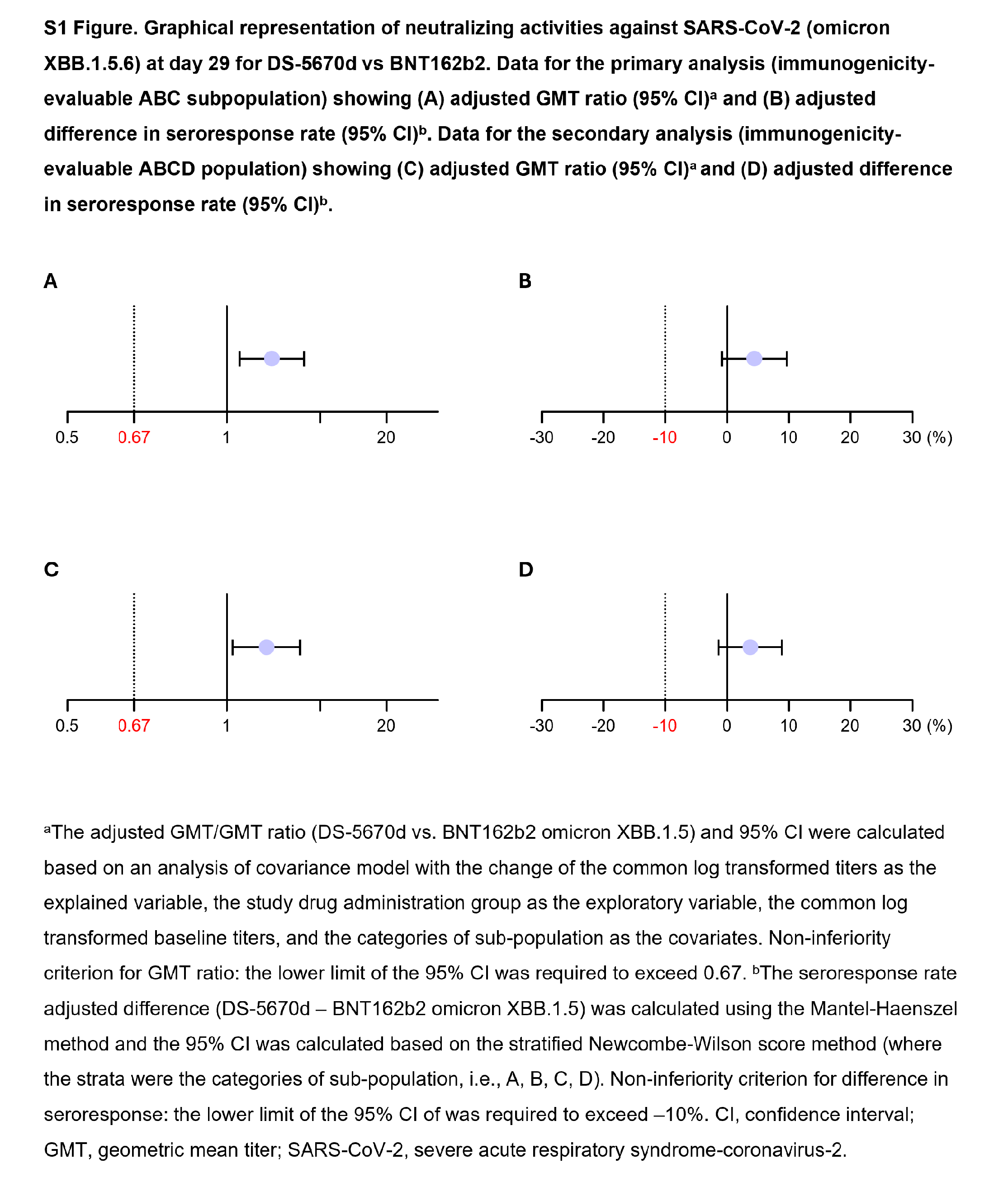

Supplement: S1 Fig — (TIF) [file pmed.1004499.s004.tif]

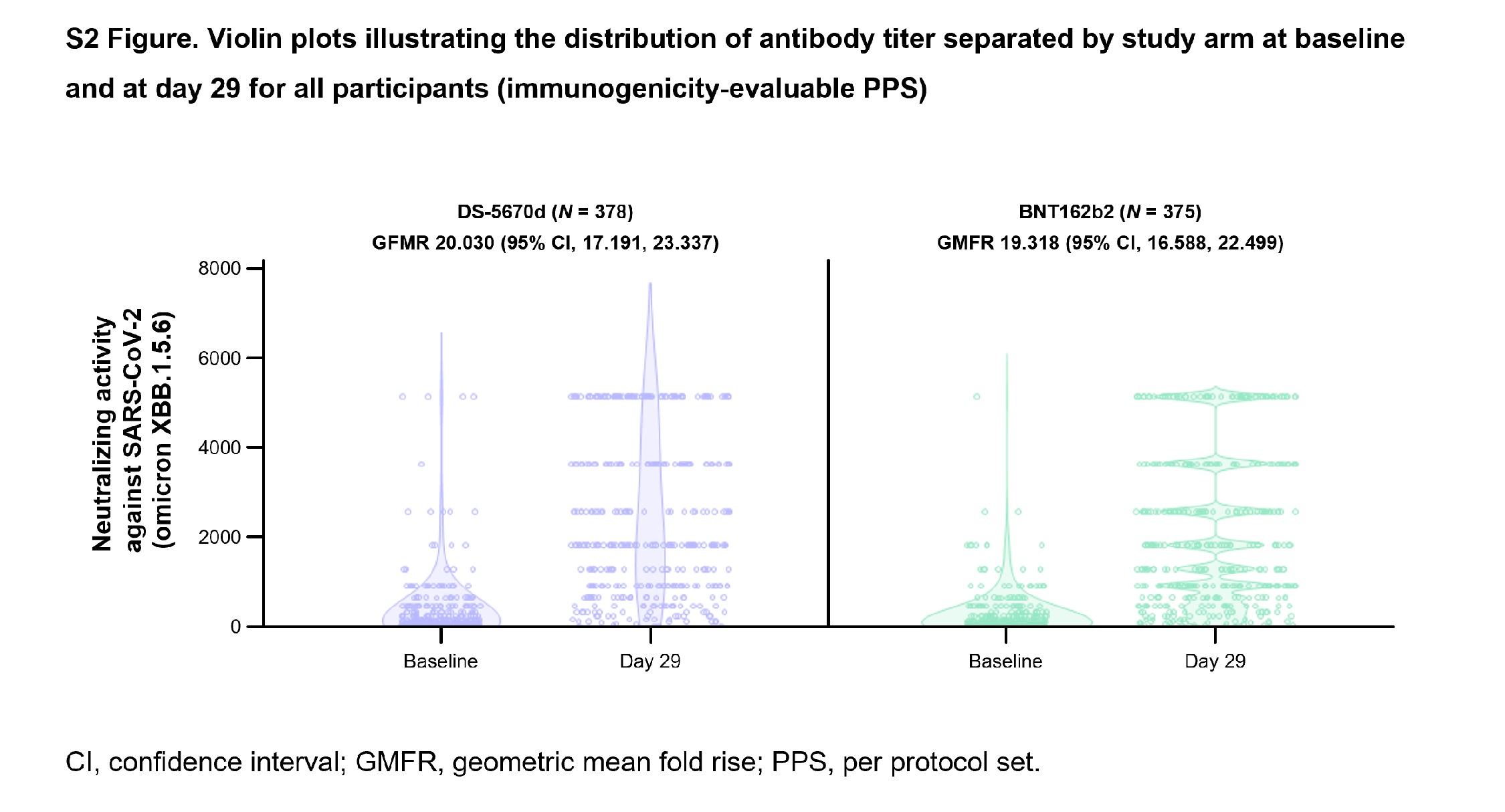

Supplement: S2 Fig — (TIF) [file pmed.1004499.s005.tif]

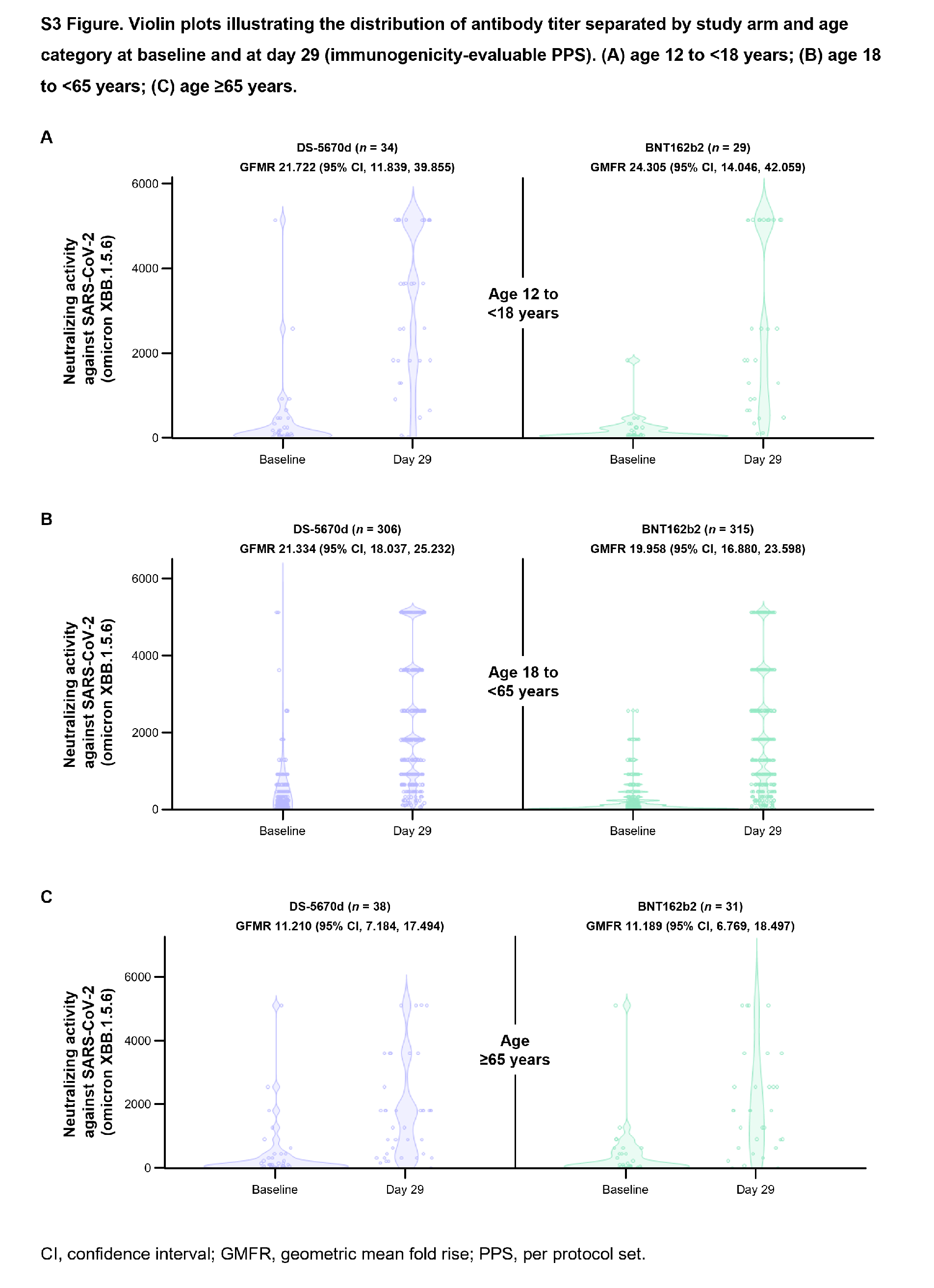

Supplement: S3 Fig — (TIF) [file pmed.1004499.s006.tif]

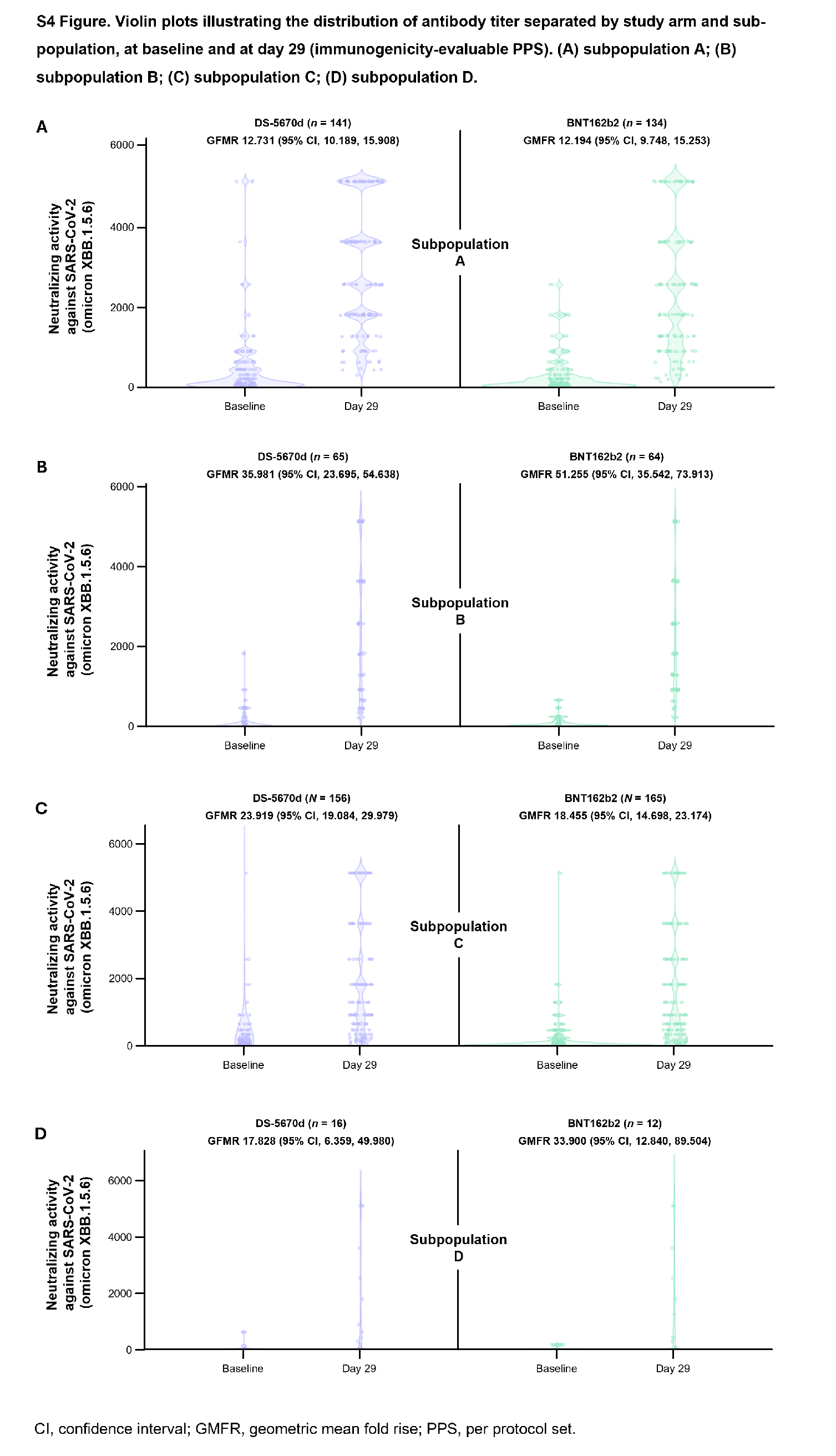

Supplement: S4 Fig — (TIF) [file pmed.1004499.s007.tif]

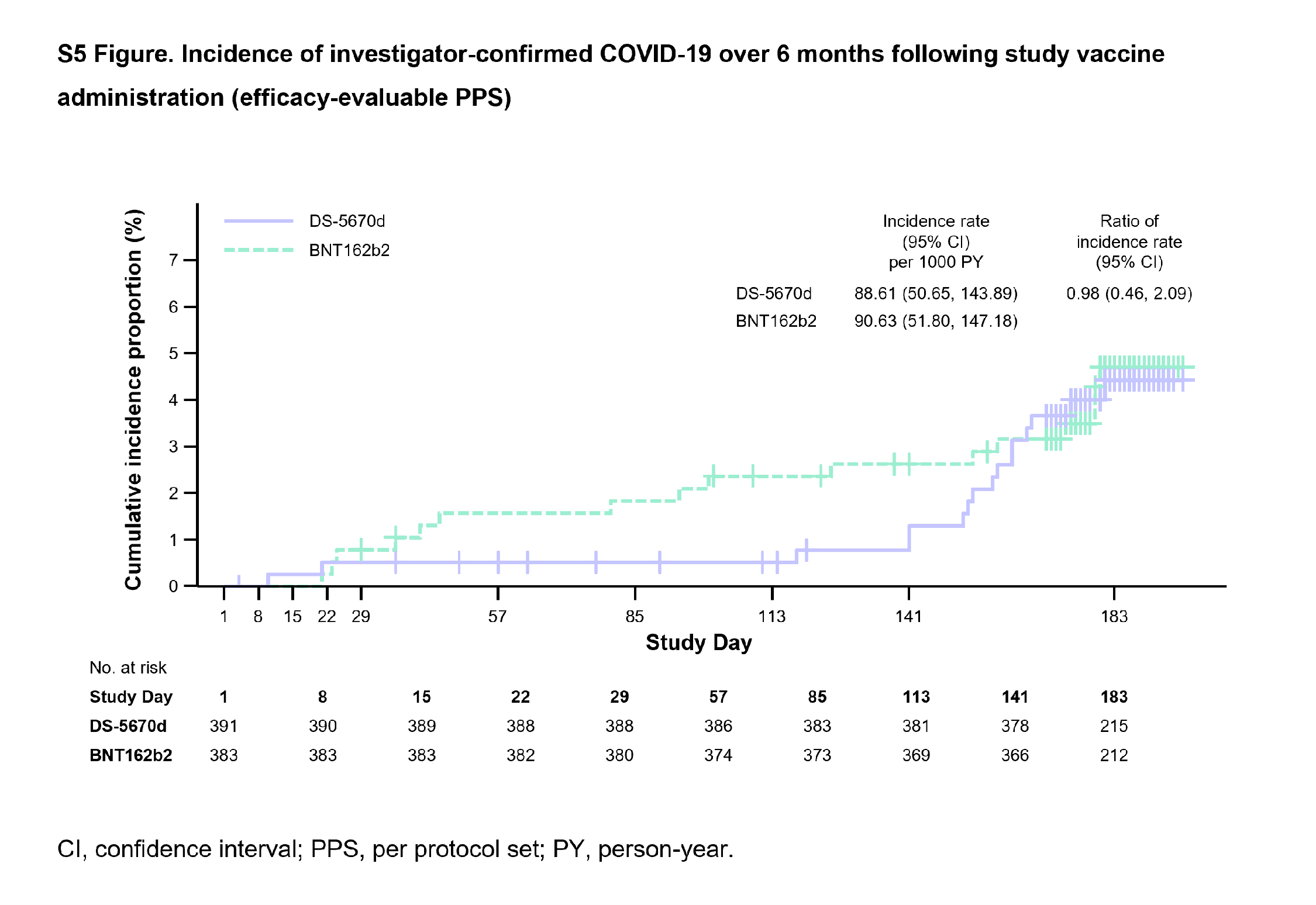

Supplement: S5 Fig — (TIF) [file pmed.1004499.s008.tif]
