# Supplementary material for: Immunogenicity and safety of DS-5670d, an omicron XBB.1.5-targeting COVID-19 mRNA vaccine: A phase 3, randomized, active-controlled study
Source: PLoS Med. 2025 Oct 13;22(10):e1004499. doi: 10.1371/journal.pmed.1004499 (PMC12517495; doi:10.1371/journal.pmed.1004499)
Supplement: S1 Table — (PDF) [file pmed.1004499.s009.pdf]

S1 Table. Listing of study sites and investigators.

Study site	Investigator	Ethical approval
Adachikyosai Byoin	Manabu Yamamoto	Adachi Kyosai Hospital, ethics committee
Den-En-Tyofu Family Clinic	Yoshihiro Umezawa	Nakameguro Atlas Clinic Institutional Review Board
Higashi Shinjuku Clinic	Hiroaki Kondo	Nakameguro Atlas Clinic Institutional Review Board
Irie Medical Clinic	Takashi Irie	Adachi Kyosai Hospital, ethics committee
Medical Corporation Chisei-Kai Tokyo Center Clinic	Aki Tsuji	Adachi Kyosai Hospital, ethics committee
Medical Corporation Heishinkai Ophac Hospital	Hidetoshi Furuie	Medical Corporation Heishinkai OPHAC Hospital Institutional Review Board
Medimesse Sakura Jyuji Clinic	Shokei Mitsuyama	Nakameguro Atlas Clinic Institutional Review Board
Nihonbashi Sakura Clinic	Kumie Ito	Adachi Kyosai Hospital, ethics committee
Onaka Naika Higashishirakabe Clinic	Yusuke Sugita	Adachi Kyosai Hospital, ethics committee
Sanpokai Nanko Clinic	Rintaro Takesako	Adachi Kyosai Hospital, ethics committee
Sapporo Odori Endoscopy Clinic	Shinya Mitsui	Nakameguro Atlas Clinic Institutional Review Board
Seishukai Clinic	Mamoru Oki	Adachi Kyosai Hospital, ethics committee
Souseikai Hakata Clinic	Takashi Eto	Hakata Clinic Institutional Review Board

Tenjin Sogo Clinic	Kenjiro Nakamura	Review Board of Human Rights and Ethics for Clinical Studies
Ueyama Child Clinic	Nami Ueyama	Nakameguro Atlas Clinic Institutional Review Board
