# Supplementary material for: Immunogenicity and safety of DS-5670d, an omicron XBB.1.5-targeting COVID-19 mRNA vaccine: A phase 3, randomized, active-controlled study
Source: PLoS Med. 2025 Oct 13;22(10):e1004499. doi: 10.1371/journal.pmed.1004499 (PMC12517495; doi:10.1371/journal.pmed.1004499)
Supplement: S2 Table — (PDF) [file pmed.1004499.s010.pdf]

S2 Table. Baseline characteristics according to subpopulation (safety analysis set).

	Subpopulation				All participants
	A<br><br>(*n* = 286)	B<br><br>(*n* = 133)	C<br><br>(*n* = 330)	D<br><br>(*n* = 28)	(*N* = 777)
Age (years)					
Median (range)	46.0 (12, 81)	32.0 (12, 73)	50.0 (12, 90)	46.5 (12, 73)	46.0 (12, 90)
12–17	20 (7.0)	15 (11.3)	23 (7.0)	6 (21.4)	64 (8.2)
18–65	248 (86.7)	116 (87.2)	261 (79.1)	19 (67.9)	644 (82.9)
≥65	18 (6.3)	2 (1.5)	46 (13.9)	3 (10.7)	69 (8.9)
Male sex	143 (50.0)	85 (63.9)	183 (55.5)	14 (50.0)	425 (54.7)
Weight (kg), median (range)	61.2 (35, 127)	61.6 (40, 132)	63.9 (37, 124)	60.6 (31, 93)	62.4 (31, 132)
Height (cm), median (range)	164.0 (140, 190)	165.7 (147, 182)	165.2 (146, 185)	161.3 (144, 180)	164.6 (140, 190)
History of SARS-CoV-2 infection	286 (100.0)	133 (100.0)	0 (0.0)	0 (0.0)	419 (53.9)
Self-reported prior infection	227 (79.4)	125 (94.0)	0 (0.0)	0 (0.0)	352 (45.3)
Positive antibody test[a]	110 (38.5)	48 (36.1)	0 (0.0)	0 (0.0)	158 (20.3)
History of COVID-19 vaccination					616 (79.3)
Original strain	6 (2.1)	0 (0.0)	22 (6.7)	0 (0.0)	313 (40.3)
Original/omicron bivalent	149 (52.1)	0 (0.0)	164 (49.7)	0 (0.0)	275 (35.4)

Omicron strain XBB.1.5	131 (45.8)	0 (0.0)	144 (43.6)	0 (0.0)	28 (3.6)
Interval from last vaccination (months)					
Median (range)	16.0 (3, 31)	-	15.4 (3, 34)	-	15.6 (3, 34)
3–11	45 (15.7)	0 (0.0)	51 (15.5)	0 (0.0)	96 (12.4)
≥12	240 (83.9)	0 (0.0)	279 (84.5)	0 (0.0)	519 (66.8)

Data are reported as *n* (%) unless otherwise stated. All participants (100%) were of Asian race. Subpopulations were defined as follows: (A) those with a history of both SARS-CoV-2 infection and COVID-19 vaccination, (B) those with a history of SARS-CoV-2 infection but without a history of COVID-19 vaccination, (C) those without a history of SARS-CoV-2 infection but with a history of COVID-19 vaccination, or (D) those without any history of SARS-CoV-2 infection or COVID-19 vaccination prior to the date of informed consent. [a]N -antibody positivity was centrally confirmed by an immunochromatographic test kit (Rapidfields S+N IgG [RF-NC003]; Kurabo Industries Ltd., Osaka, Japan) on day 1 prior to study vaccination. COVID-19, coronavirus disease 2019; SARS-CoV-2, severe acute respiratory syndrome-coronavirus-2.
