# Supplementary material for: Immunogenicity and safety of DS-5670d, an omicron XBB.1.5-targeting COVID-19 mRNA vaccine: A phase 3, randomized, active-controlled study
Source: PLoS Med. 2025 Oct 13;22(10):e1004499. doi: 10.1371/journal.pmed.1004499 (PMC12517495; doi:10.1371/journal.pmed.1004499)
Supplement: S3 Table — (PDF) [file pmed.1004499.s011.pdf]

S3 Table. Anonymized individual data underlying the neutralizing titer calculations, by sex (immunogenicity-evaluable ABCD population).

Administration arm	Sex	Subpopulation	Neutralizing antibody titer (XBB.1.5.6)	
			Day 1	Day 29
DS-5670d	Male	A	113.1	905.1
			905.1	1810.2
			452.5	3620.4
			160.0	1810.2
			80.0	1280.0
			5120.0	3620.4
			40.0	1280.0
			28.3	2560.0
			80.0	3620.4
			14.1	905.1
			160.0	1810.2
			40.0	3620.4
			40.0	5120.0
			320.0	1810.2
			40.0	3620.4
			905.1	5120.0
			56.6	640.0
			28.3	2560.0
			2560.0	5120.0
			113.1	3620.4
			905.1	3620.4
			226.3	3620.4
			2560.0	5120.0
			640.0	2560.0
			113.1	2560.0
			5120.0	3620.4
			113.1	640.0
			28.3	2560.0
			113.1	5120.0
			28.3	5120.0
			113.1	5120.0
			640.0	5120.0
			40.0	2560.0
			80.0	452.5
			640.0	3620.4
			640.0	5120.0
			2560.0	3620.4
			226.3	5120.0
			113.1	905.1
			226.3	905.1
			113.1	640.0
			452.5	1810.2
			226.3	2560.0
			905.1	5120.0
			452.5	1810.2
			113.1	452.5
			905.1	2560.0
			40.0	640.0
			320.0	5120.0
			640.0	1810.2
			113.1	5120.0
			320.0	3620.4
			80.0	2560.0
			452.5	1280.0
			226.3	3620.4
			80.0	905.1
			28.3	905.1
			56.6	5120.0
			28.3	5120.0
			80.0	5120.0

Administration arm	Sex	Subpopulation	Neutralizing antibody titer (XBB.1.5.6)	
			Day 1	Day 29
DS-5670d (cont.)	Male (cont.)	A (cont.)	56.6	2560.0
			226.3	1810.2
			1280.0	5120.0
			1280.0	5120.0
			10.0	1810.2
			320.0	5120.0
			226.3	3620.4
			40.0	5120.0
			640.0	5120.0
			640.0	3620.4
			56.6	1810.2
		B	10.0	2560.0
			14.1	452.5
			160.0	3620.4
			160.0	2560.0
			14.1	320.0
			80.0	1280.0
			56.6	905.1
			28.3	2560.0
			1810.2	5120.0
			56.6	5120.0
			56.6	226.3
			56.6	1810.2
			5.0	640.0
			5.0	452.5
			20.0	1810.2
			5.0	452.5
			226.3	2560.0
			7.1	905.1
			80.0	1810.2
			452.5	3620.4
			56.6	3620.4
			14.1	5120.0
			56.6	3620.4
			5.0	640.0
			5.0	3620.4
			56.6	1810.2
			905.1	1280.0
			5.0	452.5
			28.3	640.0
			452.5	5120.0
			28.3	3620.4
			452.5	5120.0
			10.0	3620.4
			452.5	3620.4
			7.1	905.1
			80.0	452.5
			10.0	1810.2
			5.0	1280.0
			5.0	5120.0
			5.0	452.5
			10.0	5120.0
			640.0	905.1
			5.0	1280.0
			320.0	5120.0
		C	5.0	1280.0
			10.0	1810.2
			226.3	1280.0
			14.1	1280.0
			160.0	2560.0

Administration arm	Sex	Subpopulation	Neutralizing antibody titer (XBB.1.5.6)	
			Day 1	Day 29
DS-5670d (cont.)	Male (cont.)	C (cont.)	20.0	1810.2
			10.0	5120.0
			40.0	80.0
			160.0	1810.2
			80.0	5120.0
			80.0	905.1
			14.1	1280.0
			7.1	5120.0
			160.0	320.0
			7.1	160.0
			226.3	1810.2
			640.0	905.1
			56.6	1810.2
			1810.2	905.1
			113.1	1810.2
			5.0	452.5
			56.6	1280.0
			5.0	452.5
			5.0	160.0
			7.1	160.0
			28.3	905.1
			28.3	3620.4
			113.1	5120.0
			10.0	1280.0
			5.0	320.0
			7.1	320.0
			20.0	1810.2
			160.0	5120.0
			14.1	226.3
			320.0	2560.0
			40.0	160.0
			56.6	1810.2
			20.0	640.0
			80.0	2560.0
			320.0	452.5
			56.6	905.1
			40.0	5120.0
			452.5	3620.4
			1810.2	3620.4
			320.0	905.1
			226.3	905.1
			640.0	5120.0
			56.6	452.5
			640.0	5120.0
			905.1	3620.4
			160.0	3620.4
			320.0	1810.2
			56.6	5120.0
			14.1	1280.0
			226.3	5120.0
			5.0	113.1
			20.0	905.1
			56.6	5120.0
			20.0	226.3
			2560.0	5120.0
			40.0	5120.0
			40.0	905.1
			28.3	1810.2
			14.1	1280.0
			905.1	5120.0

Administration arm	Sex	Subpopulation	Neutralizing antibody titer (XBB.1.5.6)	
			Day 1	Day 29
DS-5670d (cont.)	Male (cont.)	C (cont.)	7.1	160.0
			56.6	1810.2
			56.6	1810.2
			113.1	1810.2
			28.3	1810.2
			5.0	640.0
			5120.0	5120.0
			14.1	226.3
			56.6	2560.0
			5.0	56.6
			40.0	640.0
			5.0	226.3
			40.0	5120.0
			5.0	7.1
			28.3	3620.4
			28.3	226.3
			7.1	640.0
			40.0	1810.2
			14.1	226.3
			226.3	3620.4
			80.0	3620.4
			10.0	640.0
		D	20.0	3620.4
			7.1	113.1
			56.6	226.3
			5.0	56.6
			7.1	320.0
			5.0	7.1
			40.0	452.5
	Female	A	56.6	1810.2
			80.0	5120.0
			28.3	1280.0
			80.0	640.0
			113.1	640.0
			14.1	1810.2
			113.1	452.5
			226.3	1810.2
			226.3	2560.0
			226.3	1810.2
			80.0	905.1
			160.0	1810.2
			113.1	3620.4
			113.1	905.1
			28.3	3620.4
			160.0	1810.2
			56.6	5120.0
			452.5	5120.0
			113.1	1810.2
			640.0	1280.0
			28.3	3620.4
			160.0	640.0
			226.3	452.5
			226.3	905.1
			14.1	320.0
			160.0	5120.0
			452.5	3620.4
			1280.0	3620.4
			905.1	5120.0
			80.0	3620.4
			3620.4	2560.0

Administration arm	Sex	Subpopulation	Neutralizing antibody titer (XBB.1.5.6)	
			Day 1	Day 29
DS-5670d (cont.)	Female (cont.)	A (cont.)	452.5	3620.4
			56.6	5120.0
			320.0	1810.2
			28.3	1810.2
			56.6	3620.4
			452.5	1810.2
			1280.0	2560.0
			452.5	3620.4
			452.5	1280.0
			905.1	5120.0
			905.1	5120.0
			320.0	3620.4
			113.1	5120.0
			113.1	1810.2
			905.1	5120.0
			452.5	2560.0
			56.6	905.1
			452.5	2560.0
			320.0	2560.0
			1810.2	5120.0
			320.0	1810.2
			28.3	1810.2
			40.0	1280.0
			905.1	1280.0
			452.5	1810.2
			452.5	5120.0
			14.1	905.1
			80.0	1810.2
			640.0	5120.0
			640.0	905.1
			452.5	5120.0
			320.0	5120.0
			5120.0	5120.0
			226.3	1810.2
			14.1	3620.4
			113.1	2560.0
			905.1	3620.4
			14.1	905.1
			28.3	3620.4
			80.0	1280.0
		B	5.0	2560.0
			80.0	5120.0
			28.3	5120.0
			226.3	3620.4
			113.1	320.0
			320.0	452.5
			7.1	640.0
			113.1	226.3
			40.0	905.1
			452.5	1810.2
			14.1	905.1
			226.3	2560.0
			5.0	1810.2
			80.0	1280.0
			113.1	5120.0
			20.0	2560.0
			452.5	1810.2
			905.1	1810.2
			40.0	640.0
			20.0	905.1

Administration arm	Sex	Subpopulation	Neutralizing antibody titer (XBB.1.5.6)	
			Day 1	Day 29
DS-5670d (cont.)	Female (cont.)	B (cont.)	14.1	905.1
			452.5	3620.4
		C	1280.0	5120.0
			226.3	640.0
			40.0	905.1
			905.1	1810.2
			40.0	905.1
			5.0	320.0
			7.1	640.0
			5.0	113.1
			905.1	5120.0
			113.1	640.0
			20.0	1280.0
			5.0	905.1
			14.1	3620.4
			160.0	1810.2
			320.0	2560.0
			5.0	113.1
			56.6	113.1
			56.6	5120.0
			226.3	3620.4
			160.0	1810.2
			80.0	5120.0
			5.0	1280.0
			160.0	905.1
			7.1	640.0
			5.0	320.0
			10.0	905.1
			113.1	5120.0
			160.0	1810.2
			14.1	905.1
			5.0	113.1
			5.0	1810.2
			5.0	320.0
			452.5	3620.4
			80.0	1810.2
			5.0	905.1
			160.0	5120.0
			14.1	320.0
			20.0	2560.0
			20.0	3620.4
			80.0	5120.0
			14.1	905.1
			320.0	3620.4
			160.0	2560.0
			28.3	1810.2
			28.3	5120.0
			5.0	226.3
			160.0	5120.0
			160.0	5120.0
			14.1	5120.0
			56.6	1810.2
			5.0	113.1
			80.0	5120.0
			320.0	1810.2
			56.6	905.1
			226.3	1810.2
			5.0	40.0
			452.5	1810.2
			5.0	452.5

Administration arm	Sex	Subpopulation	Neutralizing antibody titer (XBB.1.5.6)	
			Day 1	Day 29
DS-5670d (cont.)	Female (cont.)	C (cont.)	452.5	2560.0
			226.3	1280.0
			640.0	1280.0
			452.5	2560.0
			2560.0	5120.0
			113.1	3620.4
			80.0	640.0
			452.5	905.1
			20.0	905.1
			113.1	3620.4
			5.0	320.0
		D	40.0	905.1
			14.1	5120.0
			5.0	5.0
			20.0	1810.2
			640.0	5120.0
			56.6	640.0
			5.0	2560.0
			5.0	5.0
			160.0	5120.0
BNT162b2	Male	A	56.6	640.0
			28.3	226.3
			226.3	5120.0
			226.3	1280.0
			20.0	1810.2
			905.1	3620.4
			226.3	905.1
			113.1	5120.0
			160.0	3620.4
			452.5	452.5
			113.1	5120.0
			226.3	2560.0
			320.0	1280.0
			56.6	2560.0
			28.3	320.0
			56.6	226.3
			113.1	5120.0
			113.1	3620.4
			905.1	5120.0
			113.1	3620.4
			56.6	2560.0
			80.0	1810.2
			113.1	1280.0
			40.0	640.0
			28.3	905.1
			226.3	2560.0
			320.0	3620.4
			226.3	2560.0
			113.1	1280.0
			28.3	452.5
			320.0	905.1
			113.1	226.3
			40.0	1280.0
			80.0	905.1
			452.5	1280.0
			56.6	1810.2
			452.5	1280.0
			640.0	2560.0
			1810.2	3620.4
			226.3	5120.0

Administration arm	Sex	Subpopulation	Neutralizing antibody titer (XBB.1.5.6)	
			Day 1	Day 29
BNT162b2 (cont.)	Male (cont.)	A (cont.)	113.1	3620.4
			1810.2	5120.0
			640.0	5120.0
			113.1	3620.4
			226.3	5120.0
			1280.0	2560.0
			905.1	5120.0
			640.0	1810.2
			2560.0	1810.2
			113.1	5120.0
			28.3	1810.2
			14.1	1810.2
			56.6	3620.4
			7.1	160.0
			226.3	905.1
			28.3	226.3
			452.5	1280.0
			10.0	905.1
			226.3	1280.0
			320.0	2560.0
			452.5	905.1
			226.3	905.1
			56.6	1810.2
			40.0	5120.0
			905.1	2560.0
			56.6	5120.0
		B	226.3	1280.0
			56.6	3620.4
			14.1	905.1
			14.1	1280.0
			5.0	1810.2
			40.0	1280.0
			160.0	1280.0
			5.0	905.1
			5.0	905.1
			7.1	452.5
			20.0	452.5
			10.0	905.1
			10.0	1810.2
			113.1	905.1
			5.0	2560.0
			5.0	226.3
			5.0	640.0
			7.1	2560.0
			20.0	3620.4
			226.3	1280.0
			226.3	3620.4
			113.1	2560.0
			80.0	1810.2
			14.1	640.0
			80.0	905.1
			640.0	3620.4
			40.0	905.1
			7.1	3620.4
			640.0	1810.2
			226.3	2560.0
			7.1	452.5
			5.0	1810.2
			113.1	1810.2
			56.6	1280.0

Administration arm	Sex	Subpopulation	Neutralizing antibody titer (XBB.1.5.6)	
			Day 1	Day 29
BNT162b2 (cont.)	Male (cont.)	B (cont.)	452.5	1280.0
			28.3	3620.4
			226.3	5120.0
			5.0	1280.0
			56.6	5120.0
		C	80.0	3620.4
			1810.2	2560.0
			452.5	1810.2
			905.1	5120.0
			226.3	5120.0
			56.6	640.0
			160.0	905.1
			56.6	1810.2
			5.0	452.5
			452.5	640.0
			10.0	452.5
			452.5	5120.0
			20.0	452.5
			10.0	640.0
			14.1	113.1
			5.0	7.1
			20.0	1280.0
			452.5	3620.4
			20.0	5120.0
			7.1	905.1
			113.1	1810.2
			113.1	5120.0
			640.0	5120.0
			5.0	5120.0
			640.0	3620.4
			320.0	3620.4
			5.0	640.0
			56.6	905.1
			10.0	1280.0
			56.6	1810.2
			5.0	452.5
			5.0	905.1
			1280.0	2560.0
			7.1	113.1
			5.0	28.3
			5.0	113.1
			226.3	2560.0
			226.3	640.0
			56.6	3620.4
			7.1	80.0
			905.1	2560.0
			28.3	640.0
			28.3	320.0
			640.0	905.1
			7.1	320.0
			113.1	3620.4
			226.3	2560.0
			56.6	905.1
			5120.0	5120.0
			1280.0	2560.0
			40.0	1280.0
			113.1	905.1
			40.0	3620.4
			452.5	3620.4
			113.1	5120.0

Administration arm	Sex	Subpopulation	Neutralizing antibody titer (XBB.1.5.6)	
			Day 1	Day 29
BNT162b2 (cont.)	Male (cont.)	C (cont.)	905.1	640.0
			226.3	2560.0
			160.0	640.0
			80.0	1810.2
			1280.0	2560.0
			28.3	320.0
			7.1	1810.2
			640.0	452.5
			20.0	320.0
			7.1	80.0
			7.1	640.0
			160.0	640.0
			5.0	1810.2
			14.1	2560.0
			5.0	7.1
			7.1	452.5
			28.3	1810.2
			905.1	905.1
			5.0	452.5
			5.0	80.0
			160.0	640.0
			10.0	320.0
			452.5	1280.0
			5.0	20.0
			14.1	640.0
			40.0	905.1
			80.0	1810.2
			226.3	640.0
			5.0	160.0
			80.0	905.1
			40.0	452.5
			113.1	452.5
			56.6	5120.0
			56.6	1810.2
			20.0	1280.0
			113.1	1810.2
			320.0	5120.0
			113.1	452.5
			5.0	113.1
		D	5.0	320.0
			28.3	3620.4
			226.3	2560.0
			5.0	1810.2
			5.0	20.0
			5.0	80.0
			20.0	5120.0
	Female	A	452.5	1810.2
			40.0	640.0
			113.1	320.0
			905.1	2560.0
			320.0	5120.0
			113.1	452.5
			160.0	2560.0
			56.6	905.1
			5.0	905.1
			113.1	1280.0
			905.1	1280.0
			14.1	905.1
			113.1	1810.2
			320.0	905.1

Administration arm	Sex	Subpopulation	Neutralizing antibody titer (XBB.1.5.6)	
			Day 1	Day 29
BNT162b2 (cont.)	Female (cont.)	A (cont.)	56.6	3620.4
			80.0	3620.4
			20.0	1810.2
			113.1	1280.0
			452.5	3620.4
			226.3	905.1
			226.3	3620.4
			226.3	905.1
			40.0	1280.0
			56.6	1280.0
			320.0	1280.0
			40.0	905.1
			56.6	1810.2
			28.3	1280.0
			160.0	5120.0
			320.0	452.5
			56.6	3620.4
			226.3	5120.0
			113.1	3620.4
			80.0	1810.2
			10.0	1810.2
			1280.0	2560.0
			2560.0	905.1
			640.0	1810.2
			905.1	2560.0
			452.5	640.0
			56.6	320.0
			80.0	3620.4
			40.0	640.0
			1810.2	5120.0
			640.0	2560.0
			226.3	3620.4
			113.1	905.1
			160.0	2560.0
			7.1	3620.4
			1810.2	3620.4
			640.0	5120.0
			160.0	452.5
			56.6	1810.2
			320.0	5120.0
			56.6	2560.0
			452.5	5120.0
			160.0	2560.0
			28.3	905.1
			160.0	2560.0
			56.6	5120.0
			56.6	320.0
			1810.2	5120.0
			56.6	640.0
			1280.0	3620.4
			113.1	5120.0
			56.6	2560.0
			226.3	5120.0
			226.3	5120.0
		B	160.0	3620.4
			14.1	2560.0
			160.0	2560.0
			5.0	905.1
			5.0	226.3
			226.3	5120.0

Administration arm	Sex	Subpopulation	Neutralizing antibody titer (XBB.1.5.6)	
			Day 1	Day 29
BNT162b2 (cont.)	Female (cont.)	B (cont.)	5.0	226.3
			452.5	905.1
			640.0	905.1
			10.0	905.1
			5.0	2560.0
			113.1	2560.0
			5.0	1810.2
			56.6	905.1
			10.0	1280.0
			56.6	5120.0
			14.1	1810.2
			7.1	905.1
			14.1	3620.4
			14.1	2560.0
			28.3	5120.0
			5.0	905.1
			56.6	5120.0
			40.0	2560.0
			56.6	5120.0
		C	80.0	905.1
			113.1	1810.2
			226.3	1810.2
			10.0	1280.0
			5.0	226.3
			5.0	320.0
			20.0	640.0
			5.0	160.0
			5.0	160.0
			20.0	113.1
			20.0	905.1
			5.0	320.0
			452.5	320.0
			20.0	3620.4
			10.0	226.3
			28.3	320.0
			14.1	905.1
			160.0	640.0
			80.0	905.1
			80.0	226.3
			113.1	5120.0
			40.0	226.3
			80.0	3620.4
			905.1	1280.0
			5.0	226.3
			113.1	5120.0
			10.0	160.0
			5.0	113.1
			5.0	56.6
			5.0	452.5
			14.1	320.0
			320.0	2560.0
			40.0	1280.0
			40.0	452.5
			20.0	3620.4
			5.0	905.1
			14.1	1810.2
			40.0	5120.0
			5.0	20.0
			56.6	2560.0
			14.1	905.1

Administration arm	Sex	Subpopulation	Neutralizing antibody titer (XBB.1.5.6)	
			Day 1	Day 29
BNT162b2 (cont.)	Female (cont.)	C (cont.)	56.6	3620.4
			20.0	3620.4
			10.0	640.0
			40.0	2560.0
			7.1	80.0
			56.6	1810.2
			1810.2	2560.0
			14.1	2560.0
			452.5	2560.0
			80.0	3620.4
			452.5	3620.4
			113.1	1810.2
			14.1	80.0
			5.0	452.5
			56.6	3620.4
			320.0	5120.0
			113.1	2560.0
			226.3	5120.0
			28.3	5120.0
			56.6	2560.0
			28.3	56.6
			226.3	905.1
			56.6	1810.2
			40.0	5120.0
			28.3	640.0
			80.0	56.6
			80.0	5120.0
			10.0	320.0
			5.0	80.0
			452.5	905.1
		D	5.0	113.1
			5.0	14.1
			14.1	1280.0
			160.0	5120.0
			20.0	452.5
