# Supplementary material for: Immunogenicity and safety of DS-5670d, an omicron XBB.1.5-targeting COVID-19 mRNA vaccine: A phase 3, randomized, active-controlled study
Source: PLoS Med. 2025 Oct 13;22(10):e1004499. doi: 10.1371/journal.pmed.1004499 (PMC12517495; doi:10.1371/journal.pmed.1004499)
Supplement: S5 Table — (PDF) [file pmed.1004499.s013.pdf]

S5 Table. Unsolicited AEs occurring in >1 participant in either group (safety analysis set).

MedDRA Preferred Term	DS-5670d (*N* = 393)		BNT162b2 (*N* = 384)	
	All causality	Study vaccine-related	All causality	Study vaccine-related
Nasopharyngitis	15 (3.8)	1 (0.3)	8 (2.1)	0
Injection site erythema	9 (2.3)	8 (2.0)	1 (0.3)	1 (0.3)
Injection site pain	6 (1.5)	6 (1.5)	1 (0.3)	1 (0.3)
Injection site pruritus	6 (1.5)	6 (1.5)	0	0
Headache	5 (1.3)	1 (0.3)	8 (2.1)	2 (0.5)
Pyrexia	5 (1.3)	2 (0.5)	2 (0.5)	0
COVID-19	4 (1.0)	0	4 (1.0)	0
Injection site induration	4 (1.0)	4 (1.0)	1 (0.3)	1 (0.3)
Injection site swelling	4 (1.0)	4 (1.0)	1 (0.3)	1 (0.3)
Influenza	3 (0.8)	0	3 (0.8)	0
Axillary pain	3 (0.8)	2 (0.5)	1 (0.3)	1 (0.3)
Gastroenteritis	3 (0.8)	0	1 (0.3)	0
Pharyngitis	3 (0.8)	0	1 (0.3)	0
Arthralgia	3 (0.8)	0	0	0

Dysmenorrhea	2 (0.5)	0	2 (0.5)	0
Nausea	2 (0.5)	2 (0.5)	1 (0.3)	0
Injection site hypoesthesia	2 (0.5)	2 (0.5)	0	0
Injection site warmth	2 (0.5)	2 (0.5)	0	0
Blood triglycerides increased	0	0	3 (0.8)	0
Abdominal pain	0	0	2 (0.5)	0
Pruritus	0	0	2 (0.5)	0
Urticaria	0	0	2 (0.5)	1 (0.3)

Data are shown as *n* (%). AE, adverse event; MedDRA, Medical Dictionary for Regulatory Activities.
